# Understanding the social determinants of Aedes-borne diseases in Iran: A qualitative exploration of challenges and policy solutions

**DOI:** 10.1371/journal.pntd.0013850

**Published:** 2025-12-22

**Authors:** Efat Mohamadi, Jawad Jafarzadeh, Fatemeh Mohamadi, Hakimeh Mostafavi, Ahad Bakhtiari, Ghobad Moradi, Maryam Sargolzaei Moghaddam, Mohammadreza Mobinizadeh, Fateme Yaftian, Amin Ghanbarnejad, Mehrnaz Mozafari, Enayatollah Homaie Rad, Ehsan Sheykh Noori, Marziyeh Najafi, Amirhossein Takian, Alireza Olyaeemanesh

**Affiliations:** 1 Health Equity Research Center (HERC), Tehran University of Medical Sciences, Tehran, Iran; 2 Iranian Research Network for Social Determinants of Health (IRNSDH), Tehran, Iran; 3 Social Determinants of Health Research Center, Research Institute for Health Development, Kurdistan University of Medical Sciences, Sanandaj, Iran; 4 Center for Communicable Disease Control (CDC), MOHME: Iran Ministry of Health and Medical Education, Tehran, Iran; 5 National Institute for Health Research (NIHR), Tehran University of Medical Sciences, Tehran, Iran; 6 Social Determinants in Health Promotion Research Center, Hormozgan University of Medical Sciences Faculty of Health, Hormozgan, Iran; 7 Vice Chancellor of Health, Bushehr University of Medical Sciences, Bushehr, Iran; 8 Social Determinants of Health Research Center, Trauma Institute, Guilan University of Medical Sciences, Rasht, Iran; 9 Department of Disease Prevention and Control, Health Deputy, Zahedan University of Medical Sciences, Zahedan, Iran; 10 TDR Grantee at IIHMR, Jaipur, India; 11 Centre of Excellence for Global Health (CEGH), Department of Global Health & Public Policy, School of Public Health, Tehran University of Medical Sciences (TUMS), Tehran, Iran; 12 Department of Health Management, Policy, and Economics, School of Public Health, Tehran University of Medical Sciences (TUMS), Tehran, Iran; Anesvad Foundation, SPAIN

## Abstract

**Background:**

Aedes-borne diseases pose escalating public health challenges globally, influenced not only by ecological and biological factors but critically by social determinants of health (SDH). In Iran, emerging local transmission of dengue highlights these diseases as effective proxies to examine the impact of social and environmental factors on health equity. However, evidence on context-specific drivers and effective responses remains scarce. This study aimed to identify key SDH and propose prioritized interventions to inform evidence-based policymaking.

**Methods:**

This mixed-method study included a two scoping review on SDH of Aedes-Borne Diseases and SDH-focused interventions, complemented by qualitative data from in-depth interviews with 21 national and provincial health experts in Iran. Data were analyzed using an inductive content analysis approach and MAXQDA 25 software was used throughout the analysis. The identified interventions were prioritized through a multi-criteria decision analysis, incorporating expert input via an online checklist and digital platform, based on four key criteria: effectiveness, feasibility, social acceptability, and political support.

**Results:**

Findings reveal that socioeconomic inequalities, weak community awareness, and limited health system capacity substantially drive disease risk. Notably, the local emergence and spread of dengue serve as a sensitive indicator reflecting broader social vulnerabilities affecting health outcomes. Integrated multisectoral strategies—encompassing health education, environmental management, digital surveillance, and cross-sector collaboration—are vital for effective control. Priority actions include healthcare worker training, embedding disease prevention within educational curricula, and tailored communication leveraging native languages and trusted community leaders.

**Conclusion:**

The study underscores that Aedes-borne diseases are not only biological threats but also reflections of underlying social and structural inequities. By framing dengue and related diseases as sentinel indicators of SDH, policymakers can better design integrated and equity-oriented strategies. Controlling Aedes-borne diseases requires a shift from disease-centric approaches toward comprehensive, SDH-informed strategies that strengthen community engagement, improve environmental and health infrastructure, and enhance cross-sector coordination. The prioritized interventions identified in this study provide a practical roadmap for strengthening preparedness and response in Iran and similar settings.

## Author summary

Aedes mosquitoes, which can spread dengue and other viral infections, are now appearing in several provinces of Iran. We wanted to understand how social and economic conditions, local environments, and the health system together shape the risk of these diseases and the success of control efforts. To do this, we reviewed international evidence and spoke with national and provincial health experts across affected regions in Iran. We then used a structured ranking process to identify which social factors and policy actions should be prioritized. Our findings show that poverty, poor housing and water management, limited public awareness, and gaps in health services all increase the risk of Aedes borne diseases. We also highlight practical, high priority actions, e.g., training health workers, integrating mosquito borne disease prevention into school curricula, and using local languages and trusted community leaders for communication. These results can help health authorities design fairer and more effective strategies to prevent future outbreaks in Iran and similar settings.

## Introduction

Aedes-borne diseases, i.e., dengue, chikungunya, and Zika have emerged as growing public health threats, particularly in tropical and subtropical regions [1]. Their rapid spread is driven by climate change, urbanization, global travel and trade, and broader social and economic factors [2,3]. According to the World Health Organization (WHO), by 2024, over 90 countries were affected by these diseases, with more than 7.6 million dengue cases and 3,000 related deaths reported, a significant increase compared to previous years [4,5]. The highest burdens have been observed in South American nations and Pacific island countries [5,6]. Data from the European Medicines Agency in the same year estimate a global case count of 13 million, with 8,500 deaths, most notably 11 million cases and 6,000 deaths reported from the PAHO region. Brazil alone accounted for over 9.5 million infections in 2024, followed by Argentina, Mexico, Paraguay, and Colombia [7]. These trends have been attributed to climate shifts, viral evolution, prolonged warm seasons, weaknesses in health systems, and increasing human and goods mobility.

A growing body of evidence indicates that Aedes-borne diseases are significantly influenced by social determinants of health (SDH). These include economic status, access to healthcare services, educational attainment, and overall living conditions [8–10]. Low health literacy, inadequate public education, and limited awareness about mosquito bite prevention and disease control measures are associated with increased vulnerability to Aedes-borne infections [11–14]. Populations living in areas with poor sanitation infrastructure,i.e., lack of access to safe drinking water and adequate hygiene facilities, are at heightened risk [15–17]. Furthermore, stagnant water, which serves as an ideal breeding ground for Aedes mosquitoes, is more frequently found in low-income and underserved communities [18–20].

In Iran, Aedes-borne diseases have been reported in at least nine provinces to date, with the majority of confirmed cases concentrated in the southern and southeastern regions, particularly in Sistan and Baluchestan, Hormozgan, and Kerman, mainly within urban and peri-urban setting [21]. Although current reports indicate a limited number of cases, early evidence suggests that these diseases hold significant potential for expansion across southern provinces. Factors, i.e., climate suitability, proximity to international transmission hotspots, and inadequate health and environmental infrastructure place these areas at heightened risk. Despite this growing concern, there remains a lack of comprehensive research in Iran exploring the social determinants that influence the prevention, control, and spread of Aedes-borne diseases.

Given the complex interplay of social determinants and the diverse contextual drivers of Aedes-borne diseases across regions [22–24], current evidence strongly highlights the need for comprehensive, multisectoral approaches to improve understanding and inform effective control strategies. In Iran, the rising number of reported cases in southern provinces, coupled with limited localized data, underscores a critical need to contextualize global insights and adapt successful international interventions to local conditions. This study addresses the significant knowledge gaps regarding how social determinants shape the transmission and control of Aedes-related diseases across different Iranian regions.

This study aims to identify the key social determinants affecting the prevention, control, and spread of Aedes-borne diseases in Iran and to develop context-specific, evidence-based policy recommendations. It synthesizes global and national findings, prioritizes effective SDH-focused interventions based on impact and feasibility, and assesses the infrastructure needed for successful, multisectoral implementation. This research is guided by the underlying theoretical framework that variations in social determinants, i.e., economic, environmental, and health system factors, significantly influence the risk and control feasibility of Aedes-borne diseases in Iran. This implicit hypothesis informed the design, analysis, and interpretation of our findings.

## Method

### Ethics statement

This study involved human participants. The qualitative component included in-depth interviews, for which Written informed consent was obtained from all participants prior to their participation in the study. Participants also provided consent for the publication of their interview content in a fully anonymized form, with all personally identifying information removed. Confidentiality was strictly maintained, and participants reviewed and confirmed their interview transcripts. The study protocol was approved by the Ethics Committee of Tehran University of Medical Sciences (Approval Code: IR.TUMS.SPH.REC.1403.255).

**Fig 1:**
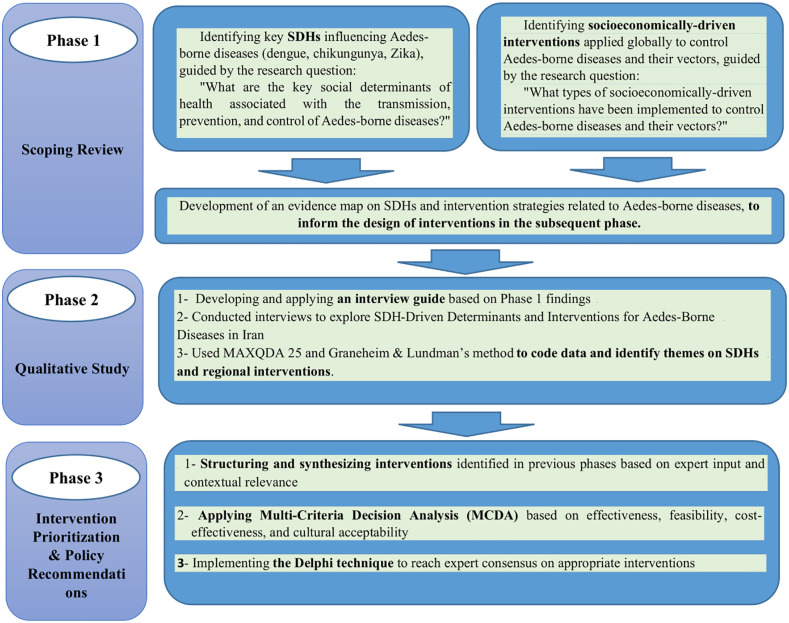
Overview of the mixed-methods study design.

This study is a mixed-methods applied research project in the field of health systems, designed and implemented in three sequential phases (Fig 1).

### Phase I: Scoping review to identify social determinants of health and related interventions

#### Study design.

The first phase of this research was conducted using a scoping review methodology, following the framework proposed by Arksey and O’Malley [25]. This phase aimed to identify key social determinants influencing the spread, prevention, and control of Aedes-borne diseases (including dengue, chikungunya, and Zika), as well as relevant interventions applied globally. To address the objectives, two distinct scoping reviews were conducted, each guided by a specific research question:

- What are the key social determinants of health SDH associated with the transmission, prevention, and control of Aedes-borne diseases (dengue, chikungunya, and Zika)?
- What types of socioeconomically-driven interventions have been implemented to control Aedes-borne diseases and their vectors?

#### Data sources and search strategy.

The literature search was conducted across three major electronic databases; PubMed, Cochrane Library, and Scopus. In addition, the websites of key international organizations, i.e., the WHO were reviewed to retrieve relevant guidelines. Search terms were developed using controlled vocabulary (e.g., MeSH terms) and keyword combinations related to SDH, vector-borne diseases, and intervention strategies (Table 1).

**Table 1:** Summary of search queries and focus areas of search study.

**Study 1:** Social SDH of Aedes-Borne Disease Transmission and Control	(“yellow fever”[MeSH Terms] OR “yellow fever”[Title/Abstract] OR “zika virus”[MeSH Terms] OR “zika”[Title/Abstract] OR “dengue”[MeSH Terms] OR “dengue”[Title/Abstract] OR “chikungunya virus”[MeSH Terms] OR “chikungunya”[Title/Abstract] OR “aedes”[MeSH Terms] OR “aedes”[Title/Abstract]) AND (“Social Determinants of Health”[MeSH Terms] OR “social factors”[Title/Abstract] OR “poverty”[MeSH Terms] OR “poverty”[Title/Abstract] OR “socioeconomic factors”[MeSH Terms] OR “income”[Title/Abstract] OR “low income”[Title/Abstract] OR “education”[MeSH Terms] OR “educational status”[Title/Abstract] OR “housing”[MeSH Terms] OR “housing conditions”[Title/Abstract] OR “environment”[MeSH Terms] OR “environmental exposure”[Title/Abstract] OR “climate”[MeSH Terms] OR “climate change”[Title/Abstract] OR “healthcare access”[Title/Abstract] OR “health facilities”[MeSH Terms])
**Study 2:** Interventions Based on SDHs for the Prevention and Control of Aedes-Borne Diseases	((“yellow fever” OR “Zika” OR “Aedes” OR “chikungunya” OR “dengue”) AND (“intervention” OR “interventions” OR “prevention” OR “preventions” OR “recommendation” OR “recommendations” OR “policy option” OR “policy options” OR “strategies” OR “strategy” OR “management” OR “control” OR “mitigation” OR “measures” OR “eradication” OR “surveillance” OR “vector control” OR “health programs

#### Inclusion criteria.

Studies were included if they met the following criteria: (1) published in English; (2) explicitly focused on the role of SDHs in the transmission, prevention, and control of Aedes-borne diseases; (3) addressed the design or implementation of policy and programmatic interventions based on SDH; (4) published between 2005 and December 30, 2024; (5) had full-text availability; and (6) included various study types, i.e., primary research, reviews, and guidelines.

Exclusion Criteria: Studies were excluded if they (1) were not written in English; (2) were letters to the editor or commentary pieces; (3) did not provide access to the full text; or (4) referred to interventions or their impacts in an unclear or non-transparent manner during the screening process.

Screening Process of Studies: The literature screening process was conducted in accordance with PRISMA guidelines [26]. A comprehensive search was performed in databases, i.e., PubMed, Scopus, and Web of Science using keywords related to Aedes-borne diseases and social determinants of health. After removing duplicates, titles and abstracts were independently screened by two reviewers based on predefined inclusion and exclusion criteria. Full-text articles were then assessed, and any disagreements were resolved through discussion or consultation with a third reviewer. The entire process was documented using a PRISMA flow diagram. During this stage, EndNote software was used to organize and manage the references.

#### Data analysis.

included studies underwent in-depth review. General information, i.e., authors, study type, year, setting, and country, was summarized in a study characteristics table. Specific content related to study objectives was synthesized using content analysis within MAXQDA 25 software.

#### Validity.

To ensure the validity of the screening and analysis process, two independent researchers reviewed each article for relevance and data extractability. Disagreements were resolved through discussion, and if consensus was not reached, a third reviewer was consulted.

### Phase II: Identification of social determinants of health (qualitative study)

#### Study design.

The qualitative component of this study was specifically designed to capture the perspectives of national and provincial health policymakers, managers, and technical experts. The aim was to explore the key SDHs influencing the transmission, prevention, and control of Aedes-borne diseases in Iran from the standpoint of those responsible for decision-making, program implementation, and policy development at systemic levels.

#### Participants.

Participants were selected through purposive sampling and included 21 key informants and experts. These comprised national-level policymakers from the Ministry of Health and Medical Education (MOHME), operational managers and field specialists from medical universities in affected provinces, and academic professionals. The range of expertise spanned public health specialists, i.e., epidemiologists, disease control officers, and environmental health experts, i.e., as well as entomologists, social psychologists, sociologists (focused on behavioral and cultural aspects of disease prevention), infectious disease specialists, family physicians directly involved in patient care, and researchers engaged in SDH-focused work across provinces.

#### Data collection tool.

Data were collected through semi-structured qualitative interviews guided by an interview protocol developed after the completion of Phase I. This guide was designed to ensure comprehensive coverage of the study’s core questions. To develop the interview framework, the research team first reviewed the study objectives and the key themes and indicators identified during the scoping review. A draft version of the interview guide was then created and refined based on input from academic advisors affiliated with the project (S1 Appendix). The finalized guide was used in all interviews to facilitate consistency and thematic depth.

#### Data collection.

Data were collected through semi-structured interviews. Given the geographic distribution of participants, interviews were conducted both in person and virtually. Prior to each interview, participants were contacted to provide a general overview of the research team, the study objectives, and the expected contribution of the participant. Following this, interviews were scheduled based on the participant’s preferred time and format. At the beginning of each session, the study’s goals were reiterated, and verbal consent for recording was obtained. After each interview, transcripts were prepared verbatim and shared with participants for verification and approval before inclusion in the analysis. The semi-structured format allowed participants ample space to express their perspectives freely. All interviews were audio-recorded, and supplementary notes were taken during each session. We conducted 12 individual in depth interviews and two focus group discussions comprising health managers, disease control specialists, and representatives from provincial health departments. The overall sample size (n = 21) was determined through purposive sampling and finalized at thematic saturation, whereby no new themes emerged from additional interviews or group discussions. Focus group composition allowed for interactive exchange of perspectives among experienced professionals, complementing individual interview insights”.

#### Data analysis.

Data were analyzed using an inductive content analysis approach, following the conventional content analysis method as proposed by Graneheim and Lundman [27]. Transcription and preliminary coding began immediately after each interview was completed and approved by the interviewee. To immerse in the data, transcripts were read multiple times, and meaning units—words, phrases, or paragraphs relevant to the study topic—were identified. These units were then condensed and labeled with appropriate codes. As coding progressed, similar codes were grouped into subcategories through constant comparison. These subcategories were further reviewed, compared conceptually, and organized into broader main categories. The coding and categorization processes were iterative and conducted by three members of the research team to ensure accuracy and analytical depth. To manage and organize the coding process, MAXQDA 25 software was used throughout the analysis. Themes were identified through systematic comparison and reflection on the categories, ensuring conceptual coherence and robustness of the final findings.

### Phase III: Contextualization and prioritization of SDH-based interventions

#### Study design.

This phase adopted a mixed-methods design grounded in participatory policy analysis and guided by a multi-criteria decision analysis (MCDA) approach [28,29]. The objective was to contextualize and prioritize SDH-oriented policy interventions for the prevention and control of Aedes-borne diseases, aligned with the national and provincial contexts of the affected regions.

#### Participants.

A total of 9 experts were engaged, including entomologists, public health officials, epidemiologists, community representatives, Non-Governmental Organization (NGO) delegates, and health professionals from relevant institutions, i.e., MOHME, provincial municipalities, universities, and provincial health departments.

#### Data collection.

Interventions identified through the systematic review and expert interviews in earlier phases were consolidated and categorized. These interventions were then evaluated using four criteria: effectiveness, feasibility of implementation, social acceptability, and political support. To collect expert opinions, an online checklist was developed and distributed via a digital platform (S2 Appendix and S1 File)*.* Experts were asked to assess each proposed intervention against the four criteria and assign weighted scores (from 1 to 10) based on the perceived importance of each dimension.

#### Data analysis.

The data collected in this phase were analyzed using a two-step quantitative approach to prioritize the SDH-based interventions. First, the Shannon Entropy Method [30] was applied to calculate the objective weights of the four predefined evaluation criteria: effectiveness, feasibility, social acceptability, and political support. We selected this method for criterion weighting as it enables an objective, data-driven assessment of dispersion and significance among expert responses, without the introduction of subjective biases that may occur with preference based approaches, i.e., the Analytic Hierarchy Process (AHP). Shannon Entropy is particularly suitable where the aim is to minimize subjective influence and to reflect the intrinsic diversity and informational content of available quantitative data. This enhances transparency and strengthens the reproducibility of the weighting procedure in our multi-criteria decision analysis framework. This method enabled the assignment of weights based on the dispersion of expert scores, ensuring minimal subjectivity in the weighting process. Subsequently, the Simple Weighted Average Method [31,32] was used to compute a composite score for each intervention, by multiplying the score of each intervention under each criterion by its respective weight and summing the results. This process provided a final ranked list of prioritized interventions tailored to the contextual needs of the country and the affected provinces. All calculations were performed using Microsoft Excel for entropy-based weight determination and SPSS version 26 for validation and descriptive analysis. Detailed scoring matrices, entropy values, and final ranking outputs are provided in S1 File.

## Results

### Key SDHs influencing Aedes-Borne diseases

A total of 206 records were initially retrieved through a comprehensive database search. After removing duplicates, non-English publications, and irrelevant or inaccessible studies, 99 articles met the inclusion criteria and were selected for full-text review and analysis (see PRISMA diagram in S3 Appendix). These studies [1–3,8–12,14–16,18–20,22,33–115] focused primarily on dengue (n = 54), Aedes mosquitoes in general (n = 28), Zika virus (n = 14), and chikungunya virus (n = 3), and were conducted across diverse geographic settings, with the highest contributions from Brazil, the United States, India, and Saudi Arabia. Most articles were published between 2015 and 2024 and employed cross-sectional, spatial (GIS), or review methodologies, reflecting the increasing global attention to Aedes-borne diseases.

**Fig 2:**
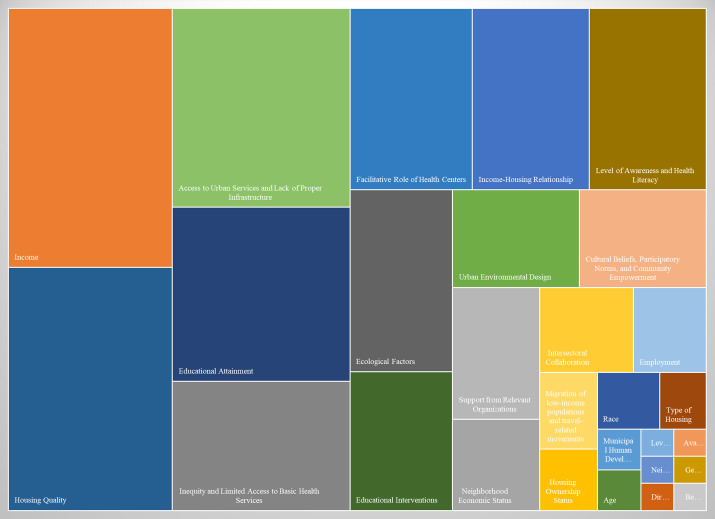
Frequency-based social determinants influencing the transmission and prevalence of aedes-borne diseases: a synthesis from study findings.

A comparative analysis of the frequency of SDHs associated with the spread, prevention, and control of Aedes-borne diseases identified three major contributing factors. The most frequently cited determinant was income, a subcomponent of the broader poverty and related socioeconomic indicators, mentioned in 48 studies. The second key factor was access to urban services and the lack of adequate infrastructure, part of the broader category of urban infrastructure and social support, noted in 40 studies. The third significant determinant was housing quality, falling under the broader housing and environmental conditions domain, referenced in 37 studies (Fig 2). Detailed findings and frequency distributions of the identified determinants are presented in the S4 Appendix.

### Policy recommendations derived from the scoping review on SDHs

As part of the identification of SDH-focused interventions, 4,944 English-language articles (2000–2024) were retrieved from major databases, along with 10 relevant WHO reports. After removing 583 duplicates, titles and abstracts were screened based on predefined inclusion criteria. Following full-text review, 71 studies and global documents were included in the final synthesis; (see PRISMA diagram in S5 Appendix) [12,116–185]. Most articles were published between 2019 and 2024 and covered dengue (n = 54), general Aedes-related issues (n = 28), Zika virus (n = 14), and chikungunya (n = 3). The studies originated from diverse settings, with the highest representation from Brazil, the U.S., India, Saudi Arabia, and international collaborations. Methodologies included original research (n = 41), reviews (n = 20), policy papers, and one protocol.

Findings indicate that combining multi-level strategies with intersectoral collaboration can significantly reduce the transmission of Aedes-borne diseases. However, the selection and effectiveness of interventions largely depend on the contextual and socio-environmental characteristics of the target setting. None of the reviewed studies identified a single “gold standard” intervention; rather, a combination of approaches across different domains proved more effective in enhancing both impact and sustainability. These strategies also included more advanced measures, i.e., regular monitoring of mosquito habitats, targeted spraying, and the use of innovative technologies for environmental management. Regional and international cooperation, i.e., data sharing and harmonized cross-border strategies, was emphasized as a critical element in preventing transnational disease spread. Overall, these interventions, through an integrated approach involving preventive and control actions, infrastructure improvements, and public awareness, provide a comprehensive foundation for sustainable and effective management of Aedes-borne diseases. Details of this finding provided in S6 Appendix*.*

### SDH-Driven determinants and interventions for Aedes-Borne diseases in Iran

A total of 21 participants were interviewed, including 12 individual in-depth interviews and two focus group discussions. The participants were selected with maximum variation in terms of organizational affiliation, educational level, professional role, gender, and years of experience (Table 2 and S7 Appendix).

**Table 2:** Descriptive characteristics of interview participants.

Variable	Statistic	Value
**Education**	Bachelor’s (%)	19.04
	Master’s (%)	9.53
	Doctorate (%)	71.43
**Gender**	Female (%)	38.1
	Male (%)	61.9
**Work experience (years)**	Mean (SD)	17.29 (8.61)
**Interview duration (minutes)**	Mean (SD)	41.14 (18.29)
	Range	18–100

Thematic analysis of the qualitative interviews revealed that the complexity of Aedes-borne diseases arises from a multifaceted interplay of social, economic, environmental, and entomological factors. Through this analysis, five main themes were identified: environmental factors, social factors, economic factors, health system infrastructure, and mosquito species characteristics. These primary themes encompassed 18 sub-themes (Tables 3). The full transcripts of the interviews are provided in S2 File.

**Table 3:** Classification of themes, subthemes, and codes related to social determinants influencing Aedes-borne diseases in Iran.

Subtheme	Code	Explanation
***Theme 1: Environmental Factors***		
**Climatic Conditions**	Climate Change	Global warming has created new habitats for mosquitoes.
	High Humidity	High humidity in northern forested areas provides more natural breeding sites.
	Water Shortage in South	Water storage in tanks and containers creates larval habitats in southern areas.
**Larval Habitats**	Stagnant Water	Water collected in containers, air coolers, flower pots, etc., serves as breeding sites.
	Natural Habitats	Dew on plants and elevator shafts offer natural larval breeding environments.
**Geographic Differences**	Northern Habitats	In the north, due to heavy rainfall, more natural larval habitats are present.
	Southern Habitats	In the south, artificial habitats are created due to water storage practices.
**Human Behavior**	Over-Irrigation	Excessive watering leads to stagnant water in pots and yards, supporting mosquitoes.
***Theme 2: Social Factors***		
**Health Literacy**	Low Awareness on Prevention	Lack of knowledge about mosquito breeding sites and prevention strategies increases the risk of disease transmission.
	Role of Education	Targeted education plays a significant role in controlling larval habitats and preventing disease spread.
**Population Density**	Increased Mosquito Contact	High-density areas have greater exposure to mosquito bites and thus a higher risk of infection.
**Culture of Cooperation**	Role of Local Leaders	In smaller or traditional communities, local leaders can significantly influence community behavior toward disease prevention.
	Cultural Differences	Variations in local culture affect the acceptance and effectiveness of prevention and control measures.
**Access to Health Services**	Dependence on Public Health	Low-income groups rely more on the public health system and tend to cooperate better with public health interventions.
	Trust in Health System	Levels of trust in the health system vary across communities and directly impact their willingness to participate in control programs.
***Theme 3: Economic factors***		
**Income Level**	Economic Poverty	Poverty leads to the use of unsafe containers for water storage, creating breeding grounds for mosquitoes.
	Limited Access to Services	Economically disadvantaged individuals have less access to health and diagnostic services.
**Cost of Treatment**	Self-Medication	Due to high treatment costs, low-income populations are less likely to seek professional care, contributing to disease spread.
	Delayed Healthcare Seeking	Financial barriers cause delays in seeking medical attention, increasing the risk of complications and transmission.
**Housing Conditions**	Overcrowding	Small, crowded houses create more favorable environments for mosquito breeding.
	Poor Health Infrastructure	Low-income areas often lack proper sewage systems and reliable water supplies, leading to increased larval habitats.
***Theme 4: Health system Infrastructure***		
**Health Facilities**	Lack of health centers in remote areas	Limited access to primary care in underserved southern regions.
	Regional disparities in access	Northern areas have stronger infrastructure than southern regions.
**Medical Equipment**	Shortage of diagnostic tools	Lack of diagnostic kits and labs delays timely detection and control.
**Human Resources**	Lack of trained personnel	Shortage of doctors and public health experts in deprived areas.
	Uneven distribution of staff	Healthcare workers are concentrated in urban areas, leaving rural gaps.
**Active Health Programs**	Absence of active surveillance	No systematic disease detection mechanisms in many high-risk areas.
	Inadequate staff training	Local health workers lack specialized training for vector-borne disease control.
***Theme 5: Mosquito Species Characteristics***		
**Species Variation**	*Aedes albopictus*	Typically found in northern, humid regions of the country.
	*Aedes aegypti*	Common in southern, hot and arid areas.
**Biological Behavior**	Anthropophilic behavior	*Aedes aegypti* shows stronger preference for feeding on humans.
	Ecological adaptability	Both species can adapt to a wide range of habitats.
**Habitat Patterns**	Artificial habitats	Thrive in stagnant water containers and urban environments.
	Natural habitats	*Aedes albopictus* often breeds in natural environments, i.e., plant containers.

The spread of Aedes-borne diseases in Iran is shaped by the interaction of environmental, social, economic, health system, and entomological factors. Climate variability, water scarcity, and humidity foster mosquito breeding, while social and economic inequities, i.e., low health literacy, overcrowding, and poor access to healthcare, intensify vulnerability and delay disease control. Structural gaps in diagnostic capacity, surveillance, and trained personnel, especially in the southern provinces, further constrain timely response. Entomological data reveal that Aedes aegypti dominates arid southern regions, relying on human hosts and artificial habitats, whereas Aedes albopictus prevails in humid northern zones with broader ecological adaptability. (Table 3).

### Policy strategies targeting SDHs for the prevention and control of Aedes-Borne diseases in Iran

Interventions, i.e., public education and management of both artificial and natural breeding sites were ranked as high priority due to their direct role in reducing larval habitats and preventing disease transmission. In contrast, measures with more indirect but nonetheless important effects, i.e., the use of local media and multicultural communication programs, were classified as medium priority. The analysis emphasized that understanding the biological variations of Aedes species and aligning intervention programs with the cultural and social context of each region are critical for effective implementation. A summary of key policy strategies addressing social determinants for Aedes-borne disease prevention and control is provided in Table 4, with detailed thematic breakdowns available in S8 Appendix.

**Table 4:** Policy strategies targeting social determinants to prevent and mitigate the spread of Aedes-borne diseases in Iran.

Proposed Interventions	Rationale	Advantages	Limitations
***Main Theme 1: Health Education & Awareness***			
Implementing educational campaigns in schools and mosques to teach mosquito bite prevention and larval habitat management.	Limited public knowledge on disease prevention and the role of individual behaviors in disease control.	Enhances public awareness and community engagement in disease control.	Requires sustained financial resources and trained implementation teams.
Training local leaders to promote behavioral change.	Influential role of local leaders in gaining trust and influencing community behavior.	Facilitates acceptance of interventions at the community level.	Time-consuming process to gain active participation of local leaders.
***Main Theme 2: Environmental Improvement***			
Managing and draining stagnant water from containers, air coolers, and water tanks.	Stagnant water as the main breeding habitat for vector mosquitoes.	Reduces larval habitats and prevents disease transmission.	Limited household cooperation and insufficient oversight.
Developing and improving urban and rural drainage systems.	Poor drainage infrastructure in high-risk areas leads to water accumulation.	Reduces artificial breeding sites and improves environmental hygiene.	High implementation costs and requires long-term planning.
***Main Theme 3: Health Infrastructure***			
Expanding health facilities in remote and high-risk areas.	Limited access to healthcare services in underserved rural regions.	Reduces geographic disparities and facilitates early treatment of suspected cases.	Requires sufficient human and financial resources.
Equipping health centers with advanced diagnostic tools.	Increase in suspected cases and the need for accurate and rapid diagnosis.	Enhances diagnostic capacity and reduces disease identification time.	High cost of equipment and staff training needs.
***Main Theme 4: Economic Interventions***			
Providing subsidies for purchasing hygienic water storage containers.	Low-income households cannot afford proper containers, increasing larval habitats.	Reduces mosquito breeding sites and improves personal and environmental hygiene.	Limited financial resources to support adequate subsidies.
Creating job opportunities through environmental cleanup and waste management projects.	Need for environmental improvement and reducing unemployment in high-risk areas.	Simultaneously improves environmental health and local economic conditions.	Requires careful supervision to prevent resource misuse.
***Main Theme 5: Social Factors***			
Forming local volunteer groups to eliminate breeding sites and monitor the environment.	Low participation in environmental hygiene and neglect of breeding sites.	Enhances community involvement and social oversight.	Requires ongoing coordination and management of volunteer groups.
Utilizing local social media platforms for rapid information dissemination.	High internet access and social media use in urban and some rural areas.	Increases speed and reach of health messaging.	Limited internet access in some rural areas and high cost of producing targeted content.
***Main Theme 6: Cultural Factors***			
Organizing cultural events focused on disease prevention and mosquito control.	Local traditions and community events are effective in mobilizing social participation.	Enhances public awareness and community engagement.	Time-intensive and requires funding for event organization.
Engaging religious and social leaders to promote trust in public health interventions.	Strong influence of religious and social leaders on community behaviors.	Increases public trust and acceptance of health programs.	Some leaders may resist changes or consider the interventions insufficient.

**Fig 3:**
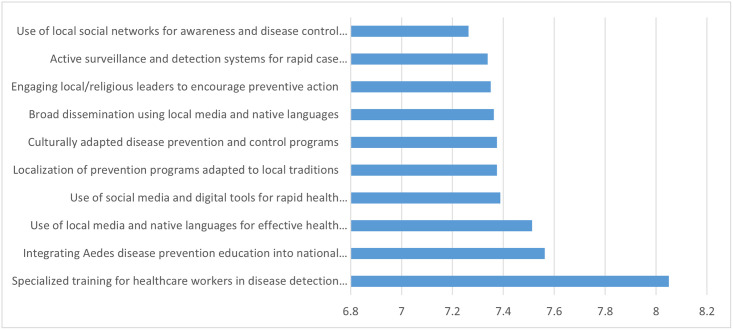
Priority ranking of top ten interventions for aedes-borne disease control in iran based on weighted multi-criteria decision analysis (based on weighted mean (0-10)).

The intervention prioritization analysis revealed varying scores across the four criteria: effectiveness (mean range: 6.25–9.13), feasibility (3.5–8.38), social acceptability (5.38–8.75), and political acceptability (4.75–8.13). Among these, feasibility showed the greatest variability, while social and political acceptability demonstrated moderate variation. Based on weighted average scores, the top three prioritized interventions were: (1) specialized training for healthcare workers to enhance disease identification and control skills (8.05); (2) integration of Aedes-borne disease prevention into the national education curriculum (7.56); and (3) use of local media and native languages for effective health communication (7.51). Similarly, Shannon entropy analysis yielded comparable but not identical priorities. This method highlighted: (1) training healthcare staff (8.19), (2) using local media and native languages (7.52), and (3) engaging local and religious leaders to promote preventive action (7.37) as the top-ranked interventions (Fig 3 and S9 Appendix).

## Discussion

This qualitative study explored the social determinants influencing the transmission, prevention, and control of Aedes-borne diseases in Iran. The findings revealed that environmental, economic, social, and health system factors play a critical role in the spread of dengue, chikungunya, and Zika. These results align with international evidence, highlighting the need for a multisectoral approach to effectively manage these diseases.

Environmental determinants, particularly climate variability, temperature, rainfall, and the presence of stagnant water, emerged as key contributors to the proliferation of Aedes mosquitoes. Higher temperatures and humidity accelerate mosquito development and expand their geographic range, consistent with findings from Brazil and Thailand, where a 1–2°C increase significantly raised mosquito density and disease transmission [2]. Similarly, rainfall contributes to ideal breeding conditions, as evidenced in Malaysia and Indonesia, where higher precipitation correlates with increased dengue incidence [33]. In Iran, participants from southern provinces, i.e., Hormozgan, Bushehr, and Sistan and Baluchestan reported that sudden downpours, coupled with poor surface water management, have intensified mosquito proliferation in those regions.

International studies have shown that economic deprivation and social inequalities have a direct impact on the incidence and control of Aedes-borne diseases [150]. In low-income countries, lack of access to basic health infrastructure, inadequate waste management, and high population density in impoverished areas have contributed to the increased transmission of dengue and chikungunya [97]. Consistent with these findings, the present study revealed that in Iran, low-income and underserved regions experience the highest exposure to Aedes-borne diseases. In these areas, poor waste collection, absence of proper sewage systems, and limited access to safe water create favorable conditions for mosquito breeding. These findings underscore the need to complement health interventions with broader social and economic measures, i.e., improving urban infrastructure and addressing economic disparities, to achieve effective disease control.

This study highlights the critical role of public awareness and community engagement in the success of Aedes-borne disease control programs. In countries, i.e., Singapore, large-scale educational campaigns have led to a significant decline in disease incidence [144]. However, in Iran, the absence of structured educational programs and low public awareness remain major barriers. Interviewees noted limited knowledge among the general population about disease risks and preventive practices, i.e., eliminating stagnant water and using protective screens. These findings align with global evidence that school-based programs, mosque outreach, social media, and national broadcasting can effectively enhance public engagement [22,36,144,145]. Countries like Malaysia, Brazil, and Cuba have also demonstrated the value of community-based approaches, including the active involvement of women and families in vector control [22,36,123,171]. While Iran has a broad network of mosques and schools offering strong outreach potential, the lack of sustained programming and insufficient funding hinder progress. Unlike Singapore, where health education is embedded in curricula, Iran has yet to establish a nationwide framework for Aedes-focused public education.

The study also highlighted weaknesses in the health system, including delays in disease detection and a shortage of diagnostic tools, as major challenges in Iran’s vector control efforts. This is consistent with findings from Malaysia and the Philippines, where inadequate surveillance systems, late case detection, and limited trained personnel have been linked to increased transmission and reduced effectiveness of control strategies [48]. In Iran, the shortage of trained medical entomologists, lack of coordination across health sectors, and financial constraints in procuring diagnostic equipment and insecticides were among the main operational challenges identified. Evidences suggest that the establishment of digital surveillance systems, strengthening of local health networks, and increased investment in diagnostic infrastructure can significantly reduce the incidence of Aedes-borne diseases [131,132]. Accordingly, reinforcing Iran’s health system infrastructure and developing robust disease monitoring and surveillance mechanisms should be considered key priorities for effective vector control.

Waste management and environmental sanitation are also essential components of Aedes control. In Brazil, strict regulations on tire disposal and scrap yard oversight have proven effective, while urban drainage programs in Mexico have contributed significantly to dengue prevention [2]. In Iran, similar interventions, i.e., community cleanup campaigns and improved waste management systems, were prioritized, but implementation remains hindered by inadequate municipal coordination, limited funding, and insufficient public participation. Unlike Brazil and Mexico [41,111], where incentive-based mechanisms promote community engagement, Iran has yet to operationalize such tools effectively.

Advanced surveillance and early warning systems are critical for anticipating outbreaks. Malaysia and Thailand have employed satellite data, artificial intelligence, and biosensors to forecast Aedes activity and identify high-risk zones [48]. These tools enable preemptive health responses. Additionally, rapid diagnostic testing (RDT) has enhanced early case detection in several countries. In contrast, Iran lacks a robust environmental surveillance and early warning system. While prioritized, implementation is constrained by limited technological infrastructure and budget. The use of locally developed digital platforms could offer a cost-effective solution.

Cross-sectoral collaboration is another key enabler of effective disease control. In Brazil, ministries of health, environment, and local governments coordinate efforts through an integrated governance structure [103]. In Cuba, civil society organizations, volunteers, and local health workers collaborate through unified networks [171]. In Iran, while intersectoral coordination is recognized as a high-priority intervention, it remains a major challenge. Fragmentation among ministries and agencies, particularly the lack of cooperation between the MOHME, municipalities, and the Department of Environment, undermines the consistency and effectiveness of intervention programs. Establishing formal coordination structures and joint policy frameworks is essential for enhancing implementation outcomes.

Studies from Brazil have emphasized the critical role of coordinated action among governmental and non-governmental bodies, intersectoral collaboration, and unified policy implementation in curbing the spread of Aedes-borne diseases [103]. Similarly, this study identified a lack of coordination among key institutions in Iran as a major barrier to effective program implementation. These findings highlight the necessity of establishing integrated management structures, enhancing intersectoral collaboration, and formulating cohesive policies. Global experiences demonstrate that successful strategies typically combine public education, environmental infrastructure development, health system strengthening, and cross-sector partnerships. Iran’s prioritized interventions, developed through expert consultation and scientific criteria, generally align with these approaches. However, challenges related to implementation capacity, institutional coordination, investment levels, and limited adoption of digital tools reveal the need for tailored, context-specific adaptation of global best practices.

This study offers a novel social perspective on the emergence and spread of Aedes-borne diseases in Iran for the first time, combining evidence from global literature with insights from diverse national stakeholders. A key strength lies in the integration of qualitative interviews with provincial managers and experts, enhancing the contextual relevance and depth of the findings. The study also employed flexible communication methods and leveraged existing networks within the MOHME and NGOs to overcome challenges in reaching key informants. However, a key limitation of this study is the absence of direct community level representation among participants. While our sample included national and provincial health experts, policymakers, and field specialists to capture policy and structural perspectives, community members and affected populations were not directly engaged in data collection. This omission may limit insights into grassroots determinants and the lived experiences of those most affected by Aedes-borne diseases. To mitigate this, the study relied on expert input from provincial representatives. Future research should incorporate community stakeholders to enable richer triangulation of social determinants and to better inform context-appropriate policy interventions. We acknowledge that the purposive expert sampling approach entails inherent limitations, including potential selection bias and constrained transferability of findings. This methodological choice was intended to capture in depth insights from experienced professionals, aligning with the study’s policy focused objectives. Nevertheless, while the results provide valuable contextual evidence, their application in other settings should be approached with caution and adapted to local social, environmental, and institutional conditions.

## Conclusion

This study highlights the multifaceted nature of Aedes-borne diseases, i.e., dengue, chikungunya, and Zika, in Iran, shaped by a complex interplay of social, economic, environmental, and health system factors. It convincingly links socioeconomic inequality, low health literacy, and limited health infrastructure with increased risk of Aedes-borne diseases in Iran. Importantly, the multi-criteria prioritization quantitatively highlights that socioeconomic factors carry the greatest relative weight, followed by health system capacity and environmental determinants. This evidence underscores the need to focus interventions on socioeconomic improvements alongside health system strengthening to effectively mitigate disease risk.

Public awareness and community engagement emerged as central to effective prevention. In Iran, leveraging schools, mosques, and local media, alongside improved collaboration between communities and public institutions, are crucial. Without behavioral change and health literacy, other interventions face limited impact. Additionally, upgrading environmental and health infrastructure, i.e., waste management, water systems, and diagnostic capacity, is vital for vector control. Strengthening health surveillance systems and intersectoral coordination between the MOHME, municipalities, environmental agencies, and civil society is critical. Lessons from countries like Brazil and Malaysia demonstrate the value of aligning health policies with environmental and development agendas. Based on the findings, the study recommends that policymakers focus on three key pillars:

1. Expanding public education and awareness campaigns through schools, mass media, and digital platforms.
2. Improving environmental infrastructure, with emphasis on water management, waste reduction, and efficient environmental monitoring.
3. Strengthening the health system through broader service access, rapid diagnostic tools, and enhanced disease surveillance.

Future research should explore the effectiveness of localized control models, assess the role of community participation, analyze the climate-related dynamics of Aedes spread in Iran, and examine innovative monitoring and vector control strategies.

## Supporting information

S1 Appendix Topic guide for qualitative phase. (DOCX)

S2 Appendix Prioritization form for SDH-oriented interventions and strategies for Aedes-Borne diseases in the Islamic Republic of Iran. (DOCX)

S3 Appendix PRISMA flow diagram – Search, screening, and selection of articles on key Social Determinants of Health (SDHs) influencing Aedes-Borne diseases. (DOCX)

S4 Appendix Frequency of Social Determinants of Health (SDHs) for Aedes-Borne diseases based on reviewed studies. (DOCX)

S5 Appendix PRISMA flow diagram – Search, screening, and selection of studies on SDH-based interventions related to aedes disease control. (DOCX)

S6 Appendix Interventions identified from the literature categorized by social and economic factors. (DOCX)

S7 Appendix Profile of participants in the qualitative component of the study. (DOCX)

S8 Appendix Policy strategies targeting SDHs for the prevention and control of Aedes-Borne diseases in Iran. (DOCX)

S9 Appendix Prioritization of identified interventions based on defined criteria. (DOCX)

S1 File Details of interventions’ prioritization. (XLSX)

S2 File Full transcripts of qualitative interviews with key informants, including provincial and national stakeholders. (DOCX)
