# Supplementary material for: Understanding the social determinants of Aedes-borne diseases in Iran: A qualitative exploration of challenges and policy solutions
Source: PLoS Negl Trop Dis. 2025 Dec 22;19(12):e0013850. doi: 10.1371/journal.pntd.0013850 (PMC12753069; doi:10.1371/journal.pntd.0013850)
Supplement: S1 Appendix — (DOCX) [file pntd.0013850.s001.docx]

**Appendix 1: Topic guide for qualitative phase**

| **General Information of the Interviewee** | |
| --- | --- |
| Name (Optional) |  |
| Field of Expertise / Occupation |  |
| Level of Education |  |
| Region |  |
| Duration of Experience in the Relevant Field |  |
| **Environmental Factors Identification** | |
| What natural environmental factors (e.g., climate, vegetation cover, stagnant water sources, water and sewage systems, urban design, waste management, temperature, humidity) contribute to the spread of Aedes mosquitoes? | |
| Does the type of housing (e.g., construction materials, urban fabric, etc.) affect the issue under investigation? | |
| Are there stagnant water sources in your area? | |
| In your opinion, has climate change had any impact? | |
| Please name any other environmental factors that have influenced the emergence and spread of Aedes-borne diseases. (If necessary, describe how these factors exert their influence.) | |
| What environmental interventions and involvement of which institutions are key to improving environmental conditions for the success of related programs? | |
| **socio-economic Factors** | |
| Is the general public sufficiently aware of the preventive measures against Aedes-borne diseases? | |
| Is the level of awareness among relevant institutions (such as municipalities, etc.) adequate? | |
| Have travel and migration had any impact on the spread of Aedes mosquitoes? | |
| Do poverty or limited resources affect disease control? If yes, how? | |
| What social factors (e.g., level of education, occupation, culture, health literacy, etc.) contribute to the spread of the disease? | |
| What socio-economic interventions and engagement of which institutions are essential to effectively respond to this situation? | |
| Are the existing health and medical infrastructures adequate? | |
| What are the main challenges related to health and medical infrastructure in the prevention and control of Aedes-borne diseases? | |
| In your opinion, what health-related policies or programs should be implemented? | |
| Has there been any successful experience in your region regarding the implementation of intersectoral interventions for Aedes mosquito control? Please explain. | |
| In your opinion, what is the most effective strategy for controlling the vector and the diseases it transmits? | |
| Please feel free to share any additional insights you believe may be helpful in this regard. | |
| Health Care Infrastructure | |
| Are the existing health and medical infrastructures sufficient? | |
| What are the main challenges related to health and medical infrastructure in the prevention and control of Aedes-borne diseases? | |
| In your opinion, what health policies or programs should be implemented? | |
| Experiences and Recommendations | |
| Has there been any successful experience in your region regarding the implementation of intersectoral interventions for Aedes mosquito control? Please describe. | |
| In your opinion, what is the most effective strategy for controlling the vector and the diseases it transmits? | |
| Additional explanation | |
| Please share any additional comments or observations you believe may be helpful in this regard. | |
