# Supplementary material for: Understanding the social determinants of Aedes-borne diseases in Iran: A qualitative exploration of challenges and policy solutions
Source: PLoS Negl Trop Dis. 2025 Dec 22;19(12):e0013850. doi: 10.1371/journal.pntd.0013850 (PMC12753069; doi:10.1371/journal.pntd.0013850)
Supplement: S2 Appendix — (DOCX) [file pntd.0013850.s002.docx]

**Appendix 2: Prioritization Form for SDH-Oriented Interventions and Strategies for Aedes-Borne Diseases in the Islamic Republic of Iran**

| **Evaluation Criteria** | **Definition** | **Importance** |
| --- | --- | --- |
| Effectiveness | The extent to which the intervention reduces disease incidence and controls vectors after implementation. | High |
| Feasibility | The practicality of implementing the intervention considering available resources, required expertise, and the country’s operational structures. | High |
| Social Acceptability | The level of acceptance of the intervention by the community, local groups, and within the region’s cultural context. | Moderate to High |
| Political & Institutional Support | The degree of support from government and decision-making bodies for the intervention’s implementation; level of required coordination between sectors such as health, environment, municipality, and education. | Moderate to High |
| Colleagues are welcome to express their views regarding the importance of each criterion (High, Moderate to High, Moderate, Moderate to Low, Low). | | |

Prioritization of Environmental Interventions and Strategies Based on the Control of Social Determinants to Prevent and Mitigate the Risk of Aedes-Borne Disease Outbreaks Identified in the Islamic Republic of Iran

| **Main category: Education and Awareness** |
| --- |
| Organizing educational campaigns in schools, mosques, and media to raise public awareness |
| Providing specialized training for health workers to strengthen skills in disease identification and control |
| Engaging religious and community leaders to enhance social acceptance and promote behavioral change |
| Utilizing local media to disseminate information related to disease control |
| Developing targeted educational programs for vulnerable groups such as housewives and children |
| **Environmental Improvement** |
| Managing and draining stagnant water from containers, beneath air coolers, and storage tanks to reduce larval habitats |
| Expanding and improving drainage systems in urban and rural areas |
| Implementing waste management systems and launching public clean-up campaigns to prevent garbage accumulation |
| Developing sewerage networks and applying sanitary solutions for managing open sewage in underserved areas |
| **Health Infrastructure** |
| Increasing the number of healthcare centers in remote and high-risk areas to improve access to medical services |
| Equipping healthcare facilities with advanced laboratory tools for rapid and accurate diagnosis of suspected cases |
| Establishing active case-finding systems to promptly identify infections and prevent further disease transmission |
| Improving the referral system for patients from rural areas to well-equipped healthcare centers |
| **Economic Interventions** |
| Providing subsidies for the purchase of health-related supplies such as appropriate water storage containers and protective nets |
| Reducing healthcare costs for low-income patients through free or subsidized services |
| Creating local employment opportunities through environmental clean-up and waste management projects |
| **Social Factors** |
| Forming local volunteer groups to eliminate larval habitats and monitor environmental conditions |
| Promoting public health literacy through targeted educational and informational programs |
| Utilizing local social networks to enhance awareness and ensure rapid dissemination of disease control information |
| Gaining support from social and religious leaders to improve acceptance of interventions and facilitate preventive measures |
| Strengthening intersectoral collaboration between government agencies and communities for disease control and implementation of environmental interventions |
| **Cultural Factors** |
| Localizing prevention programs and adapting them to traditional practices to enhance intervention effectiveness |
| Utilizing local media and native languages for effective and wide-reaching communication |
| Organizing cultural events and local ceremonies centered on raising awareness about disease prevention and control |
| Strengthening the role of religious and community leaders in delivering health messages and promoting the adoption of preventive measures |

Prioritization of Interventions and Large-scale Policy Recommendations Based on the Control of Social Determinants for the Prevention and Risk Reduction of Aedes-Borne Disease Outbreaks Identified in the Islamic Republic of Iran

| **Policy Area: Education and Awareness** |
| --- |
| Integrate education on the prevention of Aedes-borne diseases into the national education system, from primary schools to universities. |
| Establish public education networks through local and digital media to ensure effective and widespread information dissemination. |
| Develop targeted educational programs for high-risk groups and vulnerable communities. |
| Strengthen the role of religious and community leaders in public communication to improve social acceptance of interventions |
| **Policy Area: Environmental Improvement** |
| Implement comprehensive programs for managing stagnant water and improving urban and rural drainage systems. |
| Introduce financial incentives for households and businesses to support environmental improvements and reduce mosquito breeding sites. |
| Strengthen urban waste collection and management systems, with a focus on high-risk areas. |
| **Policy Area: Health Infrastructure** |
| Increase the number of health and medical centers in high-risk and remote areas. |
| Equip healthcare facilities with advanced diagnostic and treatment technologies for Aedes-borne diseases. |
| Establish surveillance systems and active case-finding mechanisms for rapid detection of infections. |
| Improve the patient referral system from rural areas to well-equipped healthcare centers. |
| **Policy Area: Economic Interventions** |
| Provide subsidies and financial support for the purchase of health-related equipment such as protective nets and water storage containers. |
| Reduce healthcare costs for low-income patients through the provision of free or subsidized services. |
| Create employment opportunities in waste management and environmental improvement sectors to promote both economic and public health outcomes simultaneously. |
| **Policy Area: Social Factors** |
| Establish local volunteer groups to eliminate mosquito breeding sites and engage in disease control efforts. |
| Utilize social media and digital technologies to facilitate rapid dissemination of health-related information. |
| Engage local and religious leaders to encourage community participation in preventive actions. |
| Strengthen intersectoral collaboration between government agencies, environmental bodies, and the health sector for effective disease control |
| **Policy Area: Cultural Factors** |
| Localize disease prevention and control programs to align with cultural norms and practices. |
| Use local media and native languages to ensure effective health communication. |
| Organize cultural events and local gatherings focused on raising awareness about Aedes-borne diseases. |
| Strengthen the role of religious and community leaders in delivering health messages and promoting community engagement. |

| If there are any interventions, strategies, or policy options not mentioned in the above tables that you believe should be considered, please specify them in the space below: |
| --- |
