# Supplementary material for: Understanding the social determinants of Aedes-borne diseases in Iran: A qualitative exploration of challenges and policy solutions
Source: PLoS Negl Trop Dis. 2025 Dec 22;19(12):e0013850. doi: 10.1371/journal.pntd.0013850 (PMC12753069; doi:10.1371/journal.pntd.0013850)
Supplement: S3 Appendix — (DOCX) [file pntd.0013850.s003.docx]

**Appendix 3: PRISMA Flow Diagram – Search, Screening, and Selection of Articles on Key Social Determinants of Health (SDHs) Influencing Aedes-Borne Diseases**

Studies screened & exclusion of criteria applied (n=176)

Record identified through database searching (n=206)

Exclusion of duplicates (n=30)

Record excluded (n=50)

Identification

Screening

Eligibility

Full-text studies assessed for eligibility (n=126)

Eligible studies identified for review (n=99)

Full-text excluded, with reasons (n=27)

Included
