# Supplementary material for: Understanding the social determinants of Aedes-borne diseases in Iran: A qualitative exploration of challenges and policy solutions
Source: PLoS Negl Trop Dis. 2025 Dec 22;19(12):e0013850. doi: 10.1371/journal.pntd.0013850 (PMC12753069; doi:10.1371/journal.pntd.0013850)
Supplement: S4 Appendix — (DOCX) [file pntd.0013850.s004.docx]

**Appendix 4: Frequency of Social Determinants of Health (SDHs) for Aedes-Borne Diseases Based on Reviewed Studies**

| **Row** | **Main Determinant** | **Sub-indicator** | **Examples** | **Reference Number** | **Frequency** |
| --- | --- | --- | --- | --- | --- |
| 1 | Poverty and Related Indicators | Municipal Human Development Index (MHDI) | This index includes sub-indicators for life expectancy, education, and income. | (27, 76) | 2 |
|  |  | Income | Higher-income households had access to amenities such as elevated water tanks, washing machines, and more frequent use of sealed containers.  Additionally, swimming pools were more common. In contrast, lower-income households showed greater motivation to control mosquitoes but had less knowledge, fewer resources, and poorer nutrition. | (19, 21, 32, 80)  (1, 15, 16, 19, 20, 26, 30, 31, 34, 38, 42, 45-48, 53, 58, 61, 62, 65, 66, 68, 70, 72, 74, 75, 77-84, 86, 88, 89, 91, 96, 101-104, 107, 108) | 48 |
|  |  | Neighborhood Economic Status | Mosquito larval density and breeding site locations | (42, 43, 54) (21, 39) | 5 |
| 2 | Housing and Environmental Conditions | Housing Ownership Status | Homelessness, renting, or owning a home | (47, 50, 60, 69, 103) | 4 |
|  |  | Neighborhood Economic Conditions | Thermal conditions, presence of vegetation, artificial water sources, and their impact on mosquito movement | (2, 11, 71, 90) | 4 |
|  |  | Income-Housing Relationship | Low-income areas are more likely to have immobile water containers, limited access to ventilation systems, private transportation, or mosquito-protective tools. | (15, 17, 22, 37, 50, 58, 59, 69, 71, 79, 85) | 11 |
|  |  |  | Population Density | (1, 2, 29, 48, 51, 55, 58, 59, 63, 64, 69, 78, 79) | 13 |
|  |  | Urban Environmental Design | Urban design, vegetation coverage, presence of abandoned houses, housing composition (rural/urban layout), geographic location, housing density, presence of plastics and coconut shells in the environment, road density | (85) (2, 28, 31, 42, 55, 56, 76, 78, 106) | 9 |
|  |  |  | Illegal housing occupations, crime rates in certain areas, informal settlements, and slums | (104)  (15, 37, 48, 89) | 5 |
|  |  | Housing Quality | Housing structure and mosquito-related housing improvement programs; presence of containers with dead or neglected plants; presence of a building manager; apartment size; housing prices; building age; housing overcrowding | (1, 17, 34, 37, 46, 48, 56, 60, 62, 63, 65, 68, 72-74, 90, 92, 96, 99, 104, 107)  (26, 30, 45, 53, 61, 65, 70, 75, 77, 81, 83, 86, 88, 95, 100-102, 105) | **37** |
|  |  |  | Presence of drinking water storage sources within homes; vegetation in residential yards | (16, 72, 75, 76, 96, 103) | 6 |
|  |  |  | Environmental pollution | (54, 55) | 2 |
|  |  | Type of Housing | Apartment / Residential Complex / Village / Detached House | (25, 35, 48) | 3 |
|  |  | Ecological Factors | Presence of urban heat islands, rainfall, humidity, temperature, geographic location, vegetation cover, altitude above sea level | (3, 29, 31, 40, 51, 64, 66, 78, 107)  (26, 47, 53, 72, 81, 84, 86, 88, 95, 100, 101, 105) | 21 |
| 3 | Education and Health Literacy | Level of Awareness and Health Literacy | General level of awareness regarding disease prevention and control | (74) (15, 19, 31, 43, 58, 59, 73, 80, 85)  (10, 20, 30, 34, 62, 72, 81, 82, 86, 88, 95, 96, 101, 108) | 24 |
|  |  | Educational Attainment | Higher or lower levels of formal education | (76) (1, 15, 21, 41, 42, 48-50, 58, 63, 74, 80, 92, 104, 107)  (20, 27, 38, 46, 47, 53, 62, 75, 77, 82, 83, 105) | 28 |
|  |  | Educational Interventions | Workshops, theater performances, distribution of printed materials on preventive methods, use of visual displays (e.g., statues), education on the use of natural insecticides, training on protective nets and screens, school-based educational programs, initiatives in churches and community centers, technology-based programs, educational groups, and flashcards. | (3, 12, 13, 22, 28, 33, 36, 44, 50, 54, 60, 66, 67, 74, 92, 104) | 16 |
|  |  | Level of Managers’ Awareness | Level of awareness in managing environmental settings that impact health | (11) | 1 |
|  |  | Availability of Resources | Availability of adequate resources and support for implementing educational programs | (56) | 1 |
| 4 | Community Engagement | Educational Approach | Active or passive participation; involvement of community peer educators | (36, 43, 66, 103) (22, 36, 50) | 7 |
|  |  | Existence of Interventions | Community-based nature of interventions and attention to target groups | (22, 42, 44, 74) | 4 |
|  |  | Connection with Health Services | Played a significant role in collaboration with nurses and nursing students for implementing educational programs and monitoring high-risk areas. | (13, 22) | 2 |
| 5 | Urban Infrastructure and Social Support | Support from Relevant Organizations | Support from mosquito control agencies, mosquito control policies and government investment, aid to flood-affected areas, and the financial saving capacity of municipalities | (2, 13, 28, 50, 53, 56, 60-62, 81, 99, 106, 108) | 13 |
|  |  | Intersectoral Collaboration | Multisectoral cooperation among community members, students, health service managers, and schools; public-private partnerships; collaboration across the health, environment, and education sectors | (10, 22, 30, 33, 66, 81, 93, 98, 99) | 9 |
|  |  | Neighborhood Urban Planning Based on Income Level | Expansion of urban gardens | (39) | 1 |
|  |  | Access to Urban Services and Lack of Proper Infrastructure | Water supply, waste management, water accumulation, sewage systems, recycling storage facilities, impervious surfaces such as asphalt and concrete, presence of railways and airports, telephone access, and public transportation networks | (1, 3, 12, 13, 16, 17, 21, 29, 31, 37, 38, 40, 43, 47-49, 51, 54, 55, 58-61, 63, 64, 74, 76, 79, 80, 83-85, 88, 90, 91, 93, 99, 100, 103, 104, 108) | 40 |
| 6 | Access to Health Services | Facilitative Role of Health Centers | Support in implementing educational programs, diagnosing disease cases, identifying high-risk areas, managing disease outbreaks, utilizing health information technologies, and providing prenatal care | (1, 3, 10, 15, 22, 25, 32, 33, 57-59, 61, 64, 73, 76, 81, 85, 89, 91, 93, 94, 98, 100-102) | 25 |
|  |  | Direct and Indirect Treatment Costs | Financial burden leading to reliance on local or traditional treatments | (57) | 1 |
|  |  | Inequity and Limited Access to Basic Health Services | Inadequate medical infrastructure, insufficient insurance coverage, and inefficiencies in health service delivery | (16, 20, 38, 45-47, 59, 61, 62, 65, 69, 88, 89, 93, 95, 96, 100-102, 105) | 20 |
| 7 | Demographic Factors | Gender | Male / Female | (75) | 1 |
|  |  | Race | African descent, non-white populations | (32, 35, 72, 84) | 4 |
|  |  | Age | Elderly individuals | (35, 75) | 2 |
|  |  | Employment | Unemployment rate, home-based occupations, retirees, homemakers, office workers | (25, 49, 83, 95, 105, 107, 108) | 7 |
| 8 | Culture and Beliefs | Cultural Beliefs, Participatory Norms, and Community Empowerment | Cultural practices related to water storage for daily use (due to frequent water shortages) | (21, 88) | 2 |
|  |  |  | Key determinants of the success of educational strategies, individual and social responsibility, fear of disease, and beliefs about curability | (12, 13, 15, 33, 56, 82, 92-94, 98, 108) | 11 |
|  |  |  | Use of traditional treatments; distrust in the public health system | (57) | 1 |
|  |  | Behavioral Patterns | Behavioral tendencies among individuals from different racial backgrounds | (104) | 1 |
|  | Migration and Human Mobility | Migration of low-income populations and travel-related movements | Up to a distance of 8,000 meters | (16, 17, 55, 95)  (35) | 5 |
