# Supplementary material for: Understanding the social determinants of Aedes-borne diseases in Iran: A qualitative exploration of challenges and policy solutions
Source: PLoS Negl Trop Dis. 2025 Dec 22;19(12):e0013850. doi: 10.1371/journal.pntd.0013850 (PMC12753069; doi:10.1371/journal.pntd.0013850)
Supplement: S5 Appendix — (DOCX) [file pntd.0013850.s005.docx]

Appendix 5: PRISMA Flow Diagram – Search, Screening, and Selection of Studies on SDH-Based Interventions Related to Aedes Disease Control

Studies screened & exclusion of criteria applied (n=4361)

Record identified through database searching (n=4944)

Exclusion of duplicates (n=583)

Record excluded (n=4051)

Identification

Screening

Eligibility

Full-text studies assessed for eligibility (n=310)

Eligible studies identified for review (n=71)

Full-text excluded, with reasons (n=239)

Included
