# Supplementary material for: Understanding the social determinants of Aedes-borne diseases in Iran: A qualitative exploration of challenges and policy solutions
Source: PLoS Negl Trop Dis. 2025 Dec 22;19(12):e0013850. doi: 10.1371/journal.pntd.0013850 (PMC12753069; doi:10.1371/journal.pntd.0013850)
Supplement: S6 Appendix — (DOCX) [file pntd.0013850.s006.docx]

**Appendix 6: Interventions Identified from the Literature Categorized by Social and Economic Factors**

| **Category: Social and Financial Protection for Prevention and Treatment Costs** |
| --- |
| - Ensure sustainable financing and political support for implementing vector control and disease prevention programs. - Implement protective measures to reduce treatment costs associated with mosquito-borne diseases. - Integrate disease control efforts with local economic development initiatives. - Address limited access to health, diagnostic, and treatment services in impoverished and high-risk areas; establish 24-hour laboratory services for rapid diagnosis. - Distribute insecticide-treated nets (ITNs). - Targeting Poor Neighborhoods (Poverty): - Areas with inadequate sanitation and higher poverty rates are key breeding grounds for dengue vectors and should be specifically targeted. - Poor health infrastructure and lack of proper waste management create favorable conditions for mosquito proliferation. - Provide low-cost and locally available materials for constructing mosquito traps. |
| **Category: Education** |
| 1. School-Based Educational Programs  - Implement awareness programs for students and their families on disease prevention. - Use iDEAL educational modules in Malaysia to improve knowledge, attitudes, and preventive behaviors. - Educate children and their families on mosquito-borne disease risks. - Develop educational programs for schools and communities to raise awareness and promote behavior change.  1. Public Awareness Campaigns  - Distribute visual educational materials for low-literacy populations. - Use social media and digital tools for public education. - Provide visual aids and educational content to improve public awareness, particularly in low-literacy communities. - Design multilingual educational initiatives targeting specific population groups. - Strengthen communication channels for timely public engagement during outbreaks.  1. Training of Health Workers  - Enhance technical knowledge among health workers for vector control and surveillance program implementation.  1. Community-Based Participation  - Encourage and train local communities to identify and eliminate mosquito breeding sites. - Promote self-care practices and behavior change for sustainable prevention. - Establish Community Working Groups (CWGs) composed of local leaders, health workers, and residents. |
| **Category: Unemployment and Job Security (Occupational Living Conditions)** |
| 1. Targeting High-Risk Occupations  - Provide preventive and protective measures for workers in contaminated environments. - Deliver educational programs for municipal workers (e.g., in Puducherry, India). - Reduce workers’ exposure to disease vectors by improving workplace environments. - Conduct regular inspections and monitoring of occupational settings to eliminate mosquito breeding sites. |
| **Category: Housing, Infrastructure, and Environment** |
| 1. Infrastructure Improvement  - Urban design modifications to prevent water accumulation and reduce mosquito habitats - Maintenance and renovation of storm drains to eliminate breeding sites - Improving sanitary conditions in impoverished areas to reduce stagnant water sources and mosquito habitats - Use of low-cost technologies to reduce environmental pollution  1. Environmental Management  - Reducing mosquito breeding sites through waste management and infrastructure upgrades - Legal measures for environmental sanitation, including monetary fines for non-compliance with dengue prevention laws - Enforcing regulations for managing mosquito habitats and reducing reproduction sources - Urban green space management - Monitoring and maintenance of public infrastructure, such as storm drains, to prevent water accumulation - Prohibition of water containers in cemeteries to prevent mosquito breeding - Routine inspections of homes, including commercial properties, vacant lots, and strategic points such as cemeteries and waste sites - Regular monitoring of storm drains as potential breeding and resting sites - Research on addressing climate change impacts, such as increased rainfall and temperature, affecting mosquito habitats - Covering water storage containers with mesh or insecticide-treated lids - Removal of unused small containers - Concrete lining of storm drains to prevent water stagnation - Routine spraying and mosquito trapping  1. Improving sewage systems and ensuring proper water flow to avoid stagnation  - Housing Improvement - Promoting the use of window screens, air conditioning, and proper household ventilation - Modifying housing designs, including gutters and water outlets - Upgrading public housing and eliminating mosquito breeding sites (e.g., using screened windows or air conditioning) - Altering building designs to reduce mosquito reproduction potential (e.g., covering water containers) |
| **Category: Multisectoral, Regional, and International Efforts by joint initiatives** |
| - Global and regional collaborations, such as the World Mosquito Program, to implement and sustain vector control efforts - Coordination with city councils, private sector actors, and academic institutions - Integration of genomic data with clinical and epidemiological datasets to enhance surveillance and inform intervention planning - Development and alignment of national and regional strategic vector control programs - Strengthening vector surveillance systems integrated with health information systems - Multisectoral partnerships involving education, water, sanitation, and public health sectors - Inclusion of stakeholders—such as local communities and policymakers—in the design and implementation of programs - Joint monitoring of mosquito density changes associated with temperature and rainfall - Cross-border initiatives between countries, such as Mexico and the United States, to prevent reintroduction - Requiring travelers to provide proof of vaccination to prevent disease spread - Identification and analysis of transmission patterns - Border, port, and airport surveillance: regular monitoring of mosquito populations, particularly Aedes species, for detection and control - Focus on points of entry: inspection and regulation of incoming cargo and travelers - Regional coordination: sharing data and surveillance strategies with neighboring countries to synchronize dengue prevention and control actions |
