# Supplementary material for: Understanding the social determinants of Aedes-borne diseases in Iran: A qualitative exploration of challenges and policy solutions
Source: PLoS Negl Trop Dis. 2025 Dec 22;19(12):e0013850. doi: 10.1371/journal.pntd.0013850 (PMC12753069; doi:10.1371/journal.pntd.0013850)
Supplement: S7 Appendix — (DOCX) [file pntd.0013850.s007.docx]

**Appendix 7: Profile of Participants in the Qualitative Component of the Study**

| **Duration (Minutes)** | **Work Experience (Years)** | **Gender** | **Organizational Position** | **Education** | **Organizational Affiliation** |
| --- | --- | --- | --- | --- | --- |
| 100 (as part of a focus group discussion) | 36 | Male | Head of Vector-Borne Diseases Department | Doctor of Veterinary Medicine (DVM) and Master of Public Health (MPH) in Epidemiology | Ministry of Health and Medical Education |
|  | 21 | Male | Senior Expert on Vector-Borne Diseases | Medical Doctor (MD) | Ministry of Health and Medical Education |
|  | 26 | Female | Dengue Fever Human Surveillance Officer | Medical Doctor (MD) | Ministry of Health and Medical Education |
|  | 15 | Female | Communicable Diseases Expert | Bachelor’s Degree | Ministry of Health and Medical Education |
|  | 8 | Female | Communicable Diseases Expert | Bachelor’s Degree | Ministry of Health and Medical Education |
|  | 29 | Female | Communicable Diseases Expert | Master’s Degree and MPH | Ministry of Health and Medical Education |
|  | 22 | Female | Communicable Diseases Expert | Master’s Degree | Ministry of Health and Medical Education |
| 18 | 29 | Male | Faculty Member | PhD in Health Policy and Economics | Health Equity Research Center |
| 45 | 16 | Female | Expert, Vector Control Department | PhD in Entomology | Ministry of Health and Medical Education |
| 56 | 21 | Male | Head, Department of Vector and Biological Agents Control – Environmental and Occupational Health Center | PhD Candidate | Ministry of Health and Medical Education |
| 43 | 30 | Male | Faculty Member | Specialist in Infectious and Tropical Disea | Iran University of Medical Sciences |
| 35 | 10 | Male | Faculty Member – Head of the Department of Vector Biology and Control | PhD in Medical Entomology | Hormozgan University of Medical Sciences |
| 35 | 30 | Male | Faculty Member – Vice Dean for Education, School of Public Health | PhD in Medical Entomology and Vector Control | Hormozgan University of Medical Sciences |
| 40 | 15 | Male | Faculty Member – Director of the Social Determinants of Health Research Center | PhD in Biostatistics | Hormozgan University of Medical Sciences |
| 32 | 15 | Male | enior Research Expert in Health System Research (HSR), Deputy for Public Health | PhD in Health Education and Health Promotion | Hormozgan University of Medical Sciences |
| 30 | 9 | Male | Faculty Membe | PhD | Guilan University of Medical Sciences |
| 37 | 2 | Female | Expert in Vector Biology and Disease Control | Bachelor’s Degree | Bushehr University of Medical Sciences |
| 30 | 22 | Male | Head of Communicable Disease Prevention and Control Department | Medical Doctor (MD) | Ministry of Health and Medical Education |
| 35 | 15 | Male | Expert in Zoonotic Disease Control | Bachelor’s Degree | Chabahar University of Medical Sciences |
| 40 | 5 | Female | Expert in Health Education and Promotion | Bachelor’s Degree | Ministry of Health and Medical Education |
|  | 1 | Male | Director, Department of Health Education and Promotion | Specialist in Infectious Diseases | Ministry of Health and Medical Education |
