# Supplementary material for: Understanding the social determinants of Aedes-borne diseases in Iran: A qualitative exploration of challenges and policy solutions
Source: PLoS Negl Trop Dis. 2025 Dec 22;19(12):e0013850. doi: 10.1371/journal.pntd.0013850 (PMC12753069; doi:10.1371/journal.pntd.0013850)
Supplement: S8 Appendix — (DOCX) [file pntd.0013850.s008.docx]

**Appendix 8: Policy Strategies Targeting SDHs for the Prevention and Control of Aedes-Borne Diseases in Iran**

| **Education and awareness strategies** | | |
| --- | --- | --- |
| **Subtopic** | **Code** | **Proposed intervention** |
| General education | Educational campaigns | Design and implement educational campaigns in schools, mosques and media to prevent mosquito bites and eliminate larval habitats. |
| Specialized training for health staff | Specialized courses | Hold specialized courses to strengthen the skills of healthcare personnel in identifying and controlling the disease. |
| Use of local leaders’ influence | Leader participation | Enlist participation of religious and local leaders to change people’s behaviour and raise awareness. |
| Educational tools | Local media | Use local media to provide information and prevention messages. |
| Targeting specific groups | Education for vulnerable groups | Provide targeted educational programmes for specific groups such as housewives and children. |
| **Environmental improvement strategies** | | |
| Management of stagnant water | Draining stagnant water | Remove accumulated water in containers, beneath air conditioners and in tanks. |
| Improving drainage | Drainage development and maintenance | Develop and maintain drainage systems for streets and alleys. |
| Waste management | Reducing waste accumulation | Create systems for sanitary collection and disposal of rubbish to reduce waste accumulation. |
| Public clean‑up | Organising clean‑up campaigns | Organise environmental clean‑up campaigns in urban and rural areas. |
| Sewer system reform | Expanding sewer networks | Improve and develop urban and rural sewage systems to prevent open sewage discharge. |
| Management of open sewage | Replacing sanitary methods | Replace open sewage disposal methods in disadvantaged areas with sanitary alternatives. |
| **Health infrastructure strategies** | | |
| Access to health services | Expansion of health centres | Increase the number of health centres in remote and high‑risk areas. |
| Human‑resource distribution | Balanced distribution of doctors and specialists | Ensure a balanced distribution of doctors and specialist staff in deprived areas. |
| Strengthening disease surveillance | Disease surveillance systems | Create active disease surveillance systems to quickly identify suspected cases. |
| Equipment and facilities | Provision of medical equipment | Upgrade laboratory and medical equipment in treatment centres in high‑risk areas. |
| Referral and transport systems | Improving referral systems | Establish structures for the rapid referral of patients from rural areas to designated treatment centres. |
| **Economic factors strategies** | | |
| Financial support for underserved communities | Health equipment subsidies | Provide subsidies for purchasing health equipment such as appropriate containers for water storage. |
| Reducing treatment costs | Free healthcare services | Offer free or subsidised healthcare services for low‑income patients. |
| Local job creation | Jobs related to environmental improvement | Create job opportunities for environmental clean‑up, waste management and monitoring of environmental improvements. |
| **Social factors strategies** | | |
| Social participation | Forming volunteer groups | Create community volunteer groups to eliminate larval habitats and monitor the environment. |
| Health‑literacy promotion | Preventive education | Provide public education on the role of individuals in preventing mosquito‑borne diseases. |
| Strengthening social networks | Local communications | Use local social networks to quickly convey information related to disease control. |
| Role of local leaders | Gaining leaders’ support | Utilise local and religious leaders to encourage people to implement preventive interventions. |
| Interaction between institutions and the public | Increasing intersectoral cooperation | Create shared platforms between government organisations and communities for disease control and environmental interventions. |
| **Cultural factors strategies** | | |
| Programme localisation | Adapting to local culture | Design interventions tailored to the customs and cultural beliefs of different regions. |
| Use of local media | Cultural education | Use local media to provide information related to disease control. |
| Cultural events | Local events | Hold cultural events focused on raising awareness about prevention and control of the disease. |
| Strengthening social trust | Role of religious and social leaders | Leverage the influence of religious and social leaders to increase public trust in health interventions. |
| Respect for cultural diversity | Multicultural programmes | Design programmes that consider cultural diversity and respect cultural sensitivities. |
| **Strategies related to mosquito species characteristics** | | |
| Species differences | Identifying *Aedes albopictus* and *Aedes aegypti* | Design interventions tailored to each species’ biological behaviour, such as the Aedes aegypti’s preference for artificial habitats. |
| Habitat patterns | Managing natural and artificial habitats | Clean flower pots, remove stagnant water containers and eliminate natural habitats such as moist plants. |
| Biological behaviour | Reducing human–mosquito contact | Use methods such as installing screens, using repellents and bed nets to reduce direct contact. |
| Reproductive cycle | Control of egg‑laying | Eliminate small stagnant water sources that could serve as mosquito breeding sites. |
| Geographical distribution | Regional planning | Develop programmes tailored to the distribution of mosquito species in northern and southern parts of the country. |
