# Supplementary material for: Understanding the social determinants of Aedes-borne diseases in Iran: A qualitative exploration of challenges and policy solutions
Source: PLoS Negl Trop Dis. 2025 Dec 22;19(12):e0013850. doi: 10.1371/journal.pntd.0013850 (PMC12753069; doi:10.1371/journal.pntd.0013850)
Supplement: S9 Appendix — (DOCX) [file pntd.0013850.s009.docx]

**Appendix 9: Prioritization of Identified Interventions Based on Defined Criteria**

| Row | **Intervention Title** | **Average Score for Each Criterion** | | | | **Simple Mean** | **Weighted Mean** | **Priority (Weighted)** | **Entropy Score** | **Priority (Entropy)** |
| --- | --- | --- | --- | --- | --- | --- | --- | --- | --- | --- |
|  |  | **Effectiveness** | **Operational Feasibility** | **Social Acceptability** | **Political Acceptability** |  |  |  |  |  |
| 1 | Specialized training for healthcare workers in disease detection and control | 7.625 | 8.375 | 8.125 | 8.125 | 8.063 | 8.050 | 1 | 8.190 | 1 |
| 2 | Integrating Aedes disease prevention education into national curriculum (school to university) | 8.500 | 7.125 | 7.875 | 6.500 | 7.500 | 7.563 | 2 | 7.266 | 7 |
| 3 | Use of local media and native languages for effective health communication | 7.500 | 7.625 | 7.750 | 7.125 | 7.500 | 7.513 | 3 | 7.517 | 2 |
| 4 | Use of social media and digital tools for rapid health communication | 8.250 | 7.125 | 8.125 | 5.750 | 7.313 | 7.388 | 4 | 7.106 | 9 |
| 5 | Localization of prevention programs adapted to local traditions | 8.625 | 6.875 | 7.000 | 6.625 | 7.281 | 7.375 | 5 | 7.050 | 11 |
| 6 | Culturally adapted disease prevention and control programs | 7.875 | 6.625 | 7.500 | 7.625 | 7.406 | 7.375 | 6 | 7.135 | 8 |
| 7 | Broad dissemination using local media and native languages | 7.500 | 7.375 | 7.375 | 7.125 | 7.344 | 7.363 | 7 | 7.334 | 5 |
| 8 | Engaging local/religious leaders to encourage preventive action | 7.375 | 7.375 | 7.000 | 7.625 | 7.344 | 7.350 | 8 | 7.374 | 3 |
| 9 | Active surveillance and detection systems for rapid case identification | 8.250 | 6.875 | 7.625 | 6.375 | 7.281 | 7.338 | 9 | 7.044 | 12 |
| 10 | Use of local social networks for awareness and disease control info | 7.125 | 7.750 | 7.625 | 6.375 | 7.219 | 7.263 | 10 | 7.347 | 4 |
| 11 | Comprehensive stagnant water management and drainage improvement | 9.125 | 5.500 | 8.625 | 5.750 | 7.250 | 7.263 | 11 | 6.474 | 28 |
| 12 | Strengthening solid waste management in high-risk areas | 9.000 | 5.750 | 8.250 | 5.875 | 7.219 | 7.250 | 12 | 6.555 | 25 |
| 13 | Improving referral systems from rural to equipped health centers | 7.750 | 6.500 | 8.000 | 6.750 | 7.250 | 7.225 | 13 | 6.937 | 14 |
| 14 | Intersectoral collaboration between environment, health, and other sectors | 8.750 | 6.250 | 7.875 | 5.750 | 7.156 | 7.225 | 14 | 6.690 | 22 |
| 15 | Targeted education programs for high-risk/vulnerable groups | 7.375 | 7.000 | 6.875 | 7.250 | 7.125 | 7.138 | 15 | 7.082 | 10 |
| 16 | Health campaigns in schools, mosques, and media | 6.375 | 7.625 | 6.875 | 7.500 | 7.094 | 7.075 | 16 | 7.331 | 6 |
| 17 | Cross-sector collaboration between government and community | 9.000 | 6.125 | 7.500 | 5.125 | 6.938 | 7.063 | 17 | 6.460 | 29 |
| 18 | Public clean-up campaigns and waste disposal initiatives | 8.125 | 5.500 | 7.875 | 6.875 | 7.094 | 7.038 | 18 | 6.490 | 27 |
| 19 | Community-based media networks for public health messaging | 8.000 | 6.375 | 7.250 | 6.375 | 7.000 | 7.038 | 19 | 6.706 | 21 |
| 20 | Health literacy promotion via targeted education programs | 7.625 | 6.125 | 7.000 | 7.500 | 7.063 | 7.025 | 20 | 6.749 | 20 |
| 21 | Role of religious/social leaders in public communication | 7.250 | 6.875 | 6.625 | 7.000 | 6.938 | 6.963 | 21 | 6.910 | 15 |
| 22 | Promoting health messages via local and religious leaders | 6.625 | 7.000 | 6.375 | 7.625 | 6.906 | 6.888 | 22 | 6.999 | 13 |
| 23 | Securing support from local/religious leaders for community action | 6.750 | 6.750 | 6.250 | 7.625 | 6.844 | 6.825 | 23 | 6.870 | 16 |
| 24 | Advocacy by leaders for community prevention efforts | 7.125 | 6.750 | 6.250 | 7.000 | 6.781 | 6.813 | 24 | 6.775 | 19 |
| 25 | Cultural events to promote disease prevention | 6.875 | 6.875 | 6.625 | 6.750 | 6.781 | 6.800 | 25 | 6.809 | 17 |
| 26 | Free/subsidized healthcare for low-income patients | 7.750 | 5.375 | 8.750 | 5.375 | 6.813 | 6.763 | 26 | 6.180 | 31 |
| 27 | Targeted education for vulnerable groups (e.g., women, children) | 6.875 | 6.375 | 6.750 | 6.875 | 6.719 | 6.700 | 27 | 6.605 | 23 |
| 28 | Expanding health centers in remote/high-risk areas | 7.000 | 6.000 | 7.875 | 6.125 | 6.750 | 6.700 | 28 | 5.751 | 35 |
| 29 | Leader engagement for social acceptance and behavior change | 6.375 | 6.750 | 6.500 | 7.250 | 6.719 | 6.688 | 29 | 6.778 | 18 |
| 30 | Subsidies for hygienic supplies (e.g., covered containers, nets) | 8.500 | 4.250 | 8.750 | 5.000 | 6.625 | 6.575 | 30 | 5.623 | 40 |
| 31 | Job creation through waste management/clean-up projects | 8.375 | 4.875 | 7.500 | 5.500 | 6.563 | 6.575 | 31 | 5.842 | 33 |
| 32 | Drainage improvement and water container management | 8.750 | 3.875 | 8.000 | 5.875 | 6.625 | 6.563 | 32 | 5.546 | 41 |
| 33 | Local media for public disease control messages | 6.500 | 6.500 | 6.250 | 6.750 | 6.500 | 6.500 | 33 | 6.518 | 26 |
| 34 | Dual health–economic development via environmental jobs | 8.250 | 4.750 | 7.750 | 5.250 | 6.500 | 6.500 | 34 | 5.746 | 36 |
| 35 | Cultural events to raise Aedes awareness | 6.250 | 6.750 | 6.500 | 6.500 | 6.500 | 6.500 | 35 | 6.595 | 24 |
| 36 | Equipping health centers with advanced diagnostic tools | 7.125 | 5.375 | 7.500 | 6.000 | 6.500 | 6.450 | 36 | 6.053 | 32 |
| 37 | Active case detection and surveillance | 8.000 | 4.625 | 6.875 | 6.250 | 6.438 | 6.413 | 37 | 5.743 | 37 |
| 38 | Stagnant water control under coolers and tanks | 8.625 | 4.875 | 5.750 | 5.750 | 6.250 | 6.350 | 38 | 5.660 | 38 |
| 39 | Improving access to care in remote/high-risk areas | 7.125 | 4.750 | 8.125 | 5.625 | 6.406 | 6.313 | 39 | 6.437 | 30 |
| 40 | Volunteer groups for larval habitat removal and monitoring | 8.250 | 4.500 | 5.375 | 7.000 | 6.281 | 6.300 | 40 | 5.649 | 39 |
| 41 | Community participation in disease/environmental control | 7.750 | 5.000 | 6.000 | 6.375 | 6.281 | 6.300 | 41 | 5.795 | 34 |
| 42 | Financial incentives for households/businesses to improve environment | 8.125 | 4.250 | 7.625 | 5.000 | 6.250 | 6.238 | 42 | 5.405 | 43 |
| 43 | Subsidies for protective nets and safe water containers | 7.500 | 4.250 | 8.625 | 4.750 | 6.281 | 6.200 | 43 | 5.427 | 42 |
| 44 | Sewer development and sanitation solutions in deprived areas | 8.000 | 3.500 | 7.750 | 5.250 | 6.125 | 6.050 | 44 | 5.089 | 45 |
| 45 | Equipping clinics with diagnostic tools for early case identification | 7.000 | 4.125 | 8.125 | 5.125 | 6.094 | 5.988 | 45 | 5.311 | 44 |
