# Supplementary material for: Understanding the social determinants of Aedes-borne diseases in Iran: A qualitative exploration of challenges and policy solutions
Source: PLoS Negl Trop Dis. 2025 Dec 22;19(12):e0013850. doi: 10.1371/journal.pntd.0013850 (PMC12753069; doi:10.1371/journal.pntd.0013850)
Supplement: S2 File — (DOCX) [file pntd.0013850.s011.docx]

**S2 File:**

**Full transcripts of qualitative interviews with key informants, including provincial and national stakeholders, conducted as part of the study on social determinants influencing Aedes-borne diseases in Iran.**

**1^st^ Interviewee (Female)**

**Interviewer:** Very well, thank you very much, Doctor. I can hear your voice. Yes, yes, thank you, thank you very much. First, I would like to thank you for the time you are giving us and the points you are going to explain to us, and I am really grateful. You are helping us a lot, and I appreciate it, Doctor. Thank you, stay healthy. The study we want to conduct aims to identify the social determinants that affect the prevention, control, and spread of Aedes-borne diseases. As you are aware, these factors that affect, for example, the transmission of mosquitoes, we have categorized based on our previous studies into three groups: environmental factors, then social and economic factors, and the next category relates to health and medical infrastructures. After that, we also want to discuss the strategies and interventions that can be practical in order to reduce the impact of these factors and to help in disease control, and we would like to review them together and be able to benefit from your valuable insights. Well, if you allow, we will start our interview with environmental factors. I request that you please tell us what environmental factors exist that have played a role in the spread of Aedes mosquitoes in the country?

**Interviewee:** You see, in the world, in fact, there is a process, an event, happening which is called climate change. Because of this event that is taking place and the earth is warming, some parts of the world that until today were not suitable for the growth of certain insects are becoming suitable. At the same time, another event is happening: due to exchanges—in fact, the exchange of goods and the many travels that exist around the world—the mosquito can more easily expand its territory. This means that at present, as you know, the world is like a global village, and in this village, very quickly, in less than 24 hours, you can reach another continent. Along this route, mosquitoes can be transported through goods—goods such as tires, used second-hand tires, which in global trade are large, and also through cars, airplanes, and a plant called lucky bamboo. These are the main ways mosquitoes can be transported, and mosquitoes can be transferred in different forms to various parts of the world where they did not exist before. For example, in the form of eggs or larvae, they can be carried in lucky bamboo plants, in tires, or in plants like coconuts or similar items that have some moisture, up to adult mosquitoes that can be transported by airplane, by cars, ships, and such goods. So, there are two factors: one is climatic conditions, climate change, which has led to more favorable weather for the growth of mosquitoes in our country, and the second is trade of goods and the movement of people in various ways.

**Interviewer:** Uh-hum, very well, thank you. Now, in your opinion, Doctor, in this environment that exists, does housing, for example, depending on whether people have different types of housing, have an effect or not? In what way? And what about water?

**Interviewee:** The question you asked was more about the environmental conditions for mosquito growth and spread. Yes, when the mosquito enters a place, a region, for it to be able to grow and establish itself, multiple factors play a role. The most important of these factors goes back to the larval habitat. The larval habitat means any place where water collects—water with specific characteristics is important. Usually, it is said to be clean or fresh water, and usually in small amounts. But it can also be in larger volumes. So, when the mosquito enters a place and there is a suitable larval habitat for it, this can be anything from a small container under a flowerpot, to a container under an air conditioner, to surface water left exposed, and all sorts of containers in which people, for whatever reason, are forced or prefer to collect water.

Now, when you use the word housing, the newer the housing, the lower the potential risk. But this does not mean that older houses cannot also be of importance. Part of this issue relates to people’s culture and their health literacy. If we set aside economic conditions that force people to collect water in certain ways, after that, it goes back to health literacy. That means whether, after the trainings and warnings given to the people of a region, they understand what larval habitats are—those suitable for mosquito growth and development—and whether they comply and apply what they have learned.

For example, someone may have a new house, but in that house, for any reason, water pipes or condensate from air conditioners may be left unattended, dripping into containers, or the plumbing may have been extended to a certain point and then left unfinished, where water leaks and accumulates. On the other hand, in new buildings, especially in humid areas like the north and south of our country near the sea, the water table is very high, which can lead to elevator shafts or those spaces designed for elevators, in both inhabited and half-finished houses, filling up with water.

So, part of this goes back to the health literacy of the builder, and the second part goes back to the oversight of the engineering and construction organizations, which need to be trained. The engineering system needs to know what points must be checked and observed during construction or at the time of a building’s completion, when they come for inspection and service delivery.

So, first comes health literacy. After that, we can talk about whether buildings are new or not, but this should not be overemphasized. Finally, apart from all this, the climatic conditions in a region matter. In humid areas, in addition to the high groundwater level, given the species of Aedes mosquito—Aedes albopictus in fact grows more in forested areas—even something as small as dew can be enough. Dew on plants, flowers, and tree surfaces can become a larval habitat. So here, the type of housing is not important at all. It depends more on the weather and climate of the area, because this mosquito prefers to grow in forested regions. Forested areas near human settlements are important for us. Other remote forest areas are not. These forested areas, due to the presence of humidity and all kinds of suitable environments where rainwater can accumulate—or even apart from rainwater, dew on flowers—can serve as necessary and suitable larval habitats.

**Interviewer:** Thank you very much, Doctor. I appreciate it. Thank you. Doctor, my question in this part is whether, since we said we are studying four provinces—Gilan, Bushehr, Hormozgan, and Chabahar—do the environmental factors that play a role in the spread of Aedes mosquitoes differ between these provinces? Are there factors that, for example, play a role in Gilan but have less impact in Chabahar?

**Interviewee:** Yes. You see, the conditions of water storage in the north and the south of the country are completely different. In the south, due to the lack of stable drinking water—that is, a reliable piped water supply—there are frequent water cutoffs for many hours during the day. People are forced to store water in tanks or water containers. Families who are economically well-off and have better health literacy use fully sanitary, covered water containers. But families who, for various reasons, face health poverty, low health literacy, and economic and social poverty, store water in any container. This is a problem in the south of the country. In the north, we have fewer water cutoffs that force people to store water.

Yes, besides this, the mosquito species in the north is completely different from that in the south. As I mentioned earlier, in the north, especially in forested and green areas, mosquito growth and reproduction are much higher than those in the south. The major difference is that the mosquito in the south survives and persists mainly in the form of eggs. It is specifically adapted to drought-prone areas. The mosquito has synchronized itself with the climatic conditions there. Since there is not much rainfall in those areas and the mosquito needs water, it can usually survive for a long time in the form of eggs. Whereas in the north, the mosquito survives as an adult—the flying mosquito you see. It can overwinter and survive the cold season in this form, having adapted to remain as an adult. It does not depend as much on artificial water containers created by humans. The reason is that in the north there are frequent rains.

Perhaps the only similarity in larval habitats between north and south is in cultural practices of the people. For example, keeping flowers in glass vases, overwatering potted plants that causes water to collect under flowerpots, or air conditioners in both climates. In both places, containers are placed under air conditioners where condensate collects. Sometimes this water is used for irrigation, sometimes for livestock, and sometimes containers are put there to prevent water from making the ground behind their houses damp for a long time. There are multiple reasons, but the result is the same: a suitable larval habitat.

As I mentioned, in the north we have even observed mosquito growth in flower dew during our studies, while this has been less common in the south so far. But in the south, we also have a saying: “two teaspoons of water are enough for mosquitoes to breed.” This applies to both climates. But the main difference is the availability of stable drinking water—scarce in the south, more available in the north.

Another difference is that in the north there is relatively more urban development. Although mosquitoes can thrive in nearby forests close to human settlements, generally we see less sewage and waste in the north. People there are usually more well-off. Culturally, people in the north seem more familiar with health literacy, with other aspects of health, cultural and social literacy, and because of sewage piping, surface wastewater is less likely to flow into the streets.

In the south, however, in the provinces where mosquitoes are now present—almost in all cities except the very large ones, and even in much of the larger cities, like Bandar Abbas—the sewage system is still not developed. Wastewater from washing, bathing, or toilets is not properly directed and often ends up in the streets. This is a more prominent problem in the south than in the north.

The differences between the two regions, apart from other smaller ones, mainly come down to this: both regions have half-finished buildings, elevator shafts, and spaces where water can accumulate in incomplete buildings. But the most significant difference is the lack of stable drinking water in the south.

**Interviewer:** Thank you very much, Doctor. By “south,” you meant the provinces of Bushehr, Hormozgan, and Chabahar, right?

**Interviewee:** Yes, in the provinces of Bushehr, Hormozgan, and Sistan and Baluchestan we are actually dealing with *Aedes aegypti* mosquitoes, which have adapted to drier, less rainy regions. In contrast, *Aedes albopictus* mosquitoes are specific to rainy, forested areas, similar to the north of our country. At present, they are found in Mazandaran and Gilan, and also as isolated cases in Zanjan, Qazvin, Ardabil, and East Azerbaijan. But the main centers where they have currently grown and reproduced are Gilan and Mazandaran.

**Interviewer:** Thank you very much. Well, regarding the social and economic factors, Doctor, in your opinion, what factors influence the spread of Aedes mosquitoes?

**Interviewee:** You see, economic factors can actually have two dimensions. One dimension relates to the spread of mosquitoes. I cannot say that a better economy alone will result in fewer mosquitoes, but a better economy combined with health literacy can reduce the necessary risks. As a matter of fact, I conducted an assessment in Chabahar with colleagues. In areas where people’s living conditions are better, there are fewer active larval habitats. An active larval habitat is different from a potential larval habitat. A potential larval habitat means any place where water can accumulate—from tanks to elevator shafts, to sewage and surface water, to ponds and pools. It becomes active when larvae are present in it. So, according to the study conducted, wherever the economy is better, there are usually fewer active larval habitats. But this does not always happen.

You should know that sometimes mosquitoes prefer hidden larval habitats, which are called larval habitats hidden from human sight. These include flower containers, glass flower vases, water under air conditioners, dripping surface water in hidden spots like parking areas, patios, and backyards. However, in lower-income areas in the south of the country, people—because of the lack of drinking water and limited economic means—use all kinds of containers. As a result, they have more potential larval habitats. And since the number of potential larval habitats is higher, the number of active larval habitats can also increase proportionally—not equally, but relatively higher.

On the other hand, in underprivileged or poorer areas, we usually see higher population density. In fact, more people live there. In that same assessment I did in Chabahar with my colleagues, when you look at disease incidence, specifically the incidence of dengue fever, you realize that areas with higher population density have more patients, which is completely natural. In fact, the incidence can go up to a very high number, as high as 900. Whereas in the Chabahar Free Zone, where we have fewer larval habitats and fewer households, and thus a smaller population, the number of patients is also lower accordingly. This has been completely proven: worldwide, densely populated areas have more patients, because more people are exposed to mosquito bites.

So that was the part related to mosquitoes. Now let us move to the second part, where we assess the relationship of the economy with patients and the number of patients. The reality is that more affluent families can visit doctors more easily. Economically, when there is no equal access to medical and healthcare facilities, the poor generally visit doctors less often or later. So what happens is that in underprivileged areas, since people visit doctors later after the onset of symptoms, the disease can spread more. This means that a patient, from two days before the onset of symptoms until about one week to ten days after the onset of symptoms, can transmit the virus to other people—that is, to healthy mosquitoes that then become infected and transmit the disease to others. Transmission occurs through mosquitoes, and late visits make it worse. In contrast, more affluent families visit doctors earlier, so we can interrupt the transmission cycle of the disease sooner, thanks to the control interventions we carry out after the emergence of each positive mosquito case.

You had also asked about social factors, correct? Yes, Interviewer, yes. Well, regarding social factors, it seems that socially advantaged individuals are more likely to cooperate. But this is not always the case. Very often, socially advantaged families do not cooperate at all with the health system. They usually only visit the health system when they want to vaccinate their children. So, in a way, our access to affluent families for promoting awareness and knowledge about mosquito control and disease prevention is weaker than with underprivileged families.

On the other hand, underprivileged families may actually be more receptive to the health system because they visit more often, have better interaction, and are more in need of healthcare services. So, in a way, underprivileged families usually have a better relationship with health workers, being more receptive and more sensitive to health advice. In fact, in this very study, I conducted a rapid assessment of a number of patients in Chabahar. I came to the conclusion that income, education, and occupation do not have a direct or significant relationship with people’s level of awareness and performance.

Although the assumption is that people with better economic or social status should have better knowledge and performance when facing a health problem, in the study population—which was small, about 800 patients—I randomly assessed ten percent of them with a questionnaire. Ultimately, I concluded that there is no connection between income, occupation, and education. But this requires a broader investigation to be certain and to conclude that health literacy can vary in both groups, both affluent and poor. Health literacy does not necessarily result from income, education, or occupation.

**Interviewer:** Thank you very much, Doctor. I appreciate it. I think you have referred to all the factors. Is that correct? Is there anything else we should mention?

**Interviewee:** I don’t think so. The only thing I might want to add is that we should understand that because the mosquito actually has a wide flight radius, it is often not possible to draw a line between poor and affluent areas. Why do I say this? Because it is active during the day, and people, regardless of where they live or which part of the city they are in, and regardless of the level of risk factors, move around during the day. This means poor and affluent individuals can come into contact in the same area, for example, at the market they visit or at recreational centers. This is not directly related to income level. Therefore, we must also consider that, due to the nature of life and living environments, mobility and exposure can play a role, and the influence of socioeconomic status on disease may be diminished.

**Interviewer:** Thank you, Doctor. I appreciate your kindness. I have benefited from your words. Regarding the next factor, which I think plays a role in the spread and transmission of Aedes mosquitoes, it would be the healthcare infrastructure, right? For example, whether there is a health center, a hospital, or access to healthcare services? Yes, please explain this to us.

**Interviewee:** You see, among the affluent or technical classes, they visit the health system less, but they tend to use private healthcare services. Now, this depends on the disease surveillance system in each region or country. If the private healthcare system—including clinics and hospitals—has its reporting system connected to the government reporting system, we can benefit from the fact that affluent individuals go to private healthcare centers, allowing us to diagnose cases in a timely manner and prevent the spread of the disease. This is important because dengue is a contagious disease that can be transmitted to others via mosquitoes.

On the other hand, if access to healthcare services is insufficient, this means that coverage in poorer neighborhoods is lacking in terms of access, sufficient personnel, or diagnostic services, and timely diagnosis or treatment in hospitals is inadequate. This directly affects the number of patients, which can increase. How does this happen? If the number of rapid diagnostic kits or our laboratories does not provide timely results, we face delays in diagnosing the disease and preventing its spread. Additionally, if patients encounter long waiting lines at healthcare centers, this also contributes to disease spread.

Furthermore, if individuals do not have health insurance—whether it’s the national health insurance, social security, or other public insurance such as for armed forces personnel or certain banks—they are less likely to seek care when they get sick. Dengue is an urban disease, and in cities where we encounter it, such as Chabahar, Bandar Lengeh, or some other southern cities, some of the population is considered poor. Lack of access to diagnostic and treatment facilities can lead to more severe cases, or individuals may not seek care at all and self-treat.

The reality is that dengue fever can often resolve on its own, except in individuals with underlying health conditions, who are considered high-risk. Otherwise, dengue can be managed with home treatments, such as taking painkillers and resting. Even for healthy individuals, this is the recommended approach medically.

So, what harm occurs if people lack access to diagnostic and treatment services and self-treat? The disease transmission cycle continues and can rapidly cause the disease to spread. The harm from self-treatment and not seeking care is exactly this.

**Interviewer:** Thank you, Doctor. I appreciate your kindness. In this part, my question is whether, considering the cities in the various provinces we are studying, the healthcare infrastructure and access to these healthcare facilities still affect the spread of mosquitoes by province? By healthcare services? I mean, does it have an impact? For example, by province—so that city by city, Chabahar versus Bushehr, or Hormozgan versus Gilan, due to access to healthcare services, there could be differences?

**Interviewee:** You see, if their access is equal—that is, if all our comprehensive health centers are active in every province—then logically there should be no difference. Of course, there is one difference, which I will explain, but initially, I should tell you that access to diagnostic and treatment services in the south and north of the country is very, very different. In the south, for various reasons—hardship, climate, distance from the central regions—many diagnostic and treatment facilities, as well as specialists and physicians, are reluctant to work in these areas, and we usually have a shortage of human resources. In northern Iran, generally, we usually have less shortage of human resources or comprehensive health centers compared to the south.

For this reason, since people in the north have easier access to the healthcare system, logically we should have fewer problems there. As the COVID-19 experience showed us, in the north we had a higher number of reported patients compared to the south, and part of this was due to lack of access to sufficient diagnostic and treatment facilities in the south, as well as cultural factors and the pattern of people seeking healthcare services in the south. This is a difference between southern and northern Iran.

In the south, because of our future-oriented experience, and due to multiple empty spaces for various individuals for completing the egg-laying cycle, the southern mosquito itself can generate a higher number of patients compared to northern mosquitoes. The mosquito just lives—its number of blood meals corresponds to completing its egg-laying life cycle. In contrast, the northern mosquito feeds on both humans and animals, so less of its blood meals are taken from humans, and usually it takes only one blood meal per household per life cycle.

Therefore, the nature of the southern mosquito, in addition to access to diagnostic and treatment facilities, as well as cultural, economic, and social factors, inherently leads to a higher number of patients.

**Interviewer:** Thank you, Doctor. I appreciate your kindness. I have a question regarding the factors that influence the control of *Aedes* mosquitoes in the social and economic domain. I want to ask whether, by province, there is a difference? According to your experiences, province by province—

**Interviewee:** Could you repeat the question once?

**Interviewer:** I want to know. Some factors affect the spread of mosquitoes, and part of these factors relate to social and economic conditions, which you explained in general. Now I want to know, by province, whether these differ? From the perspective of social and economic factors, have you observed differences by province?

**Interviewee:** You see, we have an example. Cyrus County is a county that has fewer potential larval habitats compared to other counties in Hormozgan Province, such as Bandar Abbas or Bandar Lengeh. Also, the level of culture, education, and economic status of the people in Sirik County, Hormozgan Province, is higher compared to several surrounding cities. Therefore, although we captured mosquitoes there at the end of the year 1401 [2022–2023], the mosquitoes did not spread afterward and stopped.

In fact, the lack of mosquito growth in Sirik County compared to Bandar Lengeh County, Bandar Abbas County, and counties in Sistan and Baluchestan Province—Chabahar being the main example—is precisely because we observe the influence of culture, economy, education, and cooperation of the people.

Moreover, people in Sunni areas of the country, since they still live ethnically and tribally and have specific beliefs, listen more to their local leader, cleric, or elder. Therefore, in that county, where all residents are Sunni, we were able to use the community leaders to help with cultural education aimed at preventing the presence of active larval habitats in the county.

Of course, in metropolitan areas, such as Bandar Abbas, it is not as easy to get help from leaders, health volunteers, or influential people, because large cities like Bandar Abbas are metropolitan, with people from different nationalities, ethnicities, and counties across the country. So, these areas differ.

In Chabahar County, one of the main reasons for disease spread is the proximity to Pakistan and the frequent movement of people, with daily population exchanges easily entering our country, allowing transmission to occur. On the other hand, besides the disease naturally resolving, the necessary larval habitats in Chabahar are very abundant. This allows mosquitoes to develop and spread faster because urban planning, city facilities, and municipal services are weak, and people are in more vulnerable conditions due to the lack of urban sewage and stable drinking water, compared to cities in northern Iran.

Of course, in northern Iran, we still do not have experience of disease transmission and cannot speak definitively. However, if we compare Bandar Lengeh County in Hormozgan Province with Chabahar County in Sistan and Baluchestan Province, we can say that although local transmission of the disease started almost simultaneously in both provinces, it stopped in Bandar Lengeh. The reason is the lack of continuous population movement. Bandar Lengeh, although connected by sea to the UAE, experienced less impact because the intensity of the epidemic in the UAE is not comparable to the intensity of the epidemic in Baluchistan, Pakistan, which is at the border and has a greater influence than Bandar Lengeh, which is distant from Pakistan and connected to Arab countries.

If we move to Bushehr, I should mention that in Bushehr Province we have two counties, Jam and Dashtestan, where people are relatively more affluent, larval habitats are fewer, and mosquito abundance is lower. Compared to where? Compared to Asaluyeh and Kangan, in the same Bushehr Province. We observed that in Asaluyeh and Bushehr, mosquito population growth and spread were much higher than in Jam and Dashtestan counties.

**Interviewer:** Thank you very much, Doctor. I appreciate your kindness. I benefited greatly from your statements. For my final question, I want to ask: in your opinion, what are the appropriate strategies for mosquito control?

**Interviewee:** You see, we have two main principles in controlling mosquito-borne diseases. One is public participation, for two reasons. First, the origin and necessary habitats around human living environments—in fact, inside people’s homes or their yards. So, we must ask the people themselves, through cultural education, to reduce these habitats, because this problem will be permanent. In our country, this is not something that will end in a single day, unless one day a stable vaccine is developed nationally or globally. So, as long as we haven’t reached that point, engaging the public is the most important principle to promote.

After that, environmental improvement in urban areas can be undertaken using responsible urban organizations. This can include municipalities, urban water authorities, and all organizations involved in urban cleanliness and environmental improvement. Ultimately, the fundamental principle in reducing necessary larval habitats can occur in two ways: first, through public education and raising awareness; second, through actions that address the lack of necessary habitats. This can include providing stable drinking water, establishing urban sewage systems, creating proper wastewater management, and ultimately dredging or any measures in the city that ensure flowing water and prevent stagnant or standing water in our urban areas.

**Interviewer:** Thank you, Doctor. I appreciate it. If you have any other points or additional remarks, I am at your service.

**2^nd^ Interviewee (male)**

**Interviewer:** First, I will give a brief explanation about the study, and then, God willing, we will start the interview… Thank you. In this study, which was coordinated by Dr. Sargolzaei and other dedicated colleagues, the aim is to identify social determinants that influence the prevention, control, and spread of diseases transmitted by Aedes mosquitoes. This is being examined at the national level, but the focus is on four provinces: Gilan, Bushehr, Hormozgan, and Chabahar. The study is being conducted at the request of the Ministry and with the help of the Health Equity Research Institute, under the supervision of Dr. Oliaei Manesh.

This study consists of several sections, including review studies and qualitative interviews. God willing, all these results will be compiled together and can help in the prevention, control, and reduction of the spread of Aedes mosquito-borne diseases. The first part of the interview concerns the factors affecting prevention, control, and spread of the disease, especially regarding disease spread. According to the studies we have conducted, we have divided these factors into three general categories: first, environmental factors; second, social and economic factors; and third, factors related to health and treatment infrastructure. We want to know, according to you, what these factors are in each section, and if there are any other factors, we will mention them as well.

The final question will be about what strategies exist in each of these sections to control the disease and can be helpful, which we should take into account.

Now, the first part is about identifying environmental factors that play a role in the spread of Aedes mosquito-borne diseases. In your opinion, what factors in the environment can influence the spread of Aedes mosquito-related diseases in the country?

**Interviewee**: From a technical perspective, if I want to explain, environmental factors are those whose effects actually depend on the species. In Iran, we currently have two invasive species: *Aedes albopictus* and *Aedes aegypti*. As you know, *albopictus* is found in the north of the country, while *aegypti* is in the south. These species can live in different environments. Of course, *aegypti* has a slightly wider range. That means, for example, you might expect to see *Aedes aegypti* in northern provinces, but the likelihood of finding *Aedes albopictus* in southern provinces is lower. In other words, you can almost be certain that *albopictus* will not be present there, because it tends to prefer higher latitudes, while *aegypti* does not.

The point here is that because these are insects that lay eggs in water—especially unlike malaria mosquitoes, which can lay eggs in deep or large water bodies—these species can lay eggs in shallow and small water collections. This gives us a hint: usually in places with high rainfall and many small ponds or puddles, like countries such as Malaysia and Thailand, which have heavy rainfall throughout the year, these mosquitoes are abundant. But in our country, due to the dry and semi-dry climate, in areas like the north—where rainfall used to be higher—you could expect to see these mosquitoes year-round.

In the southern regions, however, there is a specific characteristic: rainfall occurs in a limited season. Therefore, we have fluctuations in mosquito abundance throughout the year. Their numbers increase during the rainy season because ponds and wetlands form, and during the warm or dry seasons, when there is little rainfall, abundance decreases. This is, of course, a prediction.

Their breeding sites are limited to water containers that, as I mentioned, people use as water sources. In the south, there is some difficulty in water supply, so people store water in various containers. These containers become the breeding grounds for mosquitoes. Therefore, in the warm seasons, since we do not have natural wetlands or ponds in the south, the area of mosquito proliferation is smaller. This actually gives us an advantage compared to other countries: if we control the water sources that people store, we can significantly impact the disease vector.

Since I am an entomologist, I cannot really comment on the disease itself. Because you want to conduct the interview and later draw conclusions, my statements could introduce bias. So let me focus on the vector, which is my area of expertise. This gives us an advantage: as I mentioned, by controlling these sources, we can address the problem—either by managing the water containers or by recommending to urban service departments and water authorities to provide stable and safe drinking water for people, so they always have access to potable water. Then they can use tap water for drinking, washing, and cooking, and they won’t have to store water.

I don’t think there’s any other point to add regarding this. I think geographical location matters. Of course, in the central parts of the country, due to very, very low rainfall, we usually have fewer problems. Personally, as an expert in the Ministry of Health, I am not worried about the central regions of the country. But the southern and northern regions are different. A map was also released by a company called Tiz Negar {I’m not sure about the correct spelling of the company’s name}. When you look at that map, in the central areas, the distribution is very small, almost near zero. Most of our problems are in the northern and southern margins. The northern margin is due to sufficient rainfall, and the southern margin is because of the water resources there. Naturally, all of these are influenced by environmental factors. Humidity also has an effect.

Moreover, the behavior of *Aedes albopictus* differs somewhat from *Aedes aegypti*. In entomology, we describe *albopictus* as exophilophagic. Exophilophagic means it prefers the outside. That is, it favors outdoor environments over indoor ones. Even if you define an indoor place with four walls and a roof, it still prefers the outdoors, and it also bites outside. Another difference between these two species is that *Aedes aegypti* is anthropophilic, meaning it prefers humans. It only feeds on humans. In contrast, *Aedes albopictus* is not strictly anthropophilic; it is also zoophilic, meaning it can feed on both humans and animals. This gives *albopictus* a relative advantage in spreading, while *aegypti* is very limited and lives close to humans because it is endophilic, endophagic, and anthropophilic. These three characteristics impose limitations, making control more difficult.

*Aedes albopictus*, however, is exophilic, exophagic, and also shows some zoophilic behavior, which allows it to spread more widely. This gives it a relative advantage and changes the approach and quality of vector control compared to *Aedes aegypti*. I mainly focus on environmental conditions for mosquito distribution, which are largely influenced by species type, i.e., behavior and habits of the mosquito. In my opinion, species type is more important for the spread of these insects than climatic conditions.

To give a more practical example, consider the climatic conditions in East Azerbaijan Province versus Gilan Province. These two are very different. East Azerbaijan has a mountainous, non-forested environment, whereas Gilan is fully forested. So, the distribution cannot be attributed solely to climate. It is mostly determined by mosquito behavior and species-specific traits. The species in East Azerbaijan is actually similar to the species in Gilan.

Regarding humidity, I would say that in southern regions, the mosquitoes can still be present in areas without much humidity, as long as there is some water. For example, even in places like Tehran, we could expect to see *Aedes albopictus*. If you try to correlate mosquito presence with humidity using a positive correlation or Pearson coefficient, it might not even work in Iran.

It was interesting that you added a new perspective regarding mosquito behavior; I found that very valuable. Regarding this topic, let me just pause here…

**Interviewer:** Thank you very much, Doctor. Well, my next question for you is: what social and economic factors influence the spread of mosquito-borne diseases? Economic factors?

**Interviewee:** Actually, there’s a discussion here. Economic factors, as you might think, do not have a direct effect on this issue, especially on the disease itself; they have an indirect effect. You see, people who are economically well-off… Let me mention that Interviewer told this lady to come at ten, and now she has arrived. Economic factors are important in this sense: a well-off person usually has a large house, low population density, and economic advantages such as air conditioning. People who are less well-off have higher household density because their living space is smaller. And, let me add, they often don’t have air conditioning.

When discussing serious issues, one of the effective factors mentioned is air conditioning. Why? Because when you turn on the AC—whether it’s a gas cooler or a chiller and fan coil, etc.—you usually close doors and windows. When doors and windows are closed, mosquitoes cannot enter the house. So, if population density is high, if one mosquito enters a low-income household, for example, it can bite a family of ten people in one night. But in a well-off household of four people living in a 120–150 square meter house, the same mosquito might bite only one person in a night. This is the difference.

However, if we were to say that this disease is absolutely related to economic status in society—no, there is no such direct correlation. There is an indirect relationship between economic status and disease prevalence.

On the other hand, I was talking about… I forgot. We were discussing economic factors, and that’s done. Social factors also exist. Socially, people who are well-off and financially capable tend to take better care of themselves. What does that mean? It means that if they feel even a little discomfort, they immediately go to a doctor and try to follow healthcare recommendations. Meanwhile, people who are less well-off have other problems, and health is less of a priority for them. For example, in Chabahar, some people get sick but do not go to a doctor, either because they don’t have the money, they are too exhausted, or they have so many other hardships that they cannot spend the time to see a doctor and get treated.

In fact, what does this result in? It results in patients who are completely unrecognized because they never seek care. They might have contracted dengue fever but did not go to a doctor. When you examine this, you see that these people mostly come from social classes that are politically and socioeconomically lower. Due to their economic situation, people with weaker economic status generally visit doctors less frequently.

**Interviewer:** Doctor, in your opinion, does their awareness that they should seek care also have an effect?

**Interviewee:** I mean, for example, economic status might have some effect, but consider a person who has a thousand and one problems and doesn’t even have enough bread to eat. Let me add, their child is also sick. For example, their child has a problem, or they don’t have money to buy shoes for the child. Also, imagine they have a house, part of which is damaged or collapsed. I don’t know… Have you been to Chabahar? No? I said I went to Zahedan. If you go, in my opinion, talk to Dr. Oliaei Manesh. Since you are a researcher on this topic, go see the area. One of the future control methods is to put screens on windows. Then you go and see an area called Muradabad or Janglook in Chabahar. I said, if you can find a window to put a screen on, I’ll change my name. There is no window at all because they all get blown away.

And what does this lead to? It causes the person living in a hut to be more exposed to disease and more at risk of bites. And due to their economic situation, you cannot expect to educate them or for them to practice self-care, because they have so many other problems that self-care is the 20th, 30th, 40th, or 50th priority. They have to feed their children, take care of their family, take care of their elderly parents. These problems cause them to be more exposed to disease.

However, when you look at the disease distribution map, you see that there is almost no difference between well-off and less well-off areas. Of course, if you go to major crossroads like Tehran—north and south—you don’t see the same pattern. For instance, in northern Tehran, like Qeytarieh, versus, say, Nazyabad in the south—although Nazyabad has improved now, and Khaniabad in the south, etc.—you can see very different neighborhoods. Next to a multi-billion-toman marble palace, you might find a hut. These issues make social and economic differentiation more difficult.

Do you understand my point? For example, in Tehran, you could go along Enqelab Street: one direction, you might see more loosely veiled or unveiled people; the other direction, more veiled, modest individuals. That used to allow some differentiation. But in Chabahar, you cannot say which neighborhood is well-off and which is underprivileged because rich and poor live side by side.

Do you know why? Twenty to twenty-five years ago, everyone in Chabahar was similar—economically disadvantaged. Now, due to the city’s economic growth, the free trade zone, and the situation with fuel smuggling—now many city billionaires are fuel smugglers—you see a very polished marble palace worth tens of billions next to a poor hut. Even in southern Tehran, the lowest areas of the city outskirts do not allow such clear social-economic differentiation.

So, when we looked at the country’s disease map, it didn’t show much distinction. But, as I told you, economically, these factors can indirectly affect the number of bites and, consequently, the rate of disease transmission.

**Interviewer:** Thank you. The next question is, in your opinion, do healthcare and treatment infrastructures also have an impact on disease transmission or control, or is it not the healthcare infrastructure?

**Interviewee:** Primarily, it can have an impact when there is a patient. It depends on how you define infrastructure. If by infrastructure you mean the physical facilities and equipment of healthcare and treatment, I don’t think it has any impact on disease transmission. But if you consider healthcare infrastructure as the healthcare services themselves, meaning, for example, if an active service is being provided—like active case detection, conducting epidemiological surveys, collecting blood samples, testing, and then determining whether someone is neurologically affected, infected, or not—then yes, it has an impact. It entirely depends on how you define infrastructure.

**Interviewer:** I meant both the first and the second cases……….Yes, the second one. That is, we divided the infrastructure into parts: one part related to healthcare programs and policies, and the other part is healthcare centers, human resources, production of equipment, and also healthcare policies and programs.

**Interviewee:** Exactly. Look, in terms of equipment and facilities—let me just close the door for a moment—having a clinic for treating patients is good, but it doesn’t affect preventing disease transmission or controlling mosquitoes. However, when it comes to healthcare personnel and staff involved in mosquito control, including disease control teams, entomologists, or environmental health staff conducting environmental improvement operations, if their numbers are sufficient, their salaries are adequate, and they have the necessary equipment to perform environmental health operations properly and on time, then yes, this is the foundation and backbone of Aedes mosquito control operations.

Because, as I explained, there are environmental factors, and if we can control these factors, the mosquito population can be controlled to a large extent. However, if this infrastructure does not exist, if the personnel are insufficient and cannot cover the city adequately, then the control operations cannot be carried out properly. This affects mosquito abundance and biting rates.

If active case detection is conducted and precise cellular and biological investigations are performed, this can impact disease prevention programs—for example, the distribution of insecticide-treated nets, window screens, or environmental health surveys. These infrastructures help enable effective control.

**Interviewer:** Thank you very much. In this section, I would like to know whether, in the different regions of the country—where we are focusing on four provinces: Gilan, Bushehr, Hormozgan, and Chabahar—do you think the social infrastructures in these cities differ from each other? And how have these differences influenced things?

**Interviewee:** There is a very, very big difference. I’m telling you, go see for yourself. Let me ask, is Hormozgan included in your program?

**Interviewer:** Yes, yes—Bushehr, Hormozgan, and Chabahar.

**Interviewee:** For example, you don’t have work in the northern provinces; you are focusing on the south, which has similar species. The situation in Bushehr and Bandar Abbas is not comparable at all to Chabahar. Bandar Abbas and Bushehr are developed cities, organized and orderly, whereas, let me tell you, Chabahar’s urban area is a town that has recently become a city, and it is just emerging from economic backwardness. People are just beginning to understand what life means. I’m not talking about the new city—the part where investors and wealthy people live—that’s aside; you can see similar areas in northern Tehran. I am referring to the old city. Let me tell you, these are completely different, and this difference causes them to have different conditions.

For instance, let me clarify—I cannot directly link this, but economically, they are not comparable at all. Now, let’s put aside the Chabahar issue. If you go to Konarak, we don’t have a problem with disease transmission there. You go, and you wonder—can such a place really exist in our country? It’s not a matter of rich or poor; it’s about social structure and urban layout. For example, in Bandar Abbas, you won’t see a single dirt road, but in Chabahar, the streets have just been asphalted, and the alleys are still mostly dirt. One of the recommendations I made to the environmental health staff there was to start an asphalt movement to pave the alleys.

You might ask, what impact does it have? Does it have an impact? Well, not a direct effect on mosquitoes—you won’t easily notice the larval habitats. But in terms of SDH (social determinants of health), when people see the street in front of their house paved, they feel they have a higher social status. It has an effect: they don’t litter or they collect their trash to dispose of it properly. However, if you go there during the day, for example, to the market when it’s crowded, you may not notice much. But if you go at night, you’ll see people throwing whatever they ate or whatever they had in their hands on the ground. Streets are full of trash, and if the municipality doesn’t clean it, in two or three days, the garbage piles up. Or there was a central canal in the city. Early on, we noticed that this canal, meant for flood control and waste disposal, runs through the middle of the market. We told people to clean it, and some parts were cleaned. On our next visit, we saw that people had thrown trash again. How many times must the municipality clean it? Why? Because people see no difference—whether they throw trash here or there, nothing changes. If you visit a very dirty house, your own cleaning behavior will likely decrease. But if you go somewhere very clean and orderly, psychologically, you try not to litter, and if you do produce waste, you try not to make your home messy or dirty. These are the types of behaviors.

Another problem that Chabahar has, which our other cities do not, is the issue of migrants. Pakistani migrants move there without control, which disrupts the city’s situation. This is a problem we don’t have in Bandar Abbas or Bushehr, because those cities are farther from the Pakistan border, and the security and police controls are stricter within the city. But in Chabahar, due to cultural similarities, it is difficult to distinguish a Pakistani from an Iranian, especially based on their clothing—they look almost the same. This is one of the problems Chabahar typically faces.

Thank you. I don’t know why, but I feel my explanation may have been a little unclear due to the comings and goings that occurred here.

**Interviewer:** No, that was very good. I have now recorded your points with the voice recorder, God willing. I will extract them all one by one. If any adjustments are needed, I will make them. Just share all your points as they are—that’s excellent. Thank you, Dr.

Regarding the health and treatment section, I now understand the economic part. You also mentioned the differences in social adoption, that these vary. Concerning the treatment infrastructure, in Gilan, we said it’s different because of the economy. Is there any other reason why the health and treatment infrastructures and programs in the southern part of the country differ from those in the north? Is it influential?

**Interviewee:** Yes. Look, this discussion is one thing; there’s another thing as well. Now, I hope that no one other than you hears this interview. I don’t know whether to say it or not. See, Chabahar has been very neglected. Not neglected by the central government—the poor central government provides its facilities, meaning it makes the effort itself. What’s important is that the local officials there are responsible. Well, my request is that I decided to mention this, but please do not disclose it anywhere. Just hear it. Never mind, forget about it—it’s not important.

But just to say this much: until now, it has been somewhat neglected, and that region has been a little overlooked. One gets the feeling that it’s almost as if these people aren’t even in Iran. Yes, well, I also say it’s not the central government’s fault. Don’t worry about whose fault it is. It’s not the central government’s fault. If they had been asked, they would have provided. They weren’t asked. They gave it to us, thinking the situation there was good. Do you understand?

**Interviewer:** Yes, I understand.

**Interviewee:** Yes. For example, if hypothetically they don’t have a loader—or maybe they have very few loaders—this is because the government didn’t provide it or no one requested it from the government. It requires follow-up by the local officials. If they had said, “I need a loader,” they didn’t. They didn’t say, “I need a truck,” either. And this has caused the city to develop disproportionately and asymmetrically.

Now, if we want to say it has experienced asymmetric development, you go to the free zone and you see a European-style city, similar to northern Tehran’s old city. I’m saying you didn’t see it twenty years ago—twenty years ago, it barely existed. Now I went, and I was very happy. Chabahar today is completely incomparable to twenty years ago.

Let me tell you: in the old city, there are numerous problems, and this has caused—you know the famous photo of Brazil and Rio de Janeiro, where they built one wall, and one part is like small cities while the other part is all slums? It’s like that. When you go there, you can see the difference between urban fabrics, the distinct neighborhoods. Now, this imbalance is gradually improving, although the gap is still large. But many reasons can be cited for this issue, and primarily it is not economic.

For example, in Chabahar, I can show you people who earn ten times more than a billionaire in Tehran. I don’t care what their occupation is. Their subcultures are somewhat different from ours. Therefore, one has to adjust their perspective to match their conditions. See, if you try to view Chabahar with the perspective of someone from Tehran or from a large city, you will introduce some bias. Right? Therefore, you need to be contextually appropriate. When I go, for example, to Chabahar, I look at it from a Tehranite’s perspective, but later I look at Chabahar from Chabahar’s perspective itself.

**Interviewer:** Exactly, that’s why they say any action we want to take should always be adapted to the culture. It should be in accordance with the culture of that region. Very well. Thank you very much, Dr. Now, my last question concerns the strategies that can be applied for the prevention, control, and spread of diseases transmitted by Aedes mosquitoes. In your opinion, in each section—environmental and environmental factors—what strategies do you think could be useful? In the social and economic section, and then in the health section? And if there are any other factors, we are here to listen to you and receive your recommendations.

**Interviewee:** As I mentioned before, the problem in Chabahar is an SDH problem. Because, for example, in Bandar Abbas, you encounter fewer social determinants of health than in Chabahar. That is, in Chabahar, you have to work more on SDH issues. For instance, I told our colleagues in Chabahar, I said, “For example, force the municipality to plant flowers.” They responded, “Well, what impact do flowers have?” I said, “When people see their city turning into a more modern, clean, and orderly city, they cooperate more.” You can’t go to someone in a dilapidated city and say, “Sir, follow hygiene practices.” They say, “First, fix your own city, and the part that concerns me is the municipality’s responsibility. Go fix them first, then come to me. I don’t have water to drink; I only have water two days a week, and even then, only for two hours in some parts of the city, so I have to collect water in containers. What are you talking about?” Therefore, our focus in Chabahar should be on SDH. But your topic is not Chabahar. For example, in Bandar Abbas, SDH is not that important. People are affluent and have sufficient resources. We can focus on other factors.

One thing observed in Chabahar, usually in differences—I don’t know the reason, and even if I did, I think it’s better not to say—is that people cooperate less with the health system. For example, when you tell them, because one of the principles of Aedes control is community participation, in Chabahar you can rely less on community participation compared to Bandar Abbas, Bushehr, or Gilan. On the contrary, when you want to carry out mandatory spraying in homes, people in Chabahar allow it more easily than in Gilan or Bandar Abbas. Why? Because they are more familiar with the malaria program. They think it is still the same malaria program and are used to always letting the spraying pump go into their houses and spray walls. That’s also a method. But here, in Bandar Abbas or, I don’t know, Gilan, people do not allow this at all.

You see how these factors socially affect the control program. For example, if you fog in Chabahar, no one comes to ask, “Excuse me, what are you doing?” But when you fog in Astara, Rasht, or Bandar Abbas, people ask, “What is this smoke you’re generating? Why are you doing this?” These are social differences that can affect our program.

There are various methods. One major focus is community engagement, especially in environmental management for Aedes aegypti, because, as I mentioned, it usually lives close to human settlements. For Aedes albopictus, our problem was solved by sending health personnel to clean streets and sweep regularly. But in areas where breeding occurs inside houses, health personnel cannot access the interiors. If they cannot access the houses, what does this imply? It means that you and I, whose houses might be involved, must be aware and understand where active larval habitats might exist. Do you understand my point? Yes. And this increases the importance of community engagement for Aedes aegypti. The example with Aedes albopictus is somewhat less, but it is still important. In Aedes aegypti, community engagement has a very high impact, and if the community does not help, our social problem is much harder to solve than the Aedes albopictus problem. That is, the importance of community engagement still depends on the mosquito species. This does not mean that I am saying community engagement is unimportant for Aedes albopictus or that it is important here. But for serious future concerns, its importance is far greater.

For example, if someone like me comes to your house uninvited and says, “I want to check your house,” you would never allow it. They resist unless some enforcement is involved. There are other methods for control, like spraying, environmental management, and individual measures, such as distributing insect repellents, mosquito nets, or environmental improvements like installing screens. The most important part, when looking at control systems, is environmental management, which largely depends on community engagement. Then, depending on whether your scenario is three-stage, six-stage, or eleven-stage, other methods like space spraying or fogging are applied.

Friends, in my opinion, if you really want to see the impact of SDH on the disease, you must visit Chabahar. I don’t want you to do anything specific—just walk in the streets, observe the people, and then you will understand how crucial SDH is for health.

**Interviewer:** Thank you. I think you have about six minutes, Dr. If you have any other comments, we are here to listen. If you also have suggestions regarding health infrastructure, health programs, policies, or social factors, we are ready to hear them.

**Interviewee:** No, I have almost said everything. I wish you success. Thank you very much. I hope it will be a good study, with reliable results that can be used when planning and implementing actions in the community. Our goal is also to provide service to the community. Thank you for your time and kindness. Thank you.

**3^rd^ Interviewee (male)**

**Both:** Hello, hello. Please go ahead. How are you? Thank you for your efforts. Thanks, please go ahead. Good work. Thank you, professor.

**Interviewer:** Sorry, I think you must be tired too, but I’ve already disturbed you. I apologize. First, professor, I’ll review the study objective. The purpose of the study we are going to conduct—you’ve read it yourself, you know it. Yes, yes. Well, I’ll let you tell the rest, but say it, then the question comes in.

The first question is: In your opinion, what environmental factors influence the spread and prevalence of mosquitoes?

**Interviewee:** The Aedes mosquito, which is in fact the vector for dengue fever, is one of the most important factors affecting and reproducing environmental health issues, especially in places with stagnant water and damp areas. You can say that during times of rain and similar conditions, when water collects in ponds and puddles, these are the best places for laying eggs and observing them. For this reason, basically, our coastal provinces, the ones currently affected—Gilan, Bushehr, Hormozgan, Sistan and Baluchestan, Chabahar, and so on—are impacted, and environmental health is a major factor. Truly, one of the places they lay eggs is in our humid provinces, in things like water tanks, old tires stored in warehouses, and in places where the population grows. This is why the most important factor is environmental health, essentially.

**Interviewer:** Yes, professor. Regarding this issue, in your opinion, are there other factors such as environmental health measures you mentioned, stagnant water, humidity, temperature, housing, or similar factors that are influential?

**Interviewee:** Certainly. See, overall, because this problem mostly occurs in places that are structurally and in terms of welfare lower than other areas, usually even in these four provinces, it happens in regions with less welfare and where non-standard spaces exist. Therefore, it is completely related to poverty and the lack of economic and social infrastructure, and you could say it is also connected with social anxiety and broader economic issues, as well as individual problems. That is, individual poverty and areas with low environmental health, low welfare, economic and social infrastructure, and where poor people live—these are generally the areas where mosquitoes are more prevalent. Yes, that’s correct. Therefore, it can be said that it is associated with a spectrum of individual-level and social-level factors.

**Interviewer:** Very well. Professor, in your opinion, regarding environmental factors in the environment section, in these counties you mentioned and which are the focus of our study, do you think there is any difference or not? For example, are environmental factors in Chabahar different from Hormozgan and Bushehr different from Gilan?

**Interviewee:** One point is that this mosquito was first imported. That is, did we get infection from the Dubai influx? Yes, for this reason, the southern provinces were affected first, and then it spread to Gilan province. Perhaps its first entry wasn’t because of these issues but was related to the Dubai flood. After that flood in Dubai, this mosquito spread in Dubai and then was imported into Iran by people who traveled frequently. Unfortunately, now in some cities, local transmission has occurred due to egg-laying and physical spread within the country, but its start was good, and that’s why it happened this way. Our first cases were mostly not from people who were poor or lived in unsuitable places but who traveled frequently to Dubai. But our later cases, why? This data is becoming similar—mostly in places with unsuitable environments, and it is happening there.

**Interviewer:** Very well, thank you, professor. My next question: Professor, you mentioned that economic and social factors are also influential on the spread of this disease. Could you explain more for us how these factors influence it and how individual issues and social issues relate to these public structures?

**Interviewee:** Naturally, it’s like this: someone who is poor lives in a place where the welfare structures are unsuitable and inadequate. Yes, in fact, the government also reaches these areas less, and unfortunately, individual poverty is intertwined with living in unsuitable housing and poor environmental conditions. All of these are connected. It’s not like someone is poor but lives in a place with very good systems. Usually, where the government provides fewer facilities, the person is forced to live there due to rent or similar reasons. It’s all interconnected—a completely interrelated system—and most social and economic factors and social harms accompany these conditions.

**Interviewer:** Yes, dear professor. Regarding the economic aspect, could it be, for example, related to water storage? Right?

**Interviewee:** It could be related to these facilities that prevent mosquito spread, such as public infrastructure. For example, the absence of a sewage network and similar issues, because surface water remains mostly in places where sewage infrastructure does not exist. These are connected with unsuitable environments, poorer neighborhoods, city outskirts, and slums. These elements are all interconnected.

**Interviewer:** Yes, very good. Regarding social factors, in your opinion, does health literacy also have an impact?

**Interviewee:** Absolutely, absolutely. But one thing that can prevent the spread is people’s health literacy and providing this information to the public. Unfortunately, there are thousands of people regarding the relationship between poverty and illiteracy. Of course, with social media, public health literacy has improved somewhat superficially, but overall, it is completely related: someone who is poor also has lower health status and fewer opportunities to access correct information in this area. So, it is related to the social issue of literacy and health, even the level of literacy—that is, education level.

**Interviewer:** Yes, correct, professor. Regarding economic and social factors, the question I want to ask is: Is there any difference among these counties in terms of these economic and social factors? Among these provinces?

**Interviewee:** Well, these four provinces are all humid provinces. That is, they have a lot of rainfall, which is a common factor among all four. Let’s leave aside the initial cases that came from Dubai, but now it can be said that it started in the humid provinces, and perhaps in places with heavy rainfall and incomplete sewage systems, it spreads. Now, I don’t think there is much difference among them, as it can be assumed that the unsuitable and deficient areas in terms of infrastructure in all four provinces are similar. Of course, Gilan is somewhat less, but it exists there too. It has also become prevalent there.

**Interviewer:** Yes, that’s correct, professor. Regarding economic and social factors, the question I want to ask is: Is there any difference among these counties in terms of these economic and social factors? Among these provinces?

**Interviewee:** Well, all four of these provinces are humid provinces. That is, they have a lot of rainfall, which is a common factor among all four. Let’s leave aside the initial cases that came from Dubai, but now it can be said that it started in the humid provinces, and perhaps in places with heavy rainfall and incomplete sewage systems, it spreads. Now, I don’t think there is much difference among them, as it can be assumed that the unsuitable and deficient areas in terms of infrastructure in all four provinces are similar. Of course, Gilan is somewhat less, but it exists there too. It has also become prevalent there.

**Interviewer:** Thank you, professor. In addition to environmental, social, and economic factors, according to the studies we reviewed, there are some factors related to health and medical infrastructure and the facilities, as well as health programs and policies. I wanted to know, in your opinion, how can these factors influence the spread of the Aedes mosquito that our health network deals with?

**Interviewee:** Raising awareness among the people. And people who are less informed also have fewer visits. Some don’t trust, they don’t have time, they don’t believe, and other reasons. But definitely, in my opinion, in these areas where it has become prevalent, there are two aspects: either our centers are fewer, or if people visit less, it is still completely effective. The existence of an active health network reduces the incidence of this disease.

**Interviewer:** Yes, thank you, professor. Thank you. As my last question, I want to ask: In your opinion, what strategies can be used to control the spread of this insect?

**Interviewee:** Look, the government has certain responsibilities, and ultimately the public also has responsibilities. There are duties for us as the health sector to provide governmental infrastructure in deprived areas, addressing sewage issues, and properly handling water accumulation and puddles in the streets. Citizens’ duties are to have sufficient information and perform preventive actions. So, there’s a spectrum of interventions. Our duty is also supervisory work, along with raising public health literacy and carrying out environmental health measures in places where facilities are inadequate. For example, as I mentioned, places where old tires are stored. One of the most common places is exactly this: old tires where water has accumulated. You can almost 100% say that this is a mosquito breeding site.

**Interviewer:** Yes, that’s correct, professor. In the studies we reviewed, they also pointed exactly to this issue: through trade and imports, it entered the country, and then those second-hand, old tires became the main accumulation sites. Very well, professor. If you have any other points, another factor, or another strategy, I am at your service to hear them.

**Interviewee:** Look, in this study, this study itself may reveal other factors, and I hope it does so that human knowledge in this field increases. After all, factors discovered in other places may be discovered here. Environmental, social, economic, individual factors—we should look for ways to add new knowledge to human understanding in these areas, right?

**Interviewer:** Yes, for example, when we conduct interviews, exactly a few people from the ministry said that we went to various cities, for instance, different groups of 800 or 900 people, and examined where the prevalence was higher. The information from these individuals was collected through the interviews.

**Interviewee:** You can conduct interviews in these four provinces as well, both with the public, with health personnel, and with government managers and so on.

**Interviewer:** God willing, professor. Thank you very much, professor, for your kindness. Thank you. Have a good evening. Goodbye.

**4^th^ Interviewee (male)**

**Interviewer:** Hello, **Dr.**, how are you? I hope you are well. Thank you for your efforts. Shall we start the interview, **Dr.**?

**Interviewee:** Yes, yes, yes, very well.

**Interviewer:** Just a moment, let me check—my mobile voice recorder can record simultaneously, or does it need to be on the laptop?

**Interviewee:** It cannot; it should be on the laptop.

**Interviewer:** Very well. I will record your voice on the laptop. Excellent. First, I want to thank you for giving us your time. We are very grateful. Surely, your words are extremely valuable and important. God willing, we will be able to make very good use of them.

This study is being conducted to identify the social determinants affecting the prevention, control, and spread of diseases transmitted by the Aedes mosquito. It is being carried out under the order of the ministry and through the Center for Health Justice Research under the supervision of **Dr. Oliaei Manesh**. Several review studies have already been conducted, but for a qualitative study to collect the opinions of experts, professors, and people involved in this field, a qualitative approach was necessary. In this interview, God willing, we aim to answer four main questions.

The first part relates to the first question: the factors affecting the prevention, control, and spread of Aedes-transmitted diseases. Based on studies and reviews, we have divided these factors into three categories: environmental factors, social and economic factors, and factors related to health and medical infrastructure, programs, and policies. The fourth question concerns what interventions can be employed to control this disease.

So, **Dr.**, if there are no particular questions you have for us to raise first?

**Dr.:** Thank you. I greet you and all your colleagues. I appreciate that you invited me to be at your service. I hope I can provide correct answers to your questions.

**Interviewer:** Certainly, it will be. We are also grateful to you. Thank you, **Dr.**. The first question is: in your opinion, what environmental factors play a role in the spread of Aedes in the country?

**Interviewee:** Well, these factors need to be categorized. The most important environmental factor influencing the prevalence and spread of the Aedes mosquito—its abundance, growth, reproduction, and increase in the community—is related to climate changes. That is, if we look back forty or fifty years at the status of Aedes mosquitoes worldwide, we see that certain regions, mainly tropical regions, were affected by mosquitoes and were areas where mosquitoes could grow and reproduce. Gradually, however, the mosquito changed and expanded its habitat. It has now reached a point where not only tropical regions but also subtropical regions of the world are affected by the growth and reproduction of these mosquitoes. Gradually, the number of countries exposed to the growth and spread of this insect has been increasing. Now it has reached a point where nearly half of the different regions of the world are exposed to this mosquito. Naturally, if the virus causing the disease—dengue fever—grows and reproduces inside the mosquitoes, it can spread the disease. Therefore, climate changes have played and continue to play a very important role.

The second issue is urbanization. When we talk about urbanization, we mean unplanned, uncontrolled urbanization, where cities have rapidly accommodated large populations over the years without having the necessary health facilities and infrastructure. This has caused cities to be more prepared for mosquito growth and reproduction.

The third issue is the behavior of the people in these areas. Obviously, populations that do not maintain the hygiene of their living environment increase the likelihood that this disease will spread through mosquito growth and reproduction. There are also various other factors in this context, each of which can act as an environmental factor contributing to the spread of this disease.

**Interviewer:** Thank you very much, **Dr.**. There are a series of factors such as climate, vegetation cover, stagnant water, and then the design of the city or residential areas, temperature, humidity, and waste. In your studies and investigations, do you think the factors I mentioned were influential or not?

**Interviewee:** Yes, absolutely. I said that climate changes, global warming—especially in certain regions of the Earth—rainfall increase, decrease, both increases and decreases, as well as, as you mentioned, vegetation cover, each of these can act like pieces of a puzzle in their own way and create conditions for the spread of this disease and for the growth and reproduction of the mosquito that transmits it.

**Interviewer:** Thank you. From these two, in this section, I have a question for you: we are focusing mainly on four provinces, including Gilan, Bushehr, Hormozgan, and Chabahar. We are studying these areas specifically. In your opinion, do environmental factors in these counties and provinces cause differences that might make the prevalence of Aedes different across these locations?

**Interviewer:** Certainly, it will be. We are also grateful to you. Thank you, **Dr.**. The first question is: in your opinion, what environmental factors play a role in the spread of Aedes in the country?

**Interviewee:** Well, these factors need to be categorized. The most important environmental factor influencing the prevalence and spread of the Aedes mosquito—that is, its abundance, growth, reproduction, and increase in the community—is related to climate changes. That is, if we look back forty or fifty years at the status of Aedes mosquitoes worldwide, we see that certain regions, mainly tropical regions, were affected by mosquitoes and were areas where mosquitoes could grow and reproduce. Gradually, however, the mosquito changed its habitat and expanded further. It has now reached a point where not only tropical regions but also subtropical regions of the world are affected by the growth and reproduction of these mosquitoes. Gradually, the number of countries exposed to the growth and spread of this insect has been increasing. Now it has reached a point where nearly half of the different regions of the world are exposed to this mosquito. Naturally, if the virus causing the disease—dengue fever—grows and reproduces inside the mosquitoes, it can spread the disease. Therefore, climate changes have played and continue to play a very important role.

The second issue is urbanization. When we talk about urbanization, we mean unplanned, uncontrolled urbanization, where cities have rapidly accommodated large populations over the years without having the necessary health facilities and infrastructure. This has caused cities to be more prepared for mosquito growth and reproduction.

The third issue is the behavior of the people in these areas. Obviously, populations that do not maintain the hygiene of their living environment increase the likelihood that this disease will spread through mosquito growth and reproduction. There are also various other factors in this context, each of which can act as an environmental factor contributing to the spread of this disease.

**Interviewer:** Thank you very much, **Dr.**. There are a series of factors such as climate, vegetation cover, stagnant water, and then the design of the city or residential areas, temperature, humidity, and waste. In your studies and investigations, do you think the factors I mentioned were influential or not?

**Interviewee:** Yes, absolutely. I said that climate changes, global warming—especially in certain regions of the Earth—rainfall increase, decrease, both increases and decreases, as well as, as you mentioned, vegetation cover, each of these can act like pieces of a puzzle in their own way and create conditions for the spread of this disease and for the growth and reproduction of the mosquito that transmits this disease.

**Interviewer:** Yes, thank you. From these two, I have a question for you in this section. We are mostly focusing on four provinces of the country, including Gilan, Bushehr, Hormozgan, and Chabahar. We are examining these areas specifically. In your opinion, have environmental factors in these counties and provinces caused changes such that the prevalence could be different in these counties?

**Interviewee:** Look, for example, the World Health Organization had predicted this previously. Both the northern strip of the country and the southern strip—the coastal regions—were accurately predicted as areas where the mosquito would readily grow and reproduce. We had made these predictions ten to fifteen years ago and had also taken some measures, although perhaps there isn’t space for a detailed explanation in this interview. But it was completely predictable due to climatic factors.

Alongside that, I mentioned people’s behavior and increased urbanization. Cities really lacked necessary health facilities. Among the cities of Gilan province, as well as the cities you mentioned—Chabahar, Bandar Abbas, Bushehr—the health infrastructure and environmental hygiene were not very strong. For instance, waste collection is one of the most important factors in Aedes mosquito growth and reproduction. To prevent this, municipalities would have needed to organize waste collection over time in a systematic and proper way. But such organization has not occurred over past years.

Drought has also been a very influential factor. When there is a drought, people are forced to store water. If you have visited the southern provinces, in various cities, you see that above almost every home there is a water tank. If this water tank is open, it is one of the most important places where Aedes mosquitoes can grow and reproduce. The more these household water sources exist, the higher the probability becomes. Similarly, sewage systems, waste collection, and the proper use of cooling devices in southern regions—without necessary standards—have all been influential factors that have put these provinces at higher risk.

I mentioned that Gilan province was previously predictable, and it is also predicted that Mazandaran and Golestan provinces will likely face growth, spread, and reproduction of this mosquito in the near future.

**Interviewer:** Thank you very much, Dr. Thank you, your explanations were very complete and comprehensive. Thank you. Regarding social and economic factors affecting the prevalence of Aedes-transmitted diseases, what is your opinion?

**Interviewee:** Well, in any case, one of the most important social influencing factors is the issue of poverty. It is obvious that this disease in poorer areas, in regions with more limited resources, will certainly spread more quickly and its likelihood will be higher. One of the most important influencing factors is the issue of poverty. Another factor is the insufficient education of people. It is obvious that now, in countries dealing with this disease, planning for this issue—one of the most important things they pay attention to—is educating the public. If people, especially the younger generation, are educated, they can play a vital role in control and containment, which I think can play a very fundamental role.

**Interviewer:** Thank you, Dr. Regarding social factors, you mentioned earlier that literacy was one of them, right? Now I want to know, for example, is the culture of the northern part of the country different from the southern part? Does it still have an impact?

**Interviewee:** The north of the country is different from the south economically, or whether it has had an impact—maybe it has some influence, but what is really important, as I mentioned, is the availability of necessary facilities. Poorer areas have fewer resources. Obviously, if people have more living facilities and better access to appropriate resources, the likelihood of contracting this disease will be lower. The issue of water resources is one of the most important influencing factors. Agriculture is also a very important issue, and some specific occupations have a strong impact. For example, in the southern regions of the country, we frequently observed that one of the influencing factors in the growth and proliferation of this mosquito was the collection and storage of worn-out tires. Obviously, this might not exist in some provinces, but in the south, it is a serious issue. People need to be educated about how to store old tires, whether they should be stored at all or not. For example, even some specific occupations can be influential. For instance, we observed that the worn-out boats along the Persian Gulf and the Sea of Oman, if properly maintained, could have an impact on reducing mosquito abundance. That is, a fisherman could learn to properly store his boat when not in use and not leave it in coastal areas where water collects and mosquito breeding occurs. There are many social factors that are influential. Appropriate housing, proper use of cooling devices—all of these are factors that can have an impact. It differs from province to province. For each province, there should be precise risk mapping, and based on that risk mapping, decisions should be made about what actions should be taken, what the municipality should do, what the governorate should do, and what other organizations should do. How waste is collected, how sewage is managed—many of our cities, especially in southern provinces, do not have adequate waste collection and sewage systems. All of these are influential factors.

**Interviewer:** Thank you very much, Dr. You paid very good attention to the occupational issue and pointed it out. Thank you for your kindness. Thank you, Dr. I wanted to know your opinion regarding health and medical infrastructure. Is this also a factor that could influence the spread, if the health and medical infrastructure is stronger? Having more appropriate staff, meaning sufficient human resources, sufficient logistics?

**Interviewee:** One of the influential factors in controlling this disease is reducing the abundance of mosquitoes. Perhaps this is the most important influencing factor. Part of it relates to environmental improvement. Another part is direct control of the mosquito, especially during outbreaks of this disease. If a mistake occurs, if the approach is not scientific and precise, if we do not know what type of insecticide to use tomorrow, or where to use it, then excessive use of insecticides itself becomes one of the most important factors that, instead of benefiting, actually harms the disease control program. All of this requires a health system capable of educating the public and being effective, having experienced personnel who know what decisions to make and what actions to take at any moment.

**Interviewer:** Thank you very much, Dr. In your opinion, for controlling this disease, what health specialists are needed?

**Interviewee:** I think the most important specialists the country currently needs in the affected regions are entomology experts. These entomology experts can play the greatest role at the county level, of course with fully organized and scientific management, trained from a single source, provided with logistics, and given command authority. If anyone thinks they can act independently in controlling this disease or believes they know better than everyone else, know that they are on the wrong path. This is a long and heavy fight—the fight against **Aedes** mosquitoes will last for many years. Over these years, it is important that there is a cohesive national program for this issue, and that everyone participates, meaning both health care staff and the public. The people of the community have a very important role in education, awareness, environmental improvement, and self-care.

**Interviewer:** Thank you very much, I appreciate it. In your opinion, in this section, how should health policies and programs be implemented, and what should they pay attention to?

**Interviewee:** I did not fully understand your question.

**Interviewer:** My question is, now regarding health policies and programs that are influential, in your opinion, what should be considered, and what points should be taken into account, or do you have suggestions for the future?

**Interviewee:** The most important issue is that we put the experiences of successful countries side by side. There are countries that have had the greatest success in controlling the growth and proliferation of mosquitoes. In different parts of the world—from the Americas on one side to countries like Singapore and Thailand on the other side—each of them has experiences that should be studied and learned from. Secondly, we need an appropriate budget for this disease. An appropriate budget means a budget that, unlike the ones we are currently talking about, is actually allocated to the provinces of the country. Currently, it is so negligible that it should not even be counted on. This requires a very substantial budget. As I mentioned, it also requires public participation. A major part of control depends on people's behavior, education, and engagement. People need to understand its importance. If the public does not cooperate, rest assured, even the best countries in the world cannot overcome this disease without public participation. This is extremely important.

Fourthly, studies must be conducted in various health domains. What does this mean? It means studying human behaviors, which change over time. It also means studying mosquito resistance to insecticides, which changes and must be monitored. The issue of insecticide use in current conditions is so complex and calculated that it is limitless. It is not a matter for anyone to simply allow themselves to comment. This requires a very strong and capable research center that studies and provides its information to the country’s health authorities so they can plan effectively. Serological studies are extremely important for this issue. Strengthening diagnostic laboratories at both environmental and national levels is very, very important. Genomic information of the virus responsible for transmission is extremely important. These studies must be carried out continuously, without interruption, and the information must be constantly updated, along with many other factors that would really go beyond both my mind and your patience.

**Interviewer:** No, Dr., I am listening with all my heart and truly enjoying it. Thank you very much. I appreciate it. Regarding the strategies that can be used to control environmental, social, and economic factors, do you have any opinions?

**Interviewee:** Yes, my opinion is that the most important strategies are intersectoral coordination. The command to control this disease is a command that must primarily rely on political commitment. That is, senior officials at the national, provincial, and county levels must all contribute to ensuring that this program moves forward correctly. Without political support, the program will definitely fail. Do not doubt this. I know countries that, despite abundant wealth, failed at various times due to this lack of political commitment in control. Yes, that’s correct. I had to start from scratch. Yes, we understood. Yes, this is intersectoral cooperation. It is not enough for one sector to think it can act alone. Different sectors must fit together like pieces of a puzzle, use each other’s experiences, and exchange information with one another. This exchange of information is extremely influential. From my perspective, I think the Ministry of Interior and its colleagues have the greatest role in assisting the country’s health system to deal with this disease, which is a very, very serious disease. When it becomes widespread, we must be sure that everyone helps. Naturally, the organizations responsible for communication must use all updated information resources—like national TV, social media, virtual spaces. Social media, for example, acts like a double-edged sword in controlling this disease. Just as it can have a positive impact, if misused, it will certainly have a negative effect. This in itself is a highly influential factor.

Most importantly, I emphasize again—the issue of budget. Do not think that this disease can be controlled with the limited budgets currently available to the provinces. Alongside that is public participation. Many preventive measures against this disease are carried out by the people themselves. What does this mean? Just that people need to know how to use their air coolers so that water does not accumulate underneath the motor part. Why is this water collected there the perfect place for mosquito proliferation? Or when we talk about used tires, everyone thinks they know how to store them, but is it appropriate at all? How should it actually be done? Another issue I mentioned, especially in the southern regions, is becoming increasingly important day by day: the collection and storage of water in the small tanks placed on top of buildings. Proper management of these household water storage resources is one of the most important areas that, if done correctly, can significantly reduce mosquito proliferation.

**Interviewer:** Thank you very much, both points. Thank you for the insights you provided. Certainly, we are done now. If there is anything else you want to mention, I am at your service.

**Interviewee:** Anyway, forgive me if I spoke somewhat scattered.

**Interviewer:** No, please. I myself was unwell. It was very strange for me, but I couldn’t not respond. Hopefully, complete health will be restored. Your words are impactful.

**Interviewer:** Yes, Dr., it was definitely very useful. Thank you so much. You have worked very hard. Thank you again for giving me your time. Goodbye.

**1^st^ Focus group**

**Interviewer:** Considering the history I shared with you, these are the factors we have identified and the goal of the Ministry of Health, which says, for example, Dr. R, education, environmental improvement, and other interventions should not be overlooked. Education and environmental improvement. First education, then environmental improvement. We want to elaborate on this education a bit more. In fact, in our research work, these are not our personal opinions; we are only managing the process. The opinions are those of individuals like you, distinguished guests, which we simply organize and present. When we say education, in fact, what should this education look like? What methods should it have? How should the educational package be designed for it to be more effective?

**Dr. K:** Well, for Aedes, we have a program called Risk Communication and Community Engagement, or RCCE. We are implementing RCCE for it. The first step of this program is a situation analysis. That is, until we have a situation analysis from that city, from that university, or from that rural area affected by Aedes, we cannot carry out any interventions—especially educational interventions. It’s not enough for me to sit at the Ministry level and say, “Here is an educational pamphlet about Aedes; print 2,000 copies and distribute them among the people.” Perhaps the population I am targeting may be illiterate, and printed media may not be useful at all. Therefore, we wrote a general guideline for Aedes-transmitted diseases, like RCCE, and sent it to universities, asking them to implement this guideline according to their local conditions.

The first step is a situation analysis. The situation in Gilan is different from the situation in Chabahar, and naturally, their educational channels, educational interventions, beliefs, and even their languages are different. Therefore, we cannot have a single intervention for all universities; they want interventions tailored to their own situation analysis. For example, in Chabahar, in the area affected, one of the places involved is the Jangluk area. The team concluded that they should go door-to-door and provide face-to-face education using health volunteers and students from health-related fields. They identified this high-risk area. Considering that digital access and literacy were low there, face-to-face education was selected as an intervention and as their primary channel, using health volunteers to educate residents directly at their homes.

In general, the method of education can vary. We have thousands of educational channels: print media, national TV and radio, celebrities, and influential individuals. For example, an imam in a city or a village may be highly respected by the people, and we use this channel to deliver educational messages. Environmental advertising, urban television screens, and social media are also used. If we want to communicate with students, we use social media. All of these choices come from the situation analysis. Identifying target groups is one of the items that comes directly from the situation analysis at each university. Another major asset is schools and students, through which many children have worked successfully to convey educational messages to their parents. They even transmit environmental improvement messages to children.

Simultaneously, in every program being implemented, there is management of infodemics and rumors. If a false belief spreads among people or a rumor circulates, the team continuously monitors and responds to it. This is the routine practice in our universities.

In addition, the role of various organizations is crucial. We have a specific Aedes-focused committee for education, communications, and community engagement that brings partner organizations together. The team uses the educational capacity of all organizations to educate the community. For example, they hold meetings with the municipality to request specific billboards, coordinate with national TV for live programs, or use volunteers from the Red Crescent. Essentially, any organization involved in education and awareness is linked with our team, and they utilize all these capacities effectively.

**Interviewer:** The RCCE program you mentioned, its first step is the situation analysis. So, with the situation analysis, you probably, as you said, identify the target group, determine the severity of the situation there, and all of that… Then what do they do? Is there a second step?

**Dr. K:** Based on that situation analysis, we write the activities. From that, the target groups emerge. For each target group, a specific message is designed and delivered through its special communication channel. Then again, we complete the same questionnaire or situation analysis. We check what has changed before and after. For example, we are in weekly contact with universities. One of our universities last week reported that they conducted a situation analysis. People believed that the role of insecticides was much more important than, for example, installing screens on doors and windows. Then they worked a lot on these health messages for their target groups through various organizations, etc. Six months later, they conducted this evaluation again and saw that the awareness and behavior of people regarding the use of screens improved by around thirty to forty percent. This is the whole purpose of RCCE and our main goal in education, which is to proceed in a data-driven way, meaning not to deliver one message to the entire community but to tailor it so that our resources are not wasted.

**Interviewer:** I understand. Thank you, **Dr. K**. **Dr…. (male)**, do you have any comments?

**Interviewee 2:** Let me tell you that **Dr. K** has almost covered the main points. I will raise a topic, part of which is related to the discussion you mentioned. Usually, if we want to do an intervention mapping, we first conduct a needs assessment, draw a matrix, define our model, identify strategies, then design the program, implement it, and evaluate it. Since our topic here is specific, for example, in the southern regions of the country, Aedes may now be our first priority, if no specific case arises, we go to the matrix. The matrix we create usually works like this: behavioral objectives are defined, then individual and social determinants exist, and we specify for each objective which determinants apply. Based on the frequency, we determine which constructs from which models are useful for us, and the program is designed accordingly. This is the whole mental process, and I am not saying that all universities are proceeding based on this model. However, in the capacity-building workshops we hold for our colleagues, we try to instill this concept, and our colleagues have done this so much that their mental process follows this method. Therefore, the point is that each region, based on the conditions it has—**Dr. K** mentioned—and the analysis conducted, the matrix is drawn and may differ.

The next point is that the work we do, I step back one level. What we do first is networking people, and we have various programs in the office that perform this task. One of the most important programs is the National Self-Care Program. I mention this because later part of it will relate to you. You also get a perspective on what is happening in this office. In the Health Transformation Plan conducted in 2011, the work the office did was among the ten main programs of the Ministry, the National Self-Care Program. What did it do? This program had four main components. One component was individual self-care. What it did was extend our services to the level of health houses and health bases. One stage moved it further, bringing services into homes. How? One person from each family was designated as the family ambassador. They became our liaison with their family. Liaison means that it was their responsibility to receive a set of information and attend a series of courses or access some media, which they then transmitted to their family members. Thus, we now have a six-million-strong network of health ambassadors. I explained this networking for you besides its main function.

The second program is health volunteers.

**Interviewer:** Did you train the ambassadors in homes and health bases?

**Interviewee 2:** The point is that all our caregivers, at first, we were supposed to have a portal called the National Self-Care Portal. What was the duty of the National Self-Care Portal? Besides this in-person interaction, the in-person courses we held, its duty was that all health ambassadors would enter the portal, log in, and do self-reporting. For example, they would say, “I have diabetes,” or “My father has high blood pressure.” Based on the data entered, we would define a series of services for them. For example, we would say, “This media is related to your disease. These centers can provide services to you. You can receive these services for free.” And if, for instance, a woman was pregnant, specific services were defined for her, and notifications would be sent regarding which services on which day. Something like this. It is being worked on, but it is not yet available to the public.

Currently, the communication that health ambassadors have with our caregivers is through social media and in-person. Each health caregiver has formed a group, added health ambassadors as members, transmits all the data and information we provide, and holds meetings with them. They are in contact with the team they have as ambassadors. For us, I think there are a very large number, roughly fifty thousand virtual groups through which our health caregivers are in contact with their ambassadors via various social networks. This is individual self-care, and now it has other components as well. We have 400,000 health volunteers. Excuse me, neighborhood health volunteers. We have defined thirteen types of health volunteers, and we say that health ambassadors are also one type of health volunteer.

A neighborhood health volunteer is someone selected within the neighborhood, empowered, and covers twenty households now—we reduced their number from forty to twenty—maintains contact with them, and delivers a set of services.

**Interviewer:** Do you provide cascade training so that they go…?

**Interviewee 2:** Yes, exactly. And they provide a set of services. Therefore, we have 400,000 people who are health volunteers here. Besides their main function, also keep in mind the networking aspect.

We have self-help groups. We have fifty thousand self-help groups across the country, which are people who share a common issue. For example, diabetic individuals gather in a neighborhood, like TE groups, they gather together, do certain activities, and we act as facilitators alongside them. More importantly, there is the program for needs assessment and health promotion interventions.

What do we do? We—people, the team of officials and local trusted individuals—that is, all organizations present in the neighborhood and people trusted by the community and people whom the community refers to, along with the health team, meaning our specialized health team, sit together. These individuals together identify the priorities of that neighborhood, plan the interventions, and implement them. Three teams together. And this capacity that the Ministry of Health has—no other organization has this capacity to bring these three teams together. Many organizations go into neighborhoods and work, bring people, and bring trusted individuals. They do not have a specialized team in the neighborhood. The only organization that has a specialized team is the Ministry of Health, and this is very important because you said we are working on causes, we are identifying SDHs. This specialized team helps the team of trusted individuals and the community to guide them, provide them with the SDHs, and other points.

Therefore, these three teams come together, and certain things happen. Also, consider the networking here. So, there is a very large capacity of individuals who are connected and have formed a network.

If all these programs happen together in a neighborhood, we call it a health-promoting neighborhood. And if a city has these health-promoting neighborhoods present together, and the data is transferred to the governorate level, and the structure you see behind it is established, we call this a healthy city. This is the project that WHO is following. As long as we are serving you, two cities have joined the global network of healthy cities. About fourteen to fifteen cities have also volunteered. The rest are volunteering…

**Interviewer:** The base of the healthy city is…?

**Interviewee 2:** It has all these bases. Look, you cannot say I want to implement a healthy city program if it doesn’t have any of these. All of these are the base…

**Interviewer:** Meaning this organization and the teams, and…

**Interviewee 2:** This means a bottom-up process has been formed. You cannot say, “I will do the needs assessment, but I don’t want the network of people.” **Dr. Kh**’s problem with us was this. Look, we have a health team. Okay? We tell the health team to identify the priorities. They give us a list and tell the officials and trusted individuals, “You give us the list.” The same goes for the people. Okay?

So, how does our health team’s list come out? We tell them to examine all the indicators. They also have their own view of their environment and data. Based on health equity indicators, based on any upstream documents, they sit and make a list and identify what their priority is. Okay? Therefore, we didn’t limit them to just ten or fifteen health equity indicators. Instead, we gave them freedom, and based on the problems they perceived in their environment, the list emerges. Usually, the indicators are not outside the health system domain, and this is exactly the work **Dr. Kh** is doing. It’s exactly similar to our work. That is, the health equity indicators are right here. Part of our work is this.

Trusted individuals and officials are the same; FGDs are conducted, they are present, and they hold meetings together. Based on the amount of public engagement and the information they have, they create a list. The public is also surveyed; a sample from the community is taken. Our volunteers go door-to-door asking questions: “What is your problem? What about your family? What is happening in your neighborhood?” From this, a series of social determinants of health emerge, many of which we consider as causes rather than problems or topics.

**Interviewer:** Right, meaning there’s another root cause in it.

**Interviewee 2:** Yes, exactly. So, we extract these. Extracting them helps us solve our problems. I will explain to you.

Therefore, these three lists that emerge are combined with a formula to produce the final list for that center or health house, where priorities are determined. Based on these priorities, the county, university, and ministry make their decisions. This is the only bottom-up program where social listening happens, where data from the environment flows upward.

**Interviewer:** Excuse me, **Dr.**, I want to add something to your statements. Although it may slightly go off our current discussion, I’ll mention it. I think ultimately, even the program that wants to compile indicators eventually goes to the people. We say we must put some signals together and analyze them. That is, when we have rich information from surveys—or not exactly surveys, but our engagement with the people and the neighborhood—and we also look at the indicators from this side, if we put them together and analyze them, we can interpret and analyze them much more beautifully.

**Interviewee 2:** Where does the prioritization of your indicators come from? Tell me this.

**Interviewer:** In that project?

**Interviewee 2:** In that project at the environmental level, for example at the local health service center level.

**Interviewer:** For example, health indicators?

**Interviewee 2:** Who lists the health promotion indicators for you?

**Interviewer:** We specified a set of indicators…

**Interviewee 2:** A set of about ten to fifteen indicators has been specified. Among these… First, I believe it shouldn’t be limited. We are discussing this. Second, who lists them?

**Interviewer:** Based on the important indicators that are recognized internationally.

**Interviewee 2:** No, I mean, who actually lists them? Who says out of ten or eleven, this one…

**Interviewer:** It’s not one person; a team does this work.

**Interviewee 2:** Okay, so who is this team?

**Interviewer:** For example, from the Ministry of Health…

**Interviewee 2:** No, we cannot say the Ministry of Health…

**Interviewer:** For example, in Islamshahr, when we did it there, the colleagues who were in Islamshahr and knew their covered population…

**Interviewee 2:** **Dr.**, a program that wants to become national, when you have, for example, ten thousand centers, you cannot say that someone from the university level should go. At the environmental level, the people who are actually in the environment should carry it out themselves.

**Interviewer:** The assumption is that these indicators are not robust. That is, for each university or even each county, the indicators that are listed, reviewed, and need to be followed up are different, depending on the problems that particular city is facing. For example, colleagues from the Ministry of Health who selected twelve indicators for Islamshahr, we ultimately included in the protocol that these indicators could vary—for instance, if suicide has occurred in Islamshahr, which is very important, it must be considered. But perhaps in one of the counties of Yazd province, this indicator may not be relevant, may not need to be followed up. Another indicator might be more important.

**Interviewee 2:** This point is correct, but the problem is that this neighborhood’s issues differ from another neighborhood’s. You have one neighborhood on one side of the highway where the urban conditions are completely different. Take Farahzadi, for example. On one side of the highway are informal settlements, on the other side it’s recreational. The conditions are completely different. Therefore, you cannot say that in one neighborhood… We believe neighborhoods differ, and the health team that is based in that neighborhood can list this. So, we cannot dictate from the top…

**Interviewer:** Those indicators act like alarms. When we get the initial information from the centers, this helps with the issue you mentioned.

**Interviewee 2:** Now, we don’t care about critiquing this.

**Interviewer:** No, look, the indicators act as an alarm to help you, and the people help in understanding why this alarm exists.

**Interviewee 2:** But the people are not involved in prioritization, they are only involved in interventions. That is the main point. See, from the very beginning of determining priorities, the people are present with us.

**Interviewer:** Yes, **Dr.**, for example, where problematic indicators are identified, the fact that people are also interviewed when they are visiting…

**Interviewee 2:** No, for determining… in your program this isn’t the case; that is, in setting priorities, people are not present. You only consider that number based on the indicators. Now, I don’t care. Let me tell you that this is how the work is done. In the discussion behind Aedes, people’s networking is very important. That is, tomorrow if you want to empower people, it’s not that you just give them media. It’s not that, for example, only the national TV goes. You must have prepared some arrangements beforehand. You must have formed a network based on existing relationships. For example, the Health Promotion Council that we form in neighborhoods: people, trusted figures, and the health team come together. They have some communications, sit together, reach an agreement that the problem is behind Aedes, and when this happens, the effort and motivation exist to carry out certain actions. Therefore, you cannot say that from the top… this is happening, I’m not saying this is wrong. That is, the top-down process exists. It always has. The priority, for example, was COVID. Of course, the people’s needs must be understood, but in some places, people may really not have reached this understanding… But this readiness exists beforehand, and in some places, the neighborhood-centered approach can help. This is also part of the work that we consider; that is, this infrastructure exists, and we use this capacity so that…

Then, the point that **Interviewer** mentioned is exactly that: an analysis must be done to clearly determine that people, that is everyone, are present and help with…

The discussion of planning for interventions, that is… we create a program based on the priority that emerges. This is also an important point to tell you. It’s not just educational. That is, we work both on behavioral and non-behavioral aspects. Behavioral or educational: our strategy is education, using different things, different channels. Non-behavioral usually involves things that are not in our control. That is, they are non-educational. Here, organizations step forward, people step forward, and we determine that this activity is necessary to solve the problem. Who should do what, when, and how? For example, if the municipality has its representative in our council, responsibility is assigned to the municipality. If the Welfare Organization exists, if a school principal, if a clergyman exists, they are determined, a time frame is set, the program is arranged, and now… we have set a criterion to say whether the program was implemented correctly or not, and then reporting and evaluation are carried out. This is the general work that is done in this office in this way.

**Interviewer:** Very excellent. Thank you for your interesting explanations.

**Interviewee 2:** I was referring in part of my talk to something that helps us understand how we organize a program.

**Interviewer:** You very well pointed out the infrastructures, that these infrastructures exist so that, probably with those infrastructures, if the RCCE program is to be implemented properly, we can use them to advance the program. **Dr.**, you mentioned an issue regarding the use of media and now social networks… I have a question and I want to clarify this issue, especially regarding the Aedes mosquito. That is, whether the media—most importantly, for example, national TV—is motivated in a particular way or is it mandatory? How do you attract their participation so that this training is delivered to the people in their own voice? Or now regarding social networks? Well, one of the best platforms to deliver education to people is these social networks, provided, for example, that it’s a blogger or influencer. Someone who impacts people, has many followers, and they see your message. What did you do about this issue? Do you arrange to have a network with millions of audience so that people see it, like it, or if you don’t have it, do you use individuals who have that position? That person can be a celebrity or anyone else.

**Dr. K:** Look, each of our universities actually has a list of influential people and celebrities, as well as high-audience channels. For example, in Kangan, where we had an issue in Bushehr, there is a channel called Kangan News, where the whole city is subscribed, and our team communicated with this channel. They convey messages and health messages and ask them, if there is news or something new happens, to update their messages. So each university has its own list of influential people, high-audience channels, and high-audience news agencies, which, in crisis or non-crisis conditions, deliver messages as needed. How we bring national TV to the task…

**Interviewer:** Because it’s all pay-to-play with national TV…

**Dr. K:** Yes.

**Interviewee 2:** They are not like that with us.

**Interviewer:** So why are health messages and training so limited, **Dr.**?

**Interviewee 2:** Look, some of the services that national TV provides us are free. For example, subtitles are free. Okay? Some services have production costs on us, but we don’t pay for broadcasting. That is, if we have a ready clip, broadcasting it is free for us. But, for example, if an external company is involved, they charge a production fee to prepare the clip and a broadcasting fee, saying, for example, for one minute of airtime, give us this number. So these two numbers are separate. Production costs are on us, broadcasting costs are with…

**Interviewer:** For broadcasting, do they set limitations?

**Interviewee 2:** There is a limitation, but they cooperate with us a lot. It’s not like we hit a restriction.

**Dr. K:** But this again depends on the crisis conditions. For example, a crisis like COVID—since the National COVID Taskforce was chaired by the President, all these organizations were members, and national TV had assigned duties. As you saw, no matter which channel we turned to, they were giving messages about COVID. So this completely depends on the crisis conditions. If Aedes hasn’t become a national issue, then national TV won’t emphasize it much and instead relies on provincial networks. For example, now Hormozgan is affected. So the provincial TV of Hormozgan delivers messages about Aedes. This again depends on the crisis situation. For instance, Hormozgan doesn’t want to lose its tourists. So their TV doesn’t overly announce that, I don’t know, Kish is contaminated, and they don’t want to scare people. This is their policy decision. But whether we force them or not, we also have upstream documents. We have a national document. Civil defense has issued a bio-surveillance document about Aedes, which provides duties for all organizations. For example, if it says environmental improvement is required, the municipality is responsible for environmental improvement. Duties are assigned for all organizations. National TV has always been involved in education and cultural promotion. Whether I say they participate 100% or not is variable, depending on the situation, the crisis, the priority, and how sensitive the issue is.

**Interviewer:** Well, you said Hormozgan might not want to lose tourists. That’s an issue because if a little negligence happens there, other provinces are affected. They can’t make decisions on their own.

**Dr. K:** That’s something beyond the Ministry of Health. That is, we can only send a letter and ask them to do it. Whether they do it or not, they are not accountable to us; they are accountable to their upstream organization. For example, the Kish Free Zone really made things difficult for us for a while and didn’t allow us to announce that Kish had Aedes mosquitoes, because they didn’t want to lose their tourism industry. All of this is the reality. Now, the discussions we had may not relate much to public education but are more about policy, and in fact, the Deputy of Health is more involved.

**Interviewer:** That is, for example, now we have reached the point where the trainings are delivered through the media of that province.

**Dr. K:** I said, it’s participatory, variable, depending on the level of risk we are currently in.

**Interviewer:** Maybe right now that risk level hasn’t been perceived enough, so trainings aren’t emphasized. Could this be?

**Dr. K:** It’s possible. It’s very clear. Maybe even many of our officials, for example, the governor of a city that is full of Aedes mosquitoes, when he talks to the governor, he has no sense of risk at all and says, “Aedes mosquitoes aren’t my priority.”

**Interviewer:** Well, this training itself can create sensitivity, right **Dr.**?

**Dr. K:** Many things affect risk perception. What previous experience does that person have from a crisis? What priorities do they have now? Maybe their city is full of addiction, poverty, and unemployment, so Aedes mosquitoes may not seem significant to that governor. Addiction might seem more dangerous. Many factors influence individuals’ risk perception levels.

**Interviewer:** Yes, I mean that through training and raising awareness…

**Dr. K:** Definitely, you can increase it. We can definitely enhance risk perception. Just as is being done in the RCCE program. For example, people used to say, “It’s just a normal mosquito that poses no risk to us,” but with our trainings, the percentage of window screen installation in a city increases by forty percent. Definitely, if the trainings are done correctly, it leads to increased risk perception.

**Focus group 2**

**Interviewer:** Greetings to all the honorable attendees. As Dr. explained, following the meeting we had the day before yesterday, we are here so that we can first introduce ourselves. We have already done that introduction. My colleagues Mr. Jafarzadeh and Dr. Bakhtiari are my colleagues at the center and are among the main team members who carry out the study work and analyze them. .... In this meeting we actually want — I’ll give a general overview. Based on the reviews we have conducted, we have identified a list of social factors that affect Aedes mosquito–transmitted diseases, and of course you are experts in this field and very likely have full technical mastery of the causes of these diseases and their modes of transmission and so on. I ask you now just to look at the issue through the lens and perspective of SDH (social determinants of health). In fact, the reason we — who you probably see as outsiders to the domain of vector control and entomology — have entered this field is because of that SDH dimension. For that reason, I again request that you keep reminding yourselves to look at the issue from the perspective of which social factors affect mosquito-borne diseases and the mosquitoes themselves.

Overall, in the studies — the presentation I gave you that day was the findings of the studies. Considering the explanations we gave and the discussions that took place, yesterday we mixed the results of interviews we had with experts who were also in the provinces with this study output so that the checklists we sent you would be a little more complete. In general, in these studies we identified environmental factors such as stagnant water; now stagnant water appears differently in Gilan Province. For example, rainwater collecting in a hole, in tree roots, and, I don’t know, in rice seedling beds and such gathers in one place — like in Bushehr and Hormozgan, old docks and boats and those things that they themselves were mentioning — and in Chabahar they emphasized movement a lot, and because it is coastal and the humidity and temperature are at levels suitable for the growth of this mosquito, these things are influential. And broadly speaking … gender issues, age and education, and especially housing quality, poverty and income, and level of education — all of these can together influence both becoming infected with the disease and its exacerbation and the negative consequences it has.

This was the summary we arrived at, and we wanted in this meeting to prioritize these factors. But based on your expert opinions we want to conduct more interviews and enrich this list through sessions like this and a series of interviews. So, with your permission, we will record this meeting so that we have the discussions to analyze afterwards. We want you to answer a few questions. I’ll ask the questions now and then we will discuss them one by one. The questions we want to ask are: one is to discuss in general which socio-economic factors, in your view, can affect these mosquito-borne diseases and the mosquitoes themselves? Dr. first guided that the mosquitoes themselves are of two different types which could — you can look at this issue such that perhaps the factors affecting these two mosquito types are different, and you can discuss them separately. The next question I want to ask is whether, in your opinion, the report we want to prepare should generally talk about these factors, or whether what we want to present and the discussions you are having here now should address each province separately and speak for each setting individually — and God willing, if time permits, from your point of view what are the best intersectoral and SDH-focused interventions that can be applied to control these vectors and, both intersectorally and within the health sector itself, what interventions exist?

Given that Dr. said we can be here until 9 o’clock, if you honorable participants allow, we will start with Dr. on the first question, which is: in your opinion, what socio-economic factors influence this issue? You can give your explanations generally and broken down by province or by mosquito type.

**Interviewee:** What I’m saying, in any case — I’m not talking about global experience, these are my own observations over the years — is that what is very, very, very important is: 1) the economic conditions of the area where Aedes is going to appear. The general literacy level of the people is very important. Whether laws have been put in place beforehand to organize appropriate urban infrastructure — these things are very important. What does that mean? It means whether, for example, a city has been planned in advance under a municipal management plan or not — in its structure. Okay? On the other hand, whether service infrastructures for that area have been provided or not. For example, whether further development in the provision of drinking water has been planned in advance or not. I’m listing these factors and then you can, according to them, look at those things that are called SDH. Sorry I can’t organize exactly where to start right now. This is very important. This is very important. So we actually have two phases. One phase is when nothing has happened here yet. What factors make this place vulnerable? Okay? This goes back to how we have laid out the city beforehand. So one part goes back to the regulations and laws that were put in place earlier regarding provision of appropriate infrastructure — in different sectors from health to water. That’s one part.

The second part is how much the people in that area are connected with infected/contaminated areas. This is also very important. That is, how much that city or area is connected with infected countries? In fact, the degree of connection with endemic areas is a determining factor. Also in this part, whether there are deterrent or strict laws in terms of trade and movement — these are very important in that area. How much movement is legal, how much is illegal, how much is registered, how much is unregistered. These are actually among the factors that pertain to the area before we face a crisis. Now, the household income level is very important — the household’s economic income.

**Interviewer:** How does it affect things, in your view?

**Interviewee:** Look — sometimes I know something. For example, I know, suppose this water tank is broken and needs to be repaired but I can’t repair it because I don’t have the money. I don’t have the money. No matter how much you, as health workers, tell me, I fully understand it, but it can’t be done. So from that perspective it’s very important. That is, how much the household income is matters a lot.

The issue of literacy, in my view, is very important. Why? Because those who study more — that’s what I’ve noticed; of course it depends. A place like Chabahar may really not follow that formula much. The reason is that Chabahar is a small city. Overall it has very weak infrastructure. I mean, you can’t say the whole city has an uptown and a downtown, you know what I mean? Unless you separate a part called the free zone from the city. The rest of the city is similar everywhere — there’s nothing particularly different. You see the dispersion. But overall you would expect that in a place where the general literacy level is higher, this problem would be less.

I mentioned the income issue too. The question of access to social service centers — how widespread they are in a city — is, in my opinion, very important. Because sometimes many people who [unintelligible] simply cannot go to a center due to lack of access to these infrastructures. Now whether it’s about education — there’s the education issue — and also the question of benefiting from medical services, and how much development of healthcare infrastructure we have in the area is again very important.

In my opinion these two or three factors are very important. The rate of movement/traffic, the volume of trade with other countries and how much oversight there is over them, the general literacy level of the public, and people’s monthly incomes — these are all very important. That’s what came to my mind.

**Interviewer:** Okay, elaborate on interventions as well. In your opinion, what SDH-focused interventions could there be?

**Interviewee:** In any case, we must raise the general public’s knowledge. This is very important. In my view, the matter of provision — that is, in practice we must somehow improve people’s economic conditions. The most important part of the work is that the organizations involved in service delivery should strengthen the [unintelligible] aspect in this field and, in fact, therefore provide: 1) public education — this is very important. 2) And what may be no less important than that education is the improvement of people’s economic conditions in that area, and the provision of infrastructures related to this program. These are among the most important things.

**Interviewer:** Sir, in your view — regarding the report we plan to present to you — should our final synthesis be an overall/general summary, or should it remain structured the same way by province?

**Interviewee:** Look — overall we definitely want something for the whole country. But I think some places may have somewhat more special conditions, especially in border provinces that have particular circumstances. For example, the border in Sistan and Baluchestan certainly differs from the border with Azerbaijan. It is much weaker in terms of the infrastructure issues I mentioned to you, and in terms of general literacy. I think we should produce a single, general version for the whole country, but highlight some places and define specific interventions for them — for example Sistan and Baluchestan or even Hormozgan. Hormozgan’s conditions also differ from many other places, and from Sistan and Baluchestan.

**Interviewer:** And regarding that checklist we sent you — do you have any opinions on the criteria we included?

**Interviewee:** I reviewed it. Nothing particular came to my mind. In my opinion, it was appropriate. Just one thing — yesterday Ms. M pointed something out. I would say, since you went to the trouble of defining the domains and separating them, because the issue of *albopictus* is somewhat different — what I mentioned to you was about *aegypti*. It might be much better if, as Ms. M. suggested, you also separate that part of Aedes — separate *albopictus*. You see, *albopictus* is not as strongly linked with SDH factors in the way *aegypti* is, in terms of the interventions we define. Not to that degree. Perhaps, perhaps it relates more to the structures that provide services than to the communities and populations living there. More of that … because *albopictus* is not so dependent on the issues I mentioned. Not as much. Now, it does have some fine points, which I’m sure colleagues will explain.

**Interviewer:** Which one of you, esteemed colleagues? Well then, I’ll change the question. Dr. R, please go ahead.

**Dr. R:** Look, all vector-borne diseases — in a way — are locked together with poverty. The more poverty there is, the more diseases there are. And the more these diseases exist, the deeper poverty becomes. These two go hand in hand. So probably everything that is required for SDH stems from this point in our minds. We already know it’s tied to the socio-economic conditions of people, communities, regions, and countries.

When you look at Bangladesh, you understand why the severity of disease there is worse than in India. In India, the socio-economic situation isn’t like Bangladesh’s. Yes, the population is high. Yes, there is visible poverty. But it’s never like Bangladesh. Move a little farther, to the borders of Malaysia and Thailand — there the disease is present, but its severity and impact on the economy, on holding back society, on mortality rates — it’s not as severe as in Bangladesh or Pakistan. So that’s a very broad overview.

Now, the disease has even reached Europe. But in many parts of Europe, when you ask people whether dengue fever is a problem here, or whether *Aedes* is an important mosquito here, they don’t know what you’re talking about. Because development is doing its job. The mosquito has arrived, yes — but people don’t feel it. *[****Interviewer:*** *They don’t sense it.]* Exactly — ordinary people don’t sense it at all. I once asked in northern Italy — an area with many rice paddies — regular citizens how much they knew about dengue or *Aedes*. They said they hadn’t heard much. Meanwhile, a specialist there — even an Iranian — works specifically on *Aedes albopictus* with the municipality. But the average person doesn’t know, because development has kept the problem at bay.

Dr. Z believes that the disease has even reached halfway into Switzerland. In summer 2024, reports even mentioned outbreaks in parts of France. But is what happened in France anything like what happens in Bangladesh — where WHO issues statements that so many people were infected, so many died — and those who died were the poorest? No, it’s not comparable.

So what I’m saying is that the disease is strongly linked with the socio-economic status of people, countries, and social groups. One of the clearest components of SES — after monthly income — is literacy. Unfortunately, those who are less privileged are also less educated. A family with higher SES may have educated members who can easily understand many factors related to *Aedes*, the disease’s transmission, and apply control measures within their means.

The simplest example: regularly emptying the water in flowerpot saucers. An educated person will definitely do this. But someone less educated — their split-unit AC pipe is constantly dripping water outside in Chabahar, creating standing water. They may not even have flowerpots, but they’re still producing breeding sites. That’s why literacy is so important.

Worldwide studies have shown that combining literacy with community awareness and environmental sanitation works best. For example, a study in Havana, Cuba in the 1970s found that removing insecticides and instead educating people had a far greater impact than insecticides alone in uninformed communities. This shows that public awareness really matters, and awareness is inseparable from socio-economic status. The poorer often have less education, and those with higher SES usually have more.

There’s also a very important issue in southern Iran that perhaps hasn’t been widely discussed internationally: elevator shafts. In Bandar Abbas and Chabahar, elevator shafts are a huge problem. Why? Because during construction, the elevator pit is used as a water reservoir. Since freshwater is scarce and expensive, builders store it there. After construction, it becomes the base of the elevator system. But while under construction, it’s a perfect pool for breeding mosquitoes. It’s cool, damp, at the lowest level of the building. Poor construction workers — often Afghans or impoverished Iranians — rest there in the summer heat, lying near the water. This greatly increases human-vector contact.

If in Chabahar and Bandar Abbas we could properly manage this issue — for example, through the Engineering Organization mandating that such practices stop, even halting construction until resolved — then we would eliminate a key SDH-related factor. Addressing it as part of SDH interventions would be very effective.

That’s all I have to add. I would really like to hear my colleagues’ thoughts as well.

**Interviewer:** We benefitted from your insights. Thank you. Our colleagues would also like to speak.

**Interviewee 2:** Everything that needed to be said has already been mentioned. The only thing that comes to my mind is uncontrolled urban development. This really leads to major problems when it happens without the necessary infrastructure. I think this is one of the factors that can cause…

**Interviewer:** So, you mean urbanization has an impact on…?

**Interviewee 2:** Uncontrolled urban development without infrastructure, and the issue of informal settlements.

**Interviewer:** Informal settlements and marginalized populations—probably because they lack proper urban infrastructure. In fact, one of the factors we identified was exactly this issue: that in informal settlements, water systems aren’t adequate, so people are forced to store water in containers or cisterns, or their wastewater disposal is improper. These become suitable sources for the problem. Thank you.

**Dr. M:** Dr. R made very valuable points. Now, I’d like to restate some of them in my own words. One issue is again the difference between *aegypti* and *albopictus*. *Aegypti* is more connected to people and their way of life, while *albopictus* less so. For example, *albopictus* may be found in water under flowerpots—in a place like the north, where everyone has flowerpots, water under them can harbor larvae. This happens regardless of whether people are well-off or not.

Another example is tree holes: anywhere water can collect, whether from rainfall or condensation. *Albopictus* prefers clean water. For instance, here’s a sample of a tree hole where *Aedes* was captured [shows image]. So, condensation creates water, and larvae can be found inside. That means distribution is tied to trees of this type—or plants like yucca, which have palm-like leaves forming funnel shapes that trap water. These are widespread. Therefore, its dependency on socioeconomic conditions may not be as strong—or perhaps it is, depending on what your studies ultimately show. These are just my assumptions.

On the other hand, with *aegypti*, for example in Chabahar, we found the highest abundance of mosquitoes in areas linked to water storage containers. Now, whether people can afford proper storage containers depends on poverty and income. If they can’t afford them, sometimes charities distribute containers. But then comes the question: do people maintain them properly? Do they keep the lids tightly closed? Do they eliminate other containers, or keep several more besides this one?

Also, Dr. N, the last time she went, was supposed to conduct another round of analysis to check whether it’s really water from air conditioner drains—or whether that may have changed over time. Because of course, it can vary depending on the season.

**Interviewer:** Both of these were mentioned to us by the provincial colleagues. I don’t remember if they referred to air conditioner drains, elevator pits, or water storage containers in Chabahar…

**Dr. M:** In Chabahar and many other southern areas, anywhere there’s drought, this can happen. Even in places that might be well-established and affluent, if there’s no water due to drought, people may still need to store water in some way. And then, infrastructure becomes really important. Sometimes a government or developer plans a small town, considering water, sewage, and all of that in advance. But sometimes people just settle somewhere — like in many parts of Chabahar, where there’s informal settlement, as Ms. Elhamzadeh mentioned, the city expanded in this way — with no infrastructure. Even when the government tries to plan a city thoughtfully, in practice you see that the theory doesn’t always match reality: slopes aren’t correct, drainage isn’t proper, gutters are inadequate, supervision is lacking. So intersectoral interventions for infrastructure are very important.

Now, the public literacy level — obviously, in everything I always say three times: education, education, education. I always say this, perhaps for any task. Be it mosquito bite prevention, timely hospital visits to remove viral reservoirs, identifying and eliminating cases early, treatment to prevent complications — yes, these are all very important. This education also applies to workers laying sewage pipes, water pipes, drainage, gutters — it all comes back to training. Education is critical for everything.

The sustainability of infrastructure also depends on the government. That ties back to overall socio-economic status. Infrastructure may exist but not be sustainable: pipes may break because the wrong materials are used or proper techniques aren’t applied. These factors also have an impact.

Then, water under air conditioners may exist in some places. With seasonal fluctuations in mosquito abundance, we’ve observed zero mosquitoes in certain spots. One colleague from Chabahar asked why we emphasized tires, yet mosquito abundance in tires was zero. But in water storage containers, it was different. We also considered rainfall and historical data — after each rainfall, water didn’t accumulate in tires anymore.

**Interviewer:** The tires — at the docks?

**Dr. M:** No, I mean used tires. In some places in Chabahar, they’re even used as house walls or fences — you practically don’t see a proper wall in marginalized areas. But in other places, they sell them. In areas without walls, some people collect these tires as waste and keep them. They’re plastic — old plastics collected and set aside. This also ties to poverty, as people collect them to sell for income. Normally, tires don’t hold water. In those areas, with the drought, mosquito abundance was zero — larvae weren’t found in the tires. But after a rainfall, larval abundance surged — the larval count in tires spiked, even several times higher than in water storage containers.

**Interviewer:** So, the material of the tires — does it favor mosquito growth?

**Interviewee 2:** Look, these are mostly in the outskirts of cities, used as house walls. Rainwater collects in the tire, the volume is small and clean, and the black color of the tire is attractive to mosquitoes. It becomes an ideal habitat for laying eggs.

**Dr. M:** The mosquito is most attracted to black, and it also likes the smell of tires. Many infestations in European countries happen through imported tires. When mosquitoes lay eggs in a tire, the eggs remain there, resistant to drying. They can stay for months, and with the first rainfall, the eggs hatch into larvae and the life cycle begins. So even now, when we talk about water storage containers, we have to compare them with rainfall. In my opinion, based on the data the team has collected so far, tires are still very different from all the other breeding sites.

**Interviewer 2:** Excuse me, a question came to mind: are the eggs resistant to spraying and chemical treatments?

**Interviewee:** The eggs have a shell that prevents chemicals from affecting them. That’s why all the control measures we implement target the larvae and adult mosquitoes.

**Dr. M:** Yes. But for example, if we physically spray diesel — well, these eggs have respiratory openings that can get blocked.

**Dr. R:** Or you could put them in conditions where, for example, some disinfectants at very high doses are enclosed with them, releasing the gases — that might kill them. But using insecticides isn’t a routine method for combating eggs. What mostly gets transported are the eggs themselves, moving between countries, regions, and cities. The adult mosquito can’t fly that distance. Mosquitoes might also be transported via vehicles, but their lifespan is short, whereas the eggs can survive much longer — up to around 400 days, for example, tolerating dry conditions.

**Interviewee :** Just one more very important point — Dr. Mashaikhi went into the entomology section — is that an adult mosquito infected with the virus can also transmit it to its subsequent generations.

**Dr. M:** The eggs they lay are also infected with the virus. The contamination in Europe happened through this egg transmission — those tires imported for trade, or even the tires used as bumpers on ships, might have come from a country that was already infected. Then the mosquitoes lay eggs in them, which are transported to the destination country. Even if the seawater is salty, or if the tires haven’t experienced rainfall during transport, once at the destination a single rainfall can trigger the mosquito’s life cycle, and the adult mosquito emerges.

As Ms. M mentioned, for example, we now have *albopictus* in the north, but since the mosquitoes weren’t infected, we haven’t had any local transmission cases. However, if an infected mosquito arrives, or a reservoir containing the virus reaches that area and a mosquito feeds on it, the mosquito becomes infected — and the infection persists. This means it can be transmitted to subsequent generations; the virus can pass from generation to generation.

**Interviewer:** But they might not get sick, right? I mean, the one who…

**Dr. M:** If an infected mosquito bites someone, that person *might*… but the disease aspect is a separate issue. Only about 20% show symptoms the first time. Now, Dr. **Interviewer**, I think this might not be directly related later, but let me continue. I was talking about water under air conditioners. Even in a very affluent area, if people can’t manage the water under their air conditioners, it accumulates. [**Interviewer:** Yes, I understand.] This water under the AC can still become a suitable site for larvae. Even if people are wealthy, if they lack health literacy and awareness, they won’t be able to manage this water properly…

**Interviewer:** For example, in Ahvaz, at my father’s house, they just drain this water with a hose…

**Dr. R:** Usually they take it to the garden or collect it in a container until it’s full…

**Interviewee 2:** With a lid on it. Unless it’s emptied regularly, the mosquitoes won’t develop in it.

**Dr. M:** At most, five days. But the best method is to open the lid and, for example, direct the water through a pipe into the garden soil.

**Interviewer:** Now, this issue regarding water is very important. Dr., please go ahead.

**Interviewee 3:** I think almost all the main points have been covered — poverty, education, health literacy, as other professors mentioned. I just want to briefly touch on culture and subcultures in different areas. For instance, language and how education is delivered are extremely important.

In some areas, particularly in Baluchistan, you can see that even if economic conditions are relatively better in certain households, subcultural practices shaped by upbringing and social norms still affect behavior. For example, hygiene — both personal and public — may not be properly observed, regardless of economic status.

I’ll give an example that might not be directly related to *Aedes* mosquitoes or dengue fever but may make the point more tangible. Over 20 years ago, in a village in Nikshahr County, someone had built a very large house — I think it cost about 700 million tomans back then. It was essentially a mansion in the village. Yet, when this person needed to use the toilet, they would go about 100 meters away into the open and use the ground, cleaning themselves with clay. My point is that these subcultural practices must be considered when addressing areas where issues exist. Even if someone is educated, their upbringing may influence behaviors in ways that affect health outcomes.

So, culture can have a strong impact. The language used in educational programs must be carefully considered, especially in southern and northern regions where local languages are prevalent.

Another point is that, in my view, the more deprived a province is, the weaker even the governmental infrastructure tends to be.

**Interviewer:** Because their level of advocacy and demand for services is lower.

**Interviewee 3:** Exactly. The more deprived the population, the more basic challenges and daily struggles they face, which limits their ability to advocate for themselves. For example, regarding *Aedes* mosquitoes, the idea that people should improve the sanitation around their own homes is correct in theory — many of our issues could be resolved this way.

However, in more deprived areas, even if everyone maintains their own home properly, if the broader infrastructure and development — as Dr. Nourollahi mentioned — are lacking, the impact is limited, and we may not achieve our primary goals.

Why is that? For instance, in Konarak, someone might say, “There’s only one garbage collection truck in the entire county, and it breaks down every other day.” So, governmental infrastructure in deprived areas is weaker, and a major reason is the lack of advocacy from local authorities. This issue also needs to be addressed in parallel with other interventions. We cannot focus on one issue from start to finish while neglecting others; otherwise, we may fail to achieve meaningful outcomes.

The other professors have covered the remaining points much better than I could, so I won’t take more of the session’s time. These were just the two points I thought should be added.

**Dr. M:** Let me mention two more points. First, culture is another factor. People may be poor, but their religious or ethnic culture may make them more likely to follow guidance from a local cleric or elder. For example, in Sirik, after mosquitoes were caught, the local Sunni population followed instructions from their *Moulavi* regarding what to do with water containers. This cultural aspect can be very helpful.

Second, in my view, the link between socio-economic factors and mosquito abundance is stronger than with the disease itself. This is because *Aedes* mosquitoes are daytime biters — their peak biting occurs after sunrise and before sunset, for example between 6:30–8:30 a.m. or 4–6 p.m. So, even someone with a well-maintained home could get bitten if they go to a football field or another area where poorer communities live, or vice versa. Therefore, the places people work, eat, or visit must also be considered in interventions.

**Interviewer:** Dr. R, in your remarks you were kind of referring to interventions at the same time as highlighting factors, and one key point you emphasized was education. Education itself, as an intervention, ensures that people carry out other preventive measures themselves. Now, particularly regarding house sanitation or stagnant water at their homes and related issues, education has been highlighted as very important.

But, for example, you just mentioned that a water storage container should have a cover or be treated in a certain way. How should educational interventions be designed so that the message reaches the audience, they actually learn it, and understand well enough to implement these practices? We are already confident that education is crucial, but in terms of *how* it should be delivered, what educational packages or intervention designs would be most effective?

**Dr. R:** In my view, colleagues who are specialists in education—those with expertise and practical experience—are better positioned to comment on the *how* of educational interventions. However, in the high-risk target areas for dengue and Aedes-borne diseases, it seems that face-to-face education will be essential. Naturally, we don’t have the manpower or resources to deliver all of this ourselves.

If we can invest in volunteers or community representatives, these individuals can deliver face-to-face, “from-person-to-person” education within their own communities. Volunteers could play a very significant role in this approach.

We have experience from malaria programs in the same regions. In rural Sunni populations, the influence of community religious leaders—such as the *pishnamaz*—can be very effective. For example, a laborer may work in the morning, and then the pishnamaz gives the call to prayer at the mosque, and people follow him. Their guidance can move the community to action. This differs from more formal religious authorities (*muftis*), whose instructions are almost always followed; pishnamaz in rural areas doesn’t necessarily have a formal role, but their advice still carries weight.

For example, we successfully used a larvicide powder or granule to control *Anopheles* larvae in southern Sistan and Baluchestan. Community members would apply it once a week to their small water storage containers—about the size of a soda bottle cap—without needing to identify larvae, monitor them, or check the process. The practice was widely adopted because it was simple, mechanical, and didn’t require specialized knowledge.

I want to emphasize that face-to-face education is highly effective, but it demands significant human resources. Since we don’t have enough personnel, leveraging volunteers is the most efficient strategy. For dengue prevention and related interventions, building the program around trained community volunteers seems to be the most sustainable and practical approach.

**Interviewer 3:** What about organizational training? For example, when we were reviewing interventions in other countries, one example was a country where the organization responsible for water supply in affected areas trained its staff so that they could manage the water storage cycle for the community.

**Dr. R:** Fortunately, fortunately, in our governmental structure and organogram, there’s a kind of mandatory basis—so, for example, you can call a meeting with the heads of administrative councils, and we raise the issue. It’s not that simple, of course. We draft a formal report and get their signatures. In the next step, an expert trains their staff, and then through a monitoring mechanism, you ensure that what was planned is actually being implemented. Fortunately, in our society, for the government sector and public employees in other agencies, training is easier than anything else. It has been done—fortunately, many government employees in the southern regions of the country are well familiar with Aedes mosquitoes. Of course, the public also knows about them, but the staff know this issue very well.

**Interviewer:** Colleagues, regarding interventions… not just educational ones. Since Dr. R emphasized this, I wanted us to expand a bit more. His point was very important: if we want to focus, we shouldn’t limit ourselves to just education. We should speak more specifically. I think in the focus group session with our health education colleagues, we’ll discuss this issue in more detail. Given the discussion we’ve had so far, the factors are now fairly clear. We’ll discuss these factors in relation to educational interventions—how they should be designed and implemented.

Now, considering the challenges we discussed in the first round—such as house improvements, stagnant water, and urban infrastructure—regarding interventions that you think would be most effective: the range of possible interventions is wide, but there may be a few key interventions with the greatest impact. For example, as Dr. M. mentioned, the issue with tires has a much higher coverage rate. So perhaps the intervention should focus on this specifically, while other things with less impact might be secondary.

What interventions should be prioritized? In other words, which interventions should be placed on the current agenda for maximum effectiveness?

**Dr. R:** Interviewer, with all due respect, on the contrary—the interventions are actually very limited. Interventions for controlling the invasive Aedes mosquito, which transmits dengue fever, are quite restricted. When we talk about environmental improvement, its scope is clear and well-defined.

**Interviewer:** So when I said “a wide range,” you’re saying it’s not really wide?

**Dr. R:** No, it really isn’t broad. Environmental improvement starts at the household level, then extends to small workshops or slightly larger production complexes, and at most to the community. It’s very, very limited. Fortunately, if we do environmental improvement and education well, the world has shown that everything can be managed. Singapore is a very successful model.

If we try to broaden it too much, we don’t want you to go too far because those factors are no longer really SDH—they aren’t social determinants. They aren’t the socio-economic factors of a community anymore. For example, some advanced countries use a method called SIT, but that’s not SDH—you need money and scientific knowledge to do it. I don’t want to distract your thinking. Fortunately, the ways to combat Aedes-borne diseases are very clear. Environmental improvement means that, for instance, a military organization could manage its housing camps, remove stagnant water, fill holes, and eliminate places where rainwater accumulates.

From there, scaling up to the city level involves collecting even small things, like an old tin can where rainwater has gathered. It’s the same category. If you compare it to a disease like COVID, you would say: “People must wear masks, healthcare workers wear gloves, and ordinary people should avoid risky places.” There are many categories of activities for respiratory disease. But for vector-borne diseases, fortunately, it’s limited. That makes our role more feasible. There are essentially just these two categories.

**Interviewer:** Dr. R, could you give a concise summary of the interventions implemented in the country, if you recall them?

**Interviewer 3:** Usually, interventions that focus on only one aspect cannot succeed. They need to be combined—both controlling the vector **and** educating the community. Nowhere has it been seen that a single intervention targeting one factor alone can achieve control. The combination should include community education, training of healthcare personnel, and interventions based on mosquito genetics—which, by the way, are **not** SDH.

**Interviewer:** Do the colleagues here agree with Dr. R’s views?

**Interviewee 2:** Actually, I think education is very effective, but it has to involve practice and repetition. For raising awareness, someone might know that even very small, easily overlooked water sources can serve as breeding sites. But just knowing that isn’t enough—they haven’t fully internalized it, so the behavior change doesn’t happen. That’s very important. One-off or sporadic trainings are not very effective. Good education should not only increase awareness but also lead to behavior change. I think this is very impactful in our discussion, especially regarding environmental management, as Dr. R mentioned.

**Interviewer 3:** Just regarding… I was reviewing national interventions, and I noticed that while they identified areas already affected, neighboring areas that are at risk weren’t well defined. What should be done to prevent spread to those areas?

**Dr. R:** Actually, if they hadn’t done it, we did. For example, in those top-right maps, the areas marked in red aren’t currently affected. But we indicated them because, in terms of habitat and ecological suitability, they are at risk. For instance, Khuzestan is marked red even though we currently have no cases there. We marked it to show that in the future, these areas could become suitable for transmission.

**Interviewer 3:** So interventions should be applied there simultaneously as well?

**Dr. R:** No, trapping and interventions—not there. Those aren’t actual interventions; they’re essentially early warning methods. For example, the map I’m showing you in yellow—everything marked there represents data from seven to ten years ago where entomological surveillance was conducted. At that time, there were no actual problems in the country, but entomological monitoring was taking place. From this perspective, we are not behind. We want something scientifically documented so that when we act, we can say it’s backed by a study conducted by an academic university group.

**Dr. M:** You see, to prevent a region from becoming infested, we carry out proactive measures. For example, previously, when ports weren’t yet infested, everyone was trained to ensure that incoming tires were stored under cover so that any eggs wouldn’t be exposed if it rained. These quarantine measures are theoretically defined, but just like in livestock control, they might not always be implemented due to various reasons. Movement from Chabahar to Konarak or further isn’t consistent, but entomological surveillance is conducted at entry points—if we detect a mosquito, vector control measures are implemented immediately. Trainings and awareness campaigns are provided to different groups. Overall, the national disease surveillance system covers both entomological and patient monitoring. For neighboring areas, surveillance is also established—for example, terminals, bus stops, or truck stops where vehicles from Afghanistan or Pakistan might bring mosquitoes. Entomological monitoring there allows for early intervention.

**Interviewer:** Do you want to add anything regarding interventions?
**Dr. M:** Interventions related to…
**Dr. R:** Environmental management, education. Environmental management. Education and environmental management.

{At the end, Dr. … mentioned that legal and regulatory measures should also be established in this regard.}

| **Identification Form for Social Determinants Affecting the Prevention, Control, and Spread of Aedes-Transmitted Diseases in Bushehr Province** | | | | | | | |
| --- | --- | --- | --- | --- | --- | --- | --- |
| **Identifying of Environmental Factors** | | | | | | | |
| What factors in the natural environment (climate, vegetation, stagnant water sources, water and sewage systems, urban design, waste management, temperature, humidity) contribute to the spread of Aedes mosquitoes in your province? | Climate, stagnant water sources, water and sewage systems, temperature, humidity | | | | | | |
| Does the type of housing in your province (building materials, urban structure, etc.) affect the issue under investigation? | No | | | | | | |
| Are there any stagnant water sources in your area? | Yes | | | | No | | |
| In your opinion, have climate changes had any impact? | Yes | No | | | | Somewhat | |
| Please list any other environmental factors in your province that have influenced the occurrence and spread of Aedes mosquito–borne diseases. If needed, describe how these factors have an impact. | Elevator shafts in buildings under construction containing water, and accumulated water in unused boats left in ports and docks. | | | | | | |
| Which organizations’ participation is key for environmental improvement in your province to ensure the success of related programs? | Governor’s office, Municipality, Provincial government | | | | | | |
| **Social and economic factors** | | | | | | | |
| Is the general public awareness in your province regarding the prevention methods of Aedes-transmitted diseases sufficient? | Yes | | No | | | | Somewhat |
| Is the level of awareness among relevant institutions, such as municipalities, appropriate? | Yes | | No | | | | Somewhat |
| Have travel and migration patterns affected the spread of Aedes mosquitoes? | Yes | | No | | | | Somewhat |
| Does poverty or lack of resources affect disease control? If so, how? | Yes, poverty has led to increased urban marginalization, and in these peripheral areas, the quality of life is generally lower. This situation creates numerous sites suitable for Aedes mosquito larvae to develop. | | | | | | |
| Which social factors (such as education level, occupation, culture, health literacy, etc.) play a role in the spread of the disease in your province? And which institutions’ participation is key to responding to this situation? | Culture and health literacy; national media (radio and television) and news agencies in cyberspace. | | | | | | |
| **Healthcare infrastructure** | | | | | | | |
| Are the existing healthcare and medical infrastructures sufficient? | Yes | | | No | | | |
| What are the main challenges related to healthcare and medical infrastructures regarding the prevention and control of Aedes-transmitted diseases in your province? |  | | | | | | |
| In your opinion, what policies or programs in the health and medical sector should be implemented? |  | | | | | | |
| **Experiences and recommendations** | | | | | | | |
| Has there been any successful experience in your region regarding the implementation of intersectoral interventions for controlling Aedes mosquitoes? Please explain. | Assigning a responsible authority for each task at the county level within the county governor’s office. Using the prosecutor’s orders for offenders. | | | | | | |
| In your opinion, what is the most effective strategy for controlling the vector and the diseases it transmits in your province? | Environmental improvement | | | | | | |
| **Additional explanations** | | | | | | | |
| Please write any points you think may be useful regarding this matter in this section. |  | | | | | | |

Form for Identifying Social Determinants Affecting the Prevention, Control, and Spread of Aedes Mosquito–Borne Diseases in Three Regions: Hormozgan, Gilan, and Chabahar

**Identifying Environmental Factors**

| What factors in the natural environment (climate, vegetation cover, stagnant water sources, water and sewage systems, urban design, waste management, temperature, humidity) play a role in the spread of Aedes mosquitoes in your province? | | | |
| --- | --- | --- | --- |
| **Hormozgan** | **Gilan** | **Chabahar** | **-** |
| - Must be amenable to human intervention - Environmental improvement - Reduction of stagnant water - Urgent intervention by urban water and sewage systems in city margins to reduce stagnant sewage water - Waste management - Water from broken pipes - Any open water sources, such as water under trees, water from air conditioners - Water remaining from rainfall in pits, abandoned containers, used tires - High humidity that prevents water sources from drying | - The amount of stagnant water in urban areas is very high, which has contributed to mosquito proliferation. - During the agricultural season, due to rice seedling irrigation, mosquito populations increase in rural areas because of stagnant water in rice paddies. - Presence of numerous wetlands. - In Gilan province, due to the existence of an urban sewage system, mosquito breeding in sewage is much lower than in southern provinces. - Urban and rural water collection systems: in urban areas, drainage channels are better designed and stagnant water is mostly absent; however, in rural areas, channels are generally stagnant, allowing mosquito oviposition. - Agricultural water transfer systems can also contribute due to stagnant water. | - Suitable temperature (annual average temperature 26°C) and humidity (60–70%). - Presence of stagnant water and wastewater in urban and peri-urban areas. - Lack of stable water storage in **hootak** (deep pits for water storage in villages). - Absence of a continuous urban water supply system (water available only 3–5 days per week). - Lack of an urban sewage collection system. - Urban infrastructure without drainage channels. |  |

| Does the type of housing in your province (building materials, urban texture, etc.) affect the issue under investigation? | | | |
| --- | --- | --- | --- |
| **Hormozgan** | **Gilan** | **Chabahar** | **-** |
| - Villa-type houses that have areas where water can accumulate (e.g., pools) - Apartment buildings (with balconies where potted plants are kept, or where air conditioner drip trays or other stagnant water sources exist) - Urban fabric that is traditional and old, with humid, semi-dark areas or environments with alternating shade and sunlight | - Wire and fabric screens in many homes - Population density in urbanized areas | - Older urban neighborhoods - Preference of local residents for building and living in villa-style houses |  |

| Are there any standing water sources in your area? | | | |
| --- | --- | --- | --- |
| **Hormozgan** | **Gilan** | **Chabahar** | **-** |
| Yes / No | Yes | Yes |  |

| In your opinion, have climate changes had any impact? | | | |
| --- | --- | --- | --- |
| **Hormozgan** | **Gilan** | **Chabahar** | **-** |
| Yes | No | Yes |  |

| Please list any other environmental factors in your province that have influenced the occurrence and spread of Aedes mosquito–borne diseases (if needed, describe how these factors have an impact). | | | |
| --- | --- | --- | --- |
| **Hormozgan** | **Gilan** | **Chabahar** | **-** |
| - Standing water in tires at docks with ships and boats - Water outlets or drainage channels along the coast - Abandoned or unused boats - Water storage containers from air conditioners in homes, markets, etc. - Buildings under construction that allow water to accumulate, such as elevator shafts - Old and traditional water reservoirs around cities like Khormoj and Bandar Lengeh - Factors such as the province receiving migrants from neighboring countries and provinces (Balochistan, Afghanistan, Pakistan, and Arab countries) - Global trade - Favorable weather conditions for mosquito growth and disease transmission for 10 months of the year | - | - Cross-border traffic to Pakistan and Arab countries in the Persian Gulf - Legal and illegal goods transit from affected countries - Lack of urban infrastructure and facilities in Chabahar - Economic and livelihood challenges of the population limiting preventive, control, and home-environment improvement measures - Presence of open water reservoirs on the city outskirts - Water storage in containers due to lack of a stable water supply in the city |  |

| Which organizations’ participation is key for environmental improvement in your province to ensure the success of related programs? | | | |
| --- | --- | --- | --- |
| **Hormozgan** | **Gilan** | **Chabahar** | **-** |
| - All governmental and private offices that have human resources, equipment, or machinery for this purpose - University of Medical Sciences - Governmental organizations such as the Governorate, County Governorships, District Offices, and Village Councils - Implementation of environmental improvement by municipalities - Water and Sewerage Department - Engineering Organization and Urban Housing/Planning - National media (IRIB) for public awareness - Education Department (Schools) - Road Administration - Oil, Gas, and Petrochemical Industries - Ports and Maritime Organization - Private construction companies | - Agricultural Jihad Organization: for spraying agricultural water transfer systems and educating farmers to improve rice seedling conditions in the fields - Health care centers, etc.: for installing wire screens - Municipalities and Village Councils: for controlling stagnant water in various urban and rural areas | - Ports and Maritime Organization - Free Trade Zone Authority - Fisheries Department |  |

**Social and economic factors**

| Is the general public in your province sufficiently aware of the ways to prevent diseases transmitted by Aedes mosquitoes? | | | |
| --- | --- | --- | --- |
| **Hormozgan** | **Gilan** | **Chabahar** | **-** |
| Yes / No | Somewhat | Somewhat |  |

| Is the level of awareness among relevant organizations, including municipalities, adequate? | | | |
| --- | --- | --- | --- |
| **Hormozgan** | **Gilan** | **Chabahar** | **-** |
| Yes / No / Somewhat | Somewhat | Yes |  |

| Have travel and migration patterns affected the spread of Aedes mosquitoes? | | | |
| --- | --- | --- | --- |
| **Hormozgan** | **Gilan** | **Chabahar** | **-** |
| Yes  (It is still not clear exactly how the mosquito entered Iran. It may have been present in low numbers for a long time and has now increased due to environmental conditions, or it might have entered Iran more recently due to climate changes.) | Yes | Yes |  |

| Does poverty or lack of resources affect disease control? If so, how? | | | |
| --- | --- | --- | --- |
| **Hormozgan** | **Gilan** | **Chabahar** | **-** |
| Yes   - In poor neighborhoods and households, environmental improvements are weaker. - Awareness and sensitivity of people toward *Aedes* mosquitoes are lower. - In impoverished areas, the likelihood of settlement by infected foreign nationals is higher. - Resource shortages in all sectors, such as lack of funding and human resources, affect both environmental measures (like habitat improvement) and health services (including dengue surveillance and control). - Primarily in developing countries, this disease is more prevalent due to issues such as poverty and insufficient control infrastructure, for example in Pakistan, Bangladesh, etc. | Yes  People’s inability or high cost of purchasing wire screens prevents them from keeping mosquitoes out of their homes. | A lot   - Living in huts, partially constructed block buildings, or unrenovated homes (lack of proper windows and screens, inadequate sewage systems, untreated wastewater, etc.) - Lack of cooling facilities and preference for resting in the yard - Reluctance to seek treatment due to healthcare costs |  |

| What social factors (such as education level, occupation, culture, health literacy, etc.) play a role in the spread of the disease in your province? And which organizations’ involvement is key to addressing this situation? | | | |
| --- | --- | --- | --- |
| **Hormozgan** | **Gilan** | **Chabahar** | **-** |
| - Education level - Cultural factors - Community health literacy and health promotion programs | Among social factors, occupation (agriculture) has the greatest impact in Gilan province. Educating farmers on spraying rice seedlings at the proper time, promoting health culture, and providing incentives can be highly significant. | Low general literacy in the region and reluctance to continue education due to high-income occupations (fishing and fuel smuggling).  Most residents engaged in self-employment and market-related jobs.  Average level of health literacy in the community. |  |

| Are the existing health and medical infrastructures sufficient? | | | |
| --- | --- | --- | --- |
| **Hormozgan** | **Gilan** | **Chabahar** | **-** |
| No | Yes | No |  |

| What are the main challenges related to health and medical infrastructure in your province regarding the prevention and control of Aedes-transmitted diseases? | | | |
| --- | --- | --- | --- |
| **Hormozgan** | **Gilan** | **Chabahar** | **-** |
| - Mostly related to the health deputy sector. - Lack of activity from other organizations in controlling mosquito abundance, such as municipalities. - Avoidance of responsibility for environmental sanitation. - Weakness of educational environments, such as schools and universities, in improving awareness and attitudes of the youth regarding Aedes mosquitoes and related diseases in the province. - Shortage of human resources. - Insufficient budget. - Delayed provision of diagnostic kits. - Lack of efficient administrative and managerial structures in this field. - Failure to employ specialists in vector control (graduates in biology and vector management). - Insufficient equipment for vector prevention and control. - Weak intersectoral collaboration among relevant organizations. | - Inability and high estimated cost for installing wire mesh screens in hospitals and health centers in the province. - Inability of the Health Deputy to fund spraying operations in high-risk areas. - Lack of cooperation from other organizations in controlling Aedes mosquitoes. | - Shortage of hospitals (in the southeastern region of the country, several counties are covered by a single 186-bed hospital). - Shortage of human resources (50% of organizational positions are vacant). |  |

| What policies or programs do you think should be implemented in the health and medical sector? | | | |
| --- | --- | --- | --- |
| **Hormozgan** | **Gilan** | **Chabahar** | **-** |
| - Developing a structured and continuous program with clearly assigned responsible authorities. - Strengthening the surveillance system, including increasing sampling of suspected cases and rapid identification of patients. - Enhancing and preparing hospital wards for the possibility of a widespread epidemic in the province. - Continuous education, prevention, control, and monitoring. | - Intersectoral coordination - Training individuals and target groups | - Establish 24-hour dengue surveillance centers at three locations in the city - Recruit dedicated staff for these centers (physicians, nurses, public health experts, laboratory technicians, etc.) |  |

**Experiences and recommendations**

| Is there a successful experience in your region regarding the implementation of intersectoral interventions for controlling Aedes mosquitoes? Please explain. | | | |
| --- | --- | --- | --- |
| **Hormozgan** | **Gilan** | **Chabahar** | **-** |
| The implementation of an intersectoral environmental improvement approach at the provincial level has been somewhat successful in reducing the abundance of Aedes mosquitoes. Other intersectoral actions, such as education programs based on risk communication and social participation to engage other organizations at the provincial level, are currently underway. Dengue case-finding activities and entomological surveillance have been effective in reducing both disease incidence and mosquito abundance.  Additionally, assessments of knowledge, attitudes, and practices of the general population in Hormozgan have been conducted through applied health research, with the participation of the Research Center for Social Determinants of Health, to improve preventive interventions.  Since I am not directly involved in operational work, I do not have precise information. However, the state media has been very active in this area. I myself have conducted two television interviews and two live radio programs on this topic. The Health Deputy regularly holds meetings with organizations such as the municipality and the media in this regard. | - | Interventions regarding education have begun in the city through volunteer teams, aiming to improve the health literacy level of the region. |  |

| In your opinion, what is the most effective strategy for controlling the vector and the diseases it transmits in your province? | | | |
| --- | --- | --- | --- |
| **Hormozgan** | **Gilan** | **Chabahar** | **-** |
| Strengthening the surveillance system, including increasing the number of suspected case samplings, rapid identification of patients, and reinforcing and preparing hospital wards for the possibility of a widespread epidemic in the province. Integrated vector management using specialized biologists and vector control experts to reduce disease incidence. | Spraying insecticide on stagnant water. | Environmental improvement to eliminate larval habitats Establishment of 24-hour surveillance centers for patient treatment and quarantine |  |

**Additional details / Further explanations**

| Please write here any additional points or comments that you think may be useful regarding this matter. | | | |
| --- | --- | --- | --- |
| **Hormozgan** | **Gilan** | **Chabahar** | **-** |
| At present, a dengue fever epidemic in the country is likely, as the number of cases in provinces such as Sistan and Baluchestan, Hormozgan, and others is increasing. For example, the sudden rise in cases in Chabahar serves as an early warning of a potential national epidemic.  Therefore, a nationwide intervention approach is necessary. Currently, the national guidelines based on biological defense interventions provide an appropriate framework for effective actions. It is also crucial to ensure both intersectoral and intrasectoral participation, along with continuous education of the public and authorities, while avoiding causing unnecessary concern in the community. | - | Securing funding and providing equipment for environmental improvement in the city of Chabahar.  Conducting public awareness and culture-building activities by IRIB and other organizations, including universities, the Ministry of Education, the Ministry of Culture and Islamic Guidance, the Islamic Propagation Organization, and municipalities, coordinated with county health center experts.  Establishing a field hospital in the free zone, along with provision of human resources, equipment, and medical supplies.  Restructuring hospitals and improving staff conditions and hospital infrastructure.  Preventing construction contractors from employing foreign laborers throughout Chabahar.  Identifying low-income households and leveraging charitable capacities to provide and deliver standard water storage tanks to these families, while obligating other households to cover their water cisterns. |  |
